# Insights into the pro-angiogenic effect of hydroxysafflor yellow A (HSYA): targeting HIF-1α and MMP9 in HMEC-1

**DOI:** 10.3389/fchem.2025.1713765

**Published:** 2026-01-06

**Authors:** Juanli Fu, Yingmei Dong, Zhifeng Yao, Jiaming Yu, He Wang, Yizheng Wang, Fan Lin

**Affiliations:** 1 College of Integrative Medicine, Fujian University of Traditional Chinese Medicine, Fuzhou, China; 2 Academy of Integrative Medicine, Fujian University of Traditional Chinese Medicine, Fuzhou, China

**Keywords:** HSYA, angiogenesis, network pharmacology, HIF-1α, MMP9

## Abstract

**Background:**

Angiogenesis is a fundamental physiological process mediating vascular network formation, represents a critical therapeutic target for ischemic diseases and tumor neovascularization. Xuefu Zhuyu decoction (XFZYD), a classical formula for promoting blood circulation and resolving stasis, demonstrates pro-angiogenic effect with safflower functioning as the sovereign herb. Hydroxysafflor yellow A (HSYA), the primary bioactive constituent of safflower, exerts potent angiogenesis modulation, defining its pharmacological significance.

**Methods:**

In this study, *in vitro* tubulogenesis assay and cytocompatibility analysis were employ on human microvascular endothelial cell (HMEC-1), followed by target prediction via network pharmacology and molecular docking; immunoblotting analysis was performed to experimentally validate the pro-angiogenic molecular mechanism of HSYA.

**Results:**

HSYA exerted concentration-dependent pro-angiogenic effects on HMEC-1 cells over 24 h without compromising cell viability (*p* > 0.05) across 0–200 μM. 121 potential targets of HSYA within the angiogenesis regulatory network were identified. Functional enrichment analysis revealed fluid shear stress, lipid metabolism, HIF-1, PI3K-Akt, and VEGF signal pathways as primary regulatory pathways. 8 hub targets derived from the protein-protein interaction (PPI) network were subjected to molecular docking. High-affinity interactions were observed for key angiogenesis regulators: MMP9 (−7.6 kcal·mol^−1^), and HIF-1α(−4.5 kcal·mol^−1^), which were functionally validated by immunoblotting analysis, preliminary demonstrating the mechanism of HSYA-mediated angiogenesis promotion.

**Conclusion:**

HSYA demonstrates significant pro-angiogenic activity on HMEC-1. Mechanistically, HSYA modulates multiple signaling pathways, with HIF-lα and MMP9 demonstrating regulatory significance. These findings suggest a molecular basis for HSYA’s therapeutic potential in ischemic vascular pathologies.

## 1 Introduction

Angiogenesis is the formation of new blood vessels from pre-existing vasculature. This process is essential in embryonic development and tissue repair, but also contributes to pathological conditions including ischemic pathology, tumor progression, and retinopathy (Li et al., 2025; Peng et al., 2025; Sobczynska-Rak et al., 2025). Consequently, pro-angiogenic therapies represent a major treatment strategy for ischemic diseases (Goncalves et al., 2021; Uccelli et al., 2019). Although gene therapies (e.g., VEGF delivery) are clinically implemented, their efficacy remains inconsistent (Apte et al., 2019). Therefore, developing stable therapeutic agents for angiogenesis-related pathologies constitutes a critical research priority (Luan et al., 2022).

XFZYD, a canonical formula in traditional Chinese medicine (TCM), is clinically established for treating ischemic cardiovascular and cerebrovascular diseases (Liang et al., 2020; Wang et al., 2024). Safflower (*Carthami Flos, Honghua*) serves as the sovereign herb in XFZYD formula, consisting of multiple bioactive components. To decipher the complex synergy within the formula, it is crucial to elucidate the contributions of its key active constituent. HSYA is a chalcone glycoside extracted from safflower, primarily mediating the herb’s therapeutic efficacy (Gao et al., 2018; Cheng et al., 2024). HSYA exhibits multifunctional pharmacological properties including anti-inflammatory, antioxidant, anti-coagulation and cardioprotective activities (Xue et al., 2021; Zhang et al., 2021).

The systematic molecular targets identification for therapeutic compounds construct a foundational step in modern drug discovery. This process is optimally facilitated through multi-database screening strategies, integrating chemogenomic repositories and target annotation platforms. Network pharmacology employs integrative approaches to generate multi-scale compound-target networks, providing a pharmacological profiling for mechanistic elucidation. These methods accelerate the transition from target-centric to network-centric drug development (Hopkins, 2008).

In this study, we examined the pro-angiogenic effects of HSYA on human microvascular endothelial cell (HMEC-1). The mechanisms of HSYA in regulating angiogenesis was investigated through integrated network pharmacology and molecular docking. Subsequent experimental validation via immunoblotting analysis was conducted to assess key signaling molecules. This study aims to establish a theoretical framework for elucidating the molecular basis underlying HSYA’s regulation of angiogenesis pathways.By bridging bioactive herb with modern pharmacology, we seek to develop novel therapeutic strategies against ischemic pathology.

## 2 Materials and methods

### 2.1 Cell culture and reagents

HMEC-1 cells were commercially obtaind from Immocell (Xiamen, China), and were cultured in MCDB131 basal medium (Sigma-Aldrich, St. Louis, United States) supplemented with 10% fetal bovine serum (Thermo Fisher Scientific, Waltham, United States), 1% penicillin-streptomycin (Thermo Fisher Scientific, Waltham, United States), 10 ng/mL recombinant human EGF (MedChemExpress, Monmouth Junction, United States), and 1 μg/mL hydrocortisone (SPExBIO, Houston, United States). Cells were maintained at 37 °C in a humidified 5% CO_2_ atmosphere with medium renewal every 48 h. All cellular experiments, including the CCK-8 assay, drug treatment, and angiogenesis assay, were performed using cells at passages 3–6 during their logarithmic growth phase after thawing.

HSYA (purity >98%, HeYuan LiJi Biotechnology, Shanghai, China) was dissolved in sterile ultrapure water to prepare a 50 mM stock solution. For treatment, HSYA was diluted in medium to a final concentration of 200 μM. Cells were exposed to HSYA for 24 h prior to functional assays.

Matrigel basement membrane matrix was purchased from Corning Inc(Corning,United States) and CCK-8 assay kit was from HeYuan LiJi Biotechnology (Shanghai, China). The antibodies against HIF-1α was purchased from Selleck Chemicals (Houston, United States). The antibodies against MMP9 was obtained from HUABIO(Zhejiang, China).

### 2.2 Cell viability assay

HMEC-1 cells at 80%–90% confluence were seeded into 96-well plates at 5 × 10^3^ cells/well and allowed to adhere for 24 h. The culture medium was then replaced with fresh medium containing HSYA at concentrations of 0 μM, 100 μM, or 200 μM. Each concentration was tested in triplicate wells. Following 24 h incubation, cells were washed with PBS and incubated for 4 h at 37 °C in 100 μL serum-free medium containing 10% CCK-8 reagent. Absorbance was measured at 450 nm using a microplate reader (Tecan, Zurich, Switzerland). Cell viability (%) was calculated as: Viability = [(OD treated group − OD blank group)/(OD control group − OD blank group)] × 100%.

### 2.3 *In vitro* angiogenesis assay

HMEC-1 cells (80%–90% confluence) underwent serum starvation for 6 h before grouping into 6-well plates (0 μM, 100 μM, 200 μM). Following the seeding of 4 × 10^5^ cells per well and culturing for 24 h, the cells were subjected to drug treatment for an additional 24 h. Matrigel obtained from Corning Inc. (Corning, United States) was thawed at 4 °C for 16 h, homogenized with chilled pipette tips and coated (50 μL/well) in 96-well plates.

Subsequently, the processed cells were collected and plated into Matrigel-polymerized (37 °C, 45 min, 5% CO_2_) 96 well plates in a quantity of 3 × 10^4^ cells/well. After 4 h, images of the nascent tube formation were captured using a phase-contrast microscope (Nikon Ts2, Wetzlar, Germany) at a ×200 magnification. The number of tubes in random fields per well was quantified by counting the minimal, contiguous tube structures formed by cells.

### 2.4 Identification of HSYA-angiogenic targets

Structural data of HSYA (2D SDF/SMILES) were downloaded from PubChem (CID: 6443665, accessed on 6 August 2024, https://pubchem.ncbi.nlm.nih.gov/). Related targets were predicted using PharmMapper (v2017, https://lilab-ecust.cn/pharmmapper/index.html) and SuperPred (accessed on 19 February 2025, http://prediction.charite.de/). To construct a comprehensive HSYA-target profile, all predictions from these two sources were consolidated into a union set. Angiogenesis-related genes were curated from GeneCards (v5.24, https://www.genecards.org/). In order to refine this disease gene set and enhance its specificity, a relevance score filter was applied, retaining only those genes with a score above the mean value of all retrieved angiogenesis-related genes. All target names from both the HSYA union set and the filtered angiogenesis set were standardized to official gene symbols for “*Homo sapiens*” via UniProtKB. (https://www.uniprot.org/). Core targets were identified by Venn diagram tool on the Microbiology platform (accessed on 19 February 2025, https://www.bioinformatics.com.cn/). Protein-protein interactions were analyzed by STRING (v12.0, https://cn.string-db.org/) with “*H. sapiens*” species in the confidence >0.9 and visualized in Cytoscape (v3.10.3, Institute of Systems Biology, United States).

### 2.5 GO and KEGG pathway enrichment analyses

Angiogenesis targets regulated by HSYA were analyzed in DAVID (v2024q4, https://davidbioinformatics.nih.gov/) for Gene Ontology (GO) and Kyoto Encyclopedia of Genes and Genomes (KEGG) pathway enrichment. Significant terms (*P* < 0.05, BH-corrected) were prioritized, selecting the top 10 GO terms and 20 KEGG pathways. Angiogenesis-associated pathways of HSYA were manually supplemented. A target-pathway network was constructed in Cytoscape (v3.10.3, Institute of Systems Biology, United States).

### 2.6 Molecular docking

Topological analyses of the 8 hub targets from HSYA-angiogenesis PPI network were employed. The 2D SDF structure of HSYA was converted to 3D coordinates using OpenBabel v3.1.1. Corresponding target proteins were retrieved from RCSB Protein Data Bank database (resolution ≤3.0 Å, https://www.rcsb.org/), preprocessed in PyMOL v2.5.4 through solvent molecule removal, polar hydrogen addition, and co-crystallized ligand elimination. Molecular docking was performed in AutoDockTools v1.5.6 and the docking poses were visualized using PyMOL v2.5.4. To validate the protocol, the native cognate ligand was re-docked, and its resulting binding energy and pose were compared with those of HSYA.

### 2.7 Immunoblotting analysis

HMEC-1 cells were seeded into 6-well plates at 4 × 10^5^ cells/well and cultured until reaching 80%–90% confluence. After 24 h of adherence, the cells were treated with 0 μM or 200 μM for an additional 24 h followed by Western blotting analysis of target proteins (HIF-1α and MMP9). Total protein was extracted and quantified via bicinchoninic acid assay. Sodium dodecyl sulfate polyacrylamide gel electrophoresis (SDS-PAGE) separation with 10% Bis-Tris gel were performed and transfered with polyvinylidene fluoride (PVDF) membrane. Membranes were blocked with 5% sterile skim milk and probed with primary antibodies (4 °C, 16 h). After washing, membranes were incubated with secondary antibodies for 1 h at room temperature and bands were captured on a using an enhanced ehemiluminescence (ECL) detection system. Primary antibodies were obtained from the following sources: anti- HIF-1α and anti-MMP9 were from HuaAn Biotechnology (Catalog #: HA721997 and ET1704-69, respectively; both used at 1:1000); anti-β-actin, used as a loading control, was from Abways (Catalog #: AB0035; used at 1:6000).

### 2.8 Statistical analysis

Statistical analyses were performed using SPSS 25.0. All data were confirmed to meet assumptions of normality (Shapiro-Wilk test) and homogeneity of variance (Levene’s test) prior to parametric testing. Independent-sample T-tests were used for comparisons between two groups, and one-way ANOVA for comparisons among multiple groups. A *p*-value of less than 0.05 was considered statistically significant. Results were plotted using GraphPad Prism 9.0.

## 3 Results

### 3.1 HSYA preserved cellular vitality in endothelial cells and exhibited pro-angiogenic activaty

**FIGURE 1:**
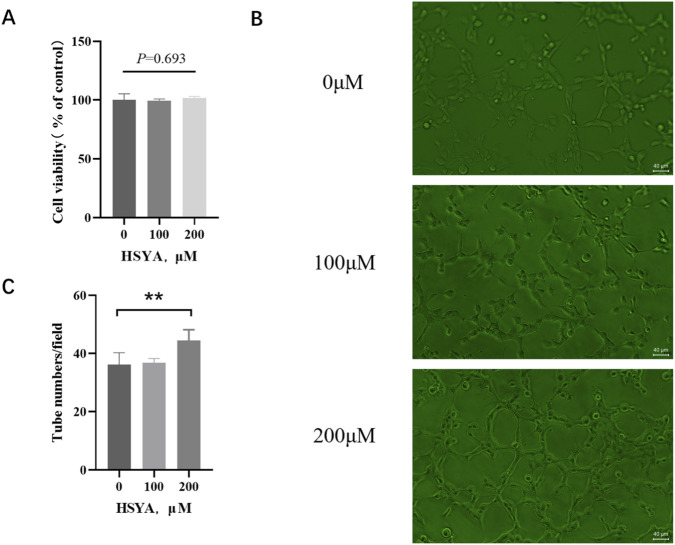
HSYA maintained the cellular viability of endothelial cells and promoted the tube formation of HMEC-1. **(A)** Effects of different concentrations (0 μM, 100 μM, 200 μM) of HSYA on HMEC-1 cell viability (n = 3 independent experiments), Data are mean ± SD; ns, not significant (one-way ANOVA, p = 0.693). **(B)** Effects of different concentrations (0 μM, 100 μM, 200 μM) of HSYA on HMEC-1 tube formation. Scale bar, 40 μm. **(C)** Quantification of tube numbers per field (n = 3 independent experiments) Data are mean ± SD; **p < 0.01 vs. 0 μM group (one-way ANOVA with LSD *post hoc* test).

HSYA maintained ≥99% cellular vitality in human microvascular endothelial cells across 0–200 μM exposures for 24 h. As shown in Figure 1A, viability remained at 99.32% ± 1.68% (100 μM), and 101.64% ± 1.49% (200 μM) versus untreated controls, confirming uncompromised bioenergetic function within pharmacologically active ranges essential for angiogenic research. Following 24 h exposure to HSYA, 200 μM HSYA treatments markedly enhanced tube formation capacity (*p* < 0.01) on growth factor-reduced Matrigel (Figures 1B,C). HSYA demonstrated dose-dependent pro-angiogenic enhancement *in vitro* without affecting cellular proliferation, indicating that its functional mechanism modulates cellular behavior rather than directly inducing mitogenic activity.

### 3.2 Hub genes mediating HSYA’s angiogenesis modulation were prioritized

**FIGURE 2:**
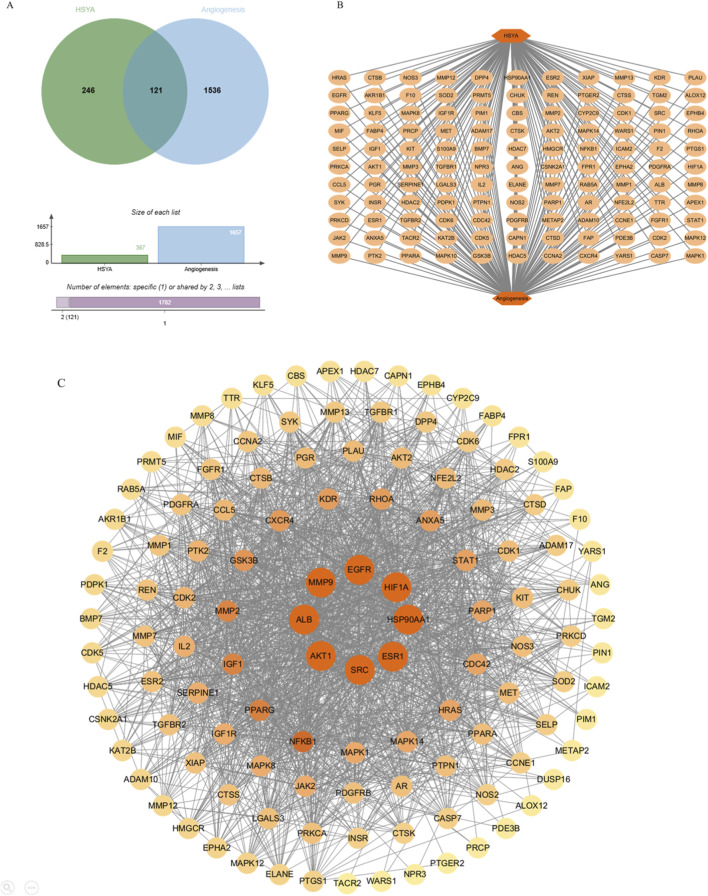
Network of HSYA-angiogenesis targets. **(A)** Venn diagram for overlapping genes of HSYA-angiogenesis. **(B)** Overlapping targets of HSYA-angiogenesis network. **(C)** PPI network of targets uderlying HSYA’s pro-angiogenic activity. PPI = protein-protein interaction.

Bioinformatic analysis integrating PharmMapper, and SuperPred identified 367 potential targets of HSYA. Cross-referencing with GeneCards angiogenesis-associated targets, 121 targets of HSYA-angiogenesis network was revealed (Figures 2A,B). To identify the hub targets, a PPI network was constructed based on topological analysis, which indicating complex multi-target coordination. 8 hub genes (*AKT1*, *ALB*, *MMP9*, *HIF1A*, *HSP90AA1*, *SRC*, *EGFR*, *ESR1*) were identified with highest centrality metrics as presented in Figure 2C (degree: the number of direct connections a node has within a network; betweenness: Quantifies how often a node acts as a bridge along the shortest path between two other nodes; proximity: the reciprocal of the sum of the shortest path lengths from a node to all other reachable nodes).

### 3.3 Enrichment analysis displayed the polypharmacological profile

Functional enrichment analysis of HSYA-angiogenesis hub targets identified 106 significant GO terms (*p <* 0.05) and 47 KEGG pathways, with the top deciles revealing key mechanistic insights.

**FIGURE 3:**
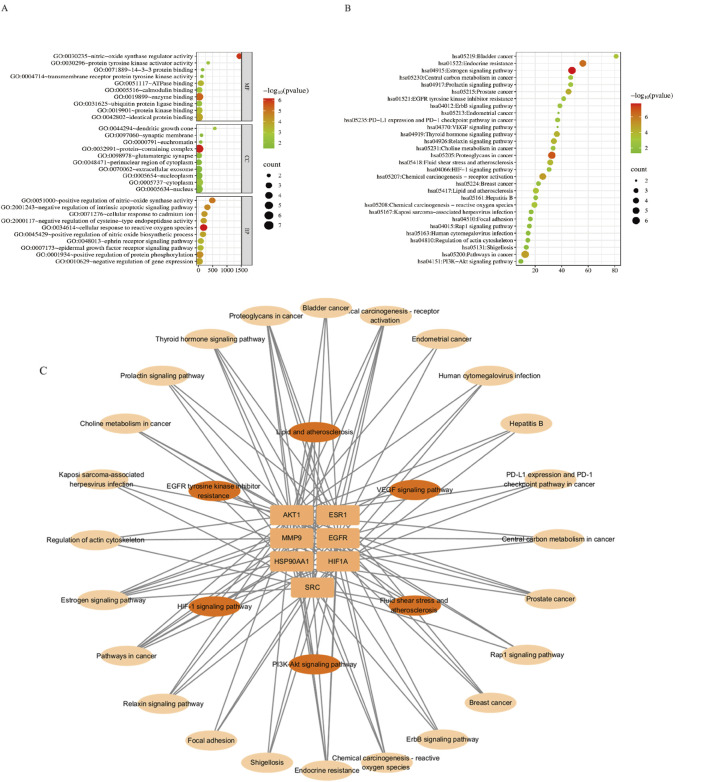
GO and KEGG enrichment analysis. **(A)** GO analysis of HSYA-angiogenesis targets. MF = molecular function, CC = cellular component, BP = biological process. **(B)** KEGG pathway plot. **(C)** Target-pathway network.

GO analysis revealed significant enrichment in key biological processes such as cellular response to reactive oxygen species (GO:0034614, adjusted *P*-value = 7.06E-07, Fold Enrichmen = 179.7777778, Count = 4), positive regulation of nitric-oxide synthase activity (GO:0051000, adjusted *P*-value = 1.17E-05, Fold Enrichment = 485.4, Count = 3), positive regulation of nitric oxide biosynthetic process (GO:0045429, adjusted *P*-value = 1.14E-04, Fold Enrichment = 158.2826087, Count = 3), epidermal growth factor receptor signaling pathway (GO:0007173, adjusted *P*-value = 4.90E-04, Fold Enrichment = 76.64210526, Count = 3), with cellular localization to cytoplasm (GO:0005737, adjusted *P*-value = 0.002822662, Fold Enrichment = 3.071640904, Count = 7), nucleus (GO:0005634, adjusted *P*-value = 0.027546997, Fold Enrichment = 2.525171065, Count = 6), and nucleoplasm (GO:0005654, adjusted *P*-value = 0.02975706, Fold Enrichment = 3.2290625, Count = 5). Molecular functions predominantly involved nitric-oxide synthase regulator activity (GO: 0030235, adjusted *P*-value = 1.14E-06, Fold Enrichment = 1440.6, Count = 3) and protein binding (Figure 3A). KEGG pathway analysis highlighted fluid shear stress, lipid metabolism, HIF-1 (hsa04066, adjusted *P*-value = 0.003021664, Fold Enrichment = 30.49885321, Count = 3), PI3K-Akt (hsa04151, adjusted *P*-value = 0.030473867, Fold Enrichment = 9.183356354, Count = 3), and VEGF signaling pathway (hsa04370, adjusted *P*-value = 0.046441529, Fold Enrichment = 36.9375, Count = 2) (Figure 3B). Target-pathway network visualization confirmed HSYA’s diverse effects in which a individual gene participating in ≥7 pathways and each pathway integrated ≥2 targets, establishing a polypharmacological framework for vascular modulation (Figure 3C).

### 3.4 HSYA demonstrated high-affinity binding to the angiogenic regulators

**FIGURE 4:**
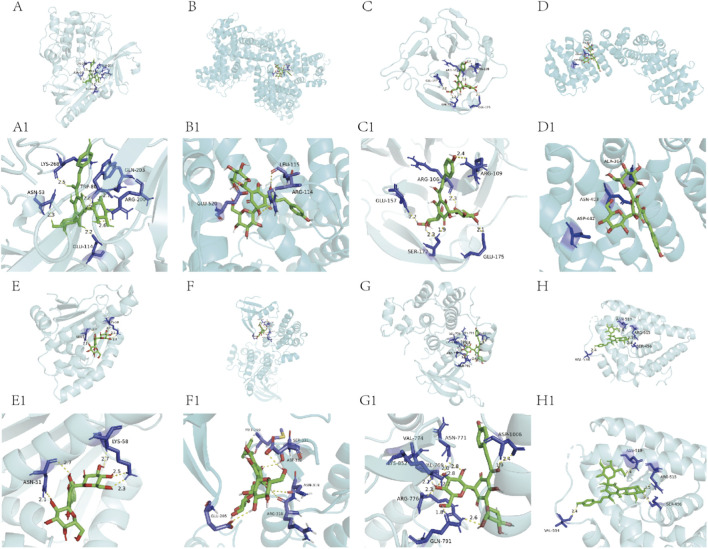
Molecular docking maps of the hub targets with HSYA. **(A, A1)** AKT-HSYA. **(B, B1)** ALB-HSYA. **(C, C1)** MMP9-HSYA. **(D, D1)** HIF-1α-HSYA. **(E, E1)** HSP90α-HSYA. **(F, F1)** Src-HSYA. **(G, G1)** EGFR-HSYA. **(H, H1)** ERα-HSYA. The yellow dotted lines are the hydrogen bonds.

Molecular docking analysis revealed potent interactions between HSYA and the protein products of eight hub genes (*AKT1*, *ALB*, *MMP9*, *HIF1A*, *HSP90AA1*, *SRC*, *EGFR*, *ESR1*). HSYA demonstrated substantial binding affinities to the hub molecular targets, with computationally derived energy values indicating biological relevance. Notably, two pro-angiogenic regulators—MMP9 and HIF-1α showed exceptional binding ability. The binding affinity of HSYA to MMP-9 (ΔG = −7.6 kcal/mol) and HIF-1α (ΔG = −6.5 kcal/mol)was comparable to or exceeded that of natural ligands (−7.5 kcal/mol and −3.1 kcal/mol, respectively), suggesting the significance of further investigation. These findings highlight HSYA’s polypharmacological potential by targeting core vasoregulatory proteins involved in angiogenesis (Figure 4; Table 1).

**TABLE 1:** Binding affinities of HSYA and native ligands to hub targets.

Sequence number	Gene name	Protein name	PDB-ID	Binding energy with natural ligand (kcal/mol)	Binding energy with HSYA (kcal/mol)
1	AKT1	AKT1	8uw9	−12.9	−9.9
2	ALB	ALB	4l9k	−8.1	−7.3
3	MMP9	MMP-9	1itv	−7.5	−7.6
4	HIF1A	HIF-1α	8he3	−3.1	−6.5
5	HSP90AA1	HSP90α1	3o0i	−10.8	−8.5
6	SRC	Src	3d7t	−11.1	−8.7
7	EGFR	EGFR	5d41	−8.6	−7.7
8	ESR1	ESR1	6pdj	−9.9	−7.2

### 3.5 Experimental validation of the pharmacological mechanism underlying HSYA-mediated angiogenesis

**FIGURE 5:**
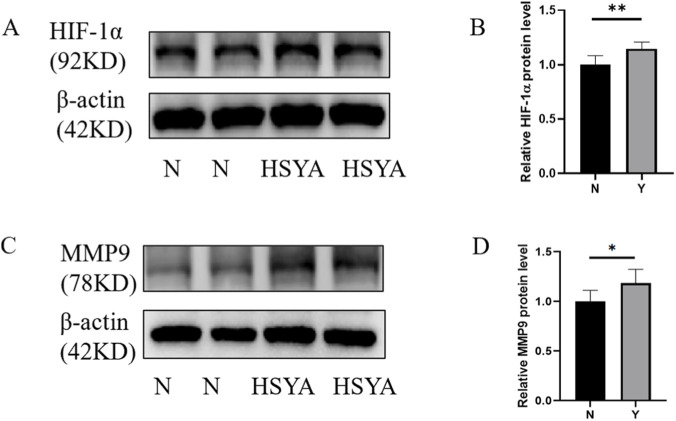
Western blotting of key regulators followed by HSYA treatment. **(A)** Western blotting of HIF-1α protein expression under HSYA treatment (200 μM). **(B)** Quantification of relative protein level (n = 3 independent experiments). Data are mean ± SD; (**p < 0.01). **(C)** Western blotting of MMP9 protein expression under HSYA treatment (200 μM). **(D)** Quantification of relative protein level (n = 3 independent experiments). Data are mean ± SD; *p < 0.05).

Western blotting quantification confirmed significant elevation in protein expression of key angiogenesis regulators following HSYA treatment: HIF-1α (1.15 ± 0.62, **p < 0.01), MMP9 (1.18 ± 0.14, *p < 0.05) demonstrated dose-dependent upregulation versus vehicle controls (Figure 5). These results preliminary demonstrate the mechanism of HSYA driven angiogenesis promotion via multi-target moderation, with HIF-1α and MMP9 emerging as potential key regulators of this biological process.

## 4 Discussion

HSYA, the predominant water-soluble bioactive fraction of safflower, belongs to the quinone-type chalcone family. This compound is characterized by an A-ring oxidized to a quinone or quinone-like moiety and exists primarily in the form of a carbon glycoside. Quinone-type chalcones, including HSYA, are considered the primary pharmacophore responsible for the blood-activating and stasis-resolving effects of safflower (Zhao et al., 2020). HSYA demonstrates regulation of angiogenesis in previous findings: it potentiates therapeutic neovascularization in myocardial ischemia via Ang-1/Tie2 signaling and enhances tubulogenesis in human umbilical vein endothelial cell (HUVEC) through VEGF-A/MMP9 upregulation (Chen et al., 2016; Ruan et al., 2021; Yang et al., 2015; Yu et al., 2019; Zou et al., 2018). Our study confirmed the promotion of angiogenesis activity of HSYA, which consistent with previous research. Through integrated network pharmacology, we identified *AKT1*, *ALB*, *MMP9*, *HIF1A*, *HSP90AA1*, *SRC*, *EGFR*, and *ESR1* as hub genes of HSYA-angiogenesis network, and a novel HIF-1α/MMP-9-associated mechanism was revealed, broadening the known signaling paradigm.

As a process essential for tissue development and repair, angiogenesis is initiated by matrix metalloproteinase-mediated degradation of the basement membrane, facilitating endothelial cell invasion into the extracellular matrix (ECM). This is followed by coordinated cellular proliferation, migration, and tubular morphogenesis, finally form the functional vasculature. In our findings, CCK-8 analysis and *in vitro* angiogenesis evaluation confirmed HSYA’s pro-angiogenic properties without affecting proliferation, consistent with prior reports across various cellulars and tissues (Guan et al., 2013; Han et al., 2016; Mao et al., 2025; Yu et al., 2024). This indicates that HSYA enhances angiogenesis not through mitogenic effects, but likely via modulation of cellular motility—possibly through cytoskeletal reorganization or adhesion dynamics.

While presented experimental evidence served as the decisive criterion for target selection. The absence of proliferative effects for HSYA demonstrated by the CCK-8 assay excluded AKT1, EGFR, and SRC as potential targets in this study, since their established roles as pro-proliferative signaling mediators.Similarly, inhibition of HSP90AA1 would inevitably induce anti-proliferative effects through client protein degradation, which conflicts with our experimental observations. ESR1 was excluded from experimental validation due to its profound dependence on hormonal regulation, a variable not controlled in our model system. Currently, no evidence implicates a significant contribution of differentiation pathways in the observed pro-angiogenic effects. ALB is primarily involved in nutritional and osmotic regulation, lacks direct association with angiogenic signaling pathways, and was therefore excluded from this study. Consequently,to prioritize the core angiogenic mechanism, we focused exclusively on MMP and HIF-1α, omitting proliferation markers and differentiation regulators in the current study. The potential regulatory roles of these excluded markers reserved for future exploration.

Matrix metalloproteinase 9 (MMP9) is critically involved in the degradation of ECM components such as collagen and gelatin, promoting tissue remodeling and inflammatory responses (Wang et al., 2022). Its enzymatic activity facilitates endothelial cell migration and the formation of tubular structures by breaking down the basement membrane (Li and Xu, 2025). Moreover, MMP9 contributes to the release of sequestered pro-angiogenic factors, such as vascular endothelial growth factor (VEGF), thereby promoting angiogenesis (Chen et al., 2021).

Hypoxia-inducible factor-1α (HIF-1α), a key transcriptional regulator of hypoxia adaptation, maintains oxygen homeostasis by governing downstream genes (Fakhri et al., 2024; Ge et al., 2023). It demonstrates cardioprotection against myocardial ischemia/re-perfusion injury (MI/RI) by suppressing oxidative stress, autophagy, inflammation, and apoptosis, while also modulating ferroptosis. As a hypoxia-responsive transcription factor, HIF-1α promotes endothelial cell proliferation and angiogenesis by inducing the expression of VEGF, PDGF, and other pro-angiogenic factors under hypoxia conditions (Yang et al., 2015). Our findings demonstrate HSYA’s pro-angiogenic effects through network pharmacology, uncovering previously unrecognized regulatory mechanisms involving the HIF-1α/MMP-9 axis. This polypharmacological action suggests HSYA’s therapeutic potential via coordinated hypoxic response and extracellular matrix regulation, suggesting the disease-specific mechanisms may provide clinical benefits.

This study primarily provides mechanistic insights into HSYA-mediated angiogenesis promotion effects, though it is limited by the reliance on *in vitro* models and absence of further mechanistic investigation. Future investigations would incorporate *in vivo* studies, confirmation of molecular docking results and functional characterization through protein knockout/restoration approaches to substantiate the mechanistic foundations and advance clinical application potential.

In TCM, safflower functions as a *Huoxue Huayu* (blood-activating and stasis-resolving) herb. Within the XFZYD formulation, safflower serves as sovereign herb, providing the primary therapeutic activity that directs the pharmacological actions of the entire prescription (Dai et al., 2025). Safflower acts as both a therapeutic spearhead disrupting pathological stasis and a regenerative catalyst promoting vascular reconstruction. This dual functionality—simultaneously eliminating obstructions and fostering angiogenesis—creates critical therapeutic windows, thereby embodying XFZYD’s foundational principle of *Qu Yu Sheng Xin* (removing stasis to generate renewal). In our study, HSYA induced *Sheng Xin* (pro-angiogenic effects) in microvascular endothelial cells, evidenced by accelerated tube formation and key molecular upregulation. This mechanistic insight further validates safflower’s role in synergizing XFZYD’s multicomponent activity.

Although HSYA has therapeutic potential in angiogenesis, clinical translation is hindered by low oral bioavailability. Recent studies show that formulating HSYA into water-in-oil nanoemulsions enhance oral bioavailability and prolong duration, offering possible clinical solutions (Lv et al., 2012; Zhao et al., 2020). The interpenetrating polymer network (IPN) hydrogel enables co-delivery of deferoxamine and HSYA, demonstrating capabilities in promoting angiogenesis and suppressing inflammation. This approach significantly accelerates diabetic wound healing, representing a promising nonoral therapeutic alternative (Gao et al., 2018).

Furthermore, previous studies have demonstrated the efficacy of a natural deep eutectic solvent (DES) system for enhancing the bioavailability of HSYA. A system of L-proline-acetamide (L-Pro-Am) increase the oral bioavailability (Fr, to water extract) of HSYA to183.5%. A deep eutectic solvent composed of glucose and choline chloride containing 10% (v/v) water enhance the relative oral bioavailability of HSYA to 326.08%, demonstrating the potential of DES as an effective delivery carrier for HSYA (Gao et al., 2022; Tong et al., 2021). Future investigations will optimize the choline chloride-based deep eutectic solvent to enhance the oral bioavailability of HSYA. We propose to establish an biocompatible, non-toxic drug delivery system to maximize clinical utility of safflower-derived bioactive compounds.

## 5 Conclusion

The natural compound HSYA exhibited potent pro-angiogenic activity in HMEC-1. Initial mechanistic investigations revealed its mechanism involve the modulation of HIF-1α and MMP9 activation pathways. These findings contribute to the understanding of natural product-based angiogenesis regulation and suggest potential therapeutic applications for angiogenesis-impaired vascular pathologies.

## Data Availability

The datasets presented in this study can be found in online repositories. The names of the repository/repositories and accession number(s) can be found in the article/Supplementary Material.
