# Supplementary figures and images for: Insights into the pro-angiogenic effect of hydroxysafflor yellow A (HSYA): targeting HIF-1α and MMP9 in HMEC-1
Source: Front Chem. 2026 Jan 6;13:1713765. doi: 10.3389/fchem.2025.1713765 (PMC12816176; doi:10.3389/fchem.2025.1713765)

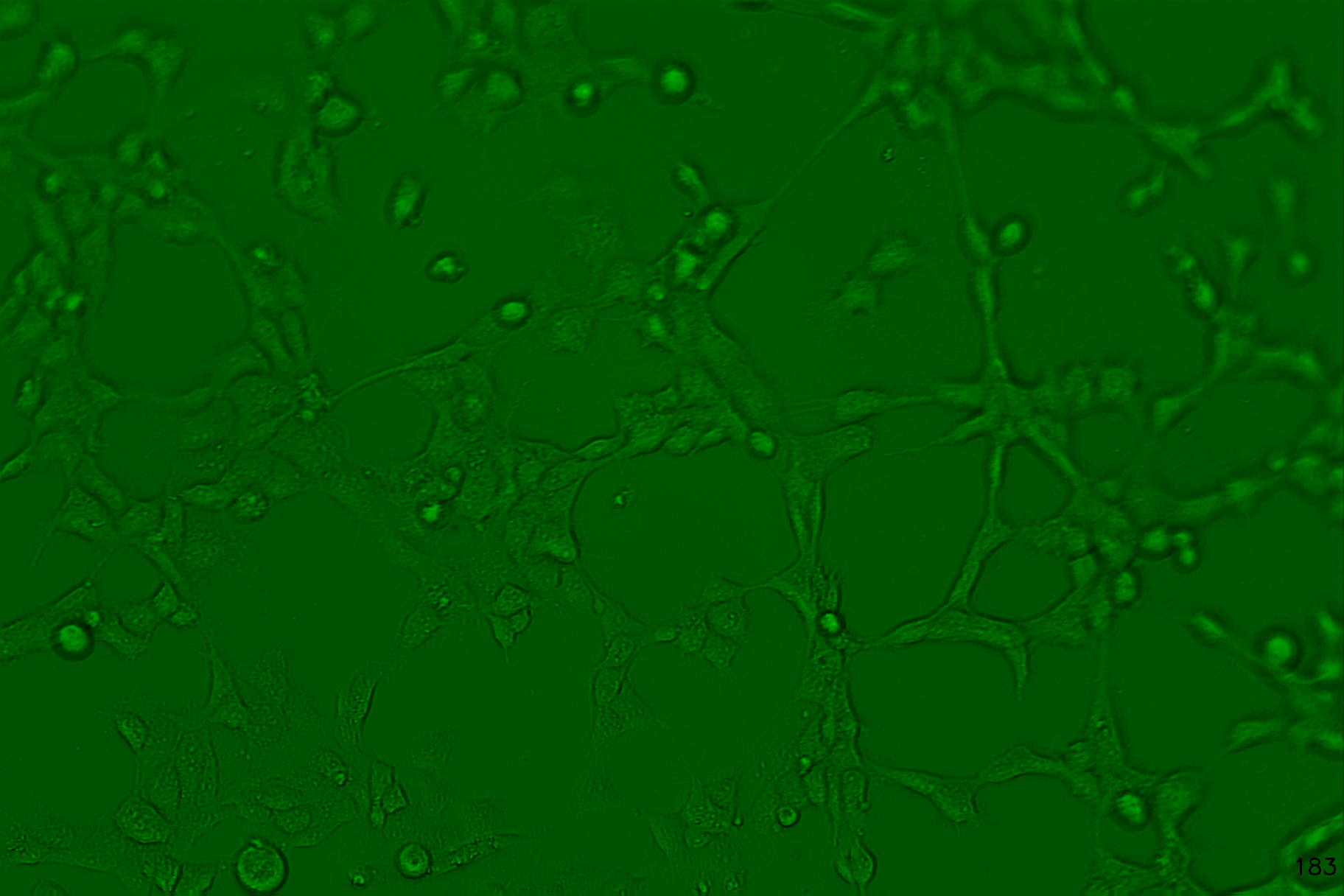

Supplement: Supplementary file 1 [file DataSheet3.zip › Figure1-original/Figure1B/0μM.tif]

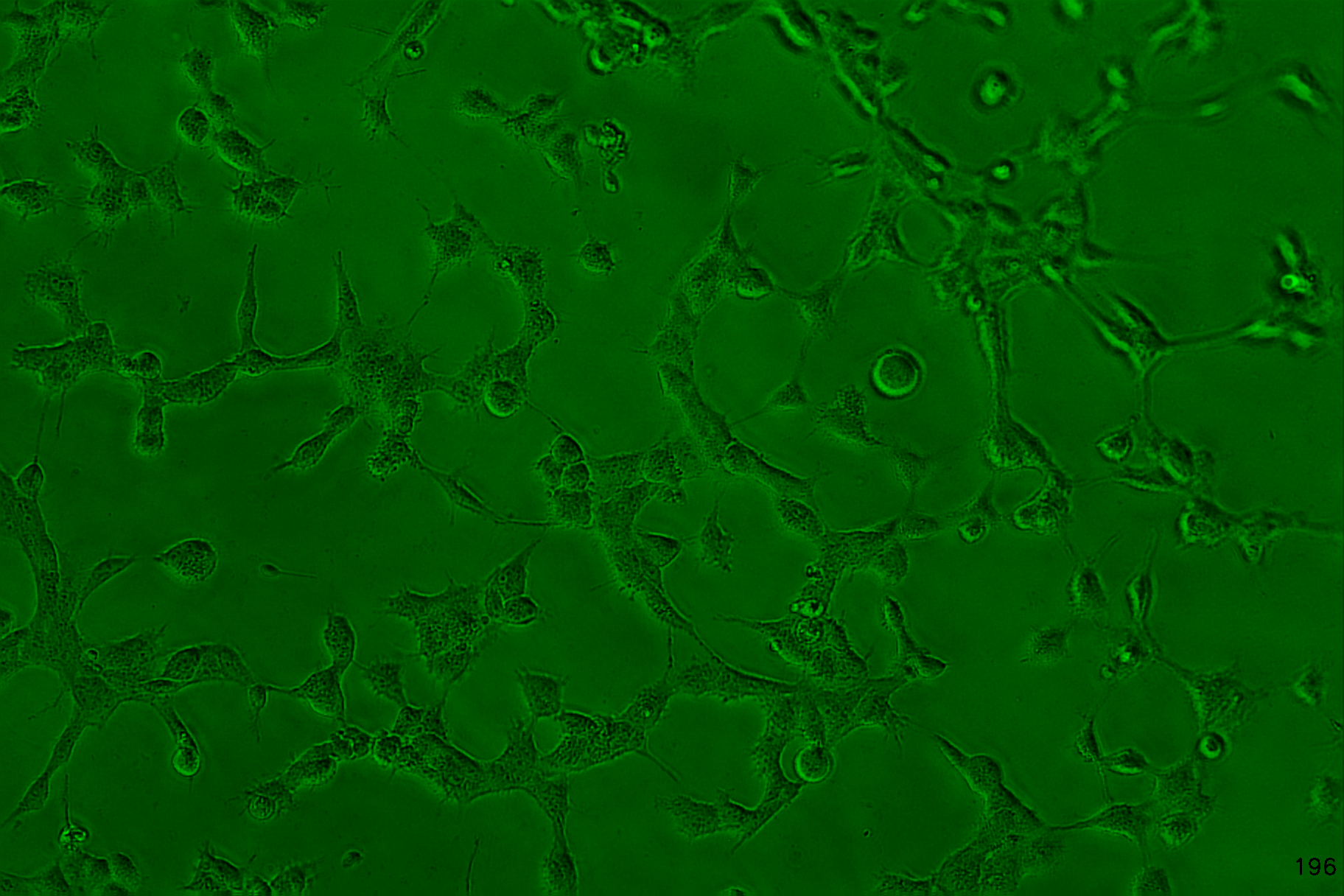

Supplement: Supplementary file 1 [file DataSheet3.zip › Figure1-original/Figure1B/100μM.tif]

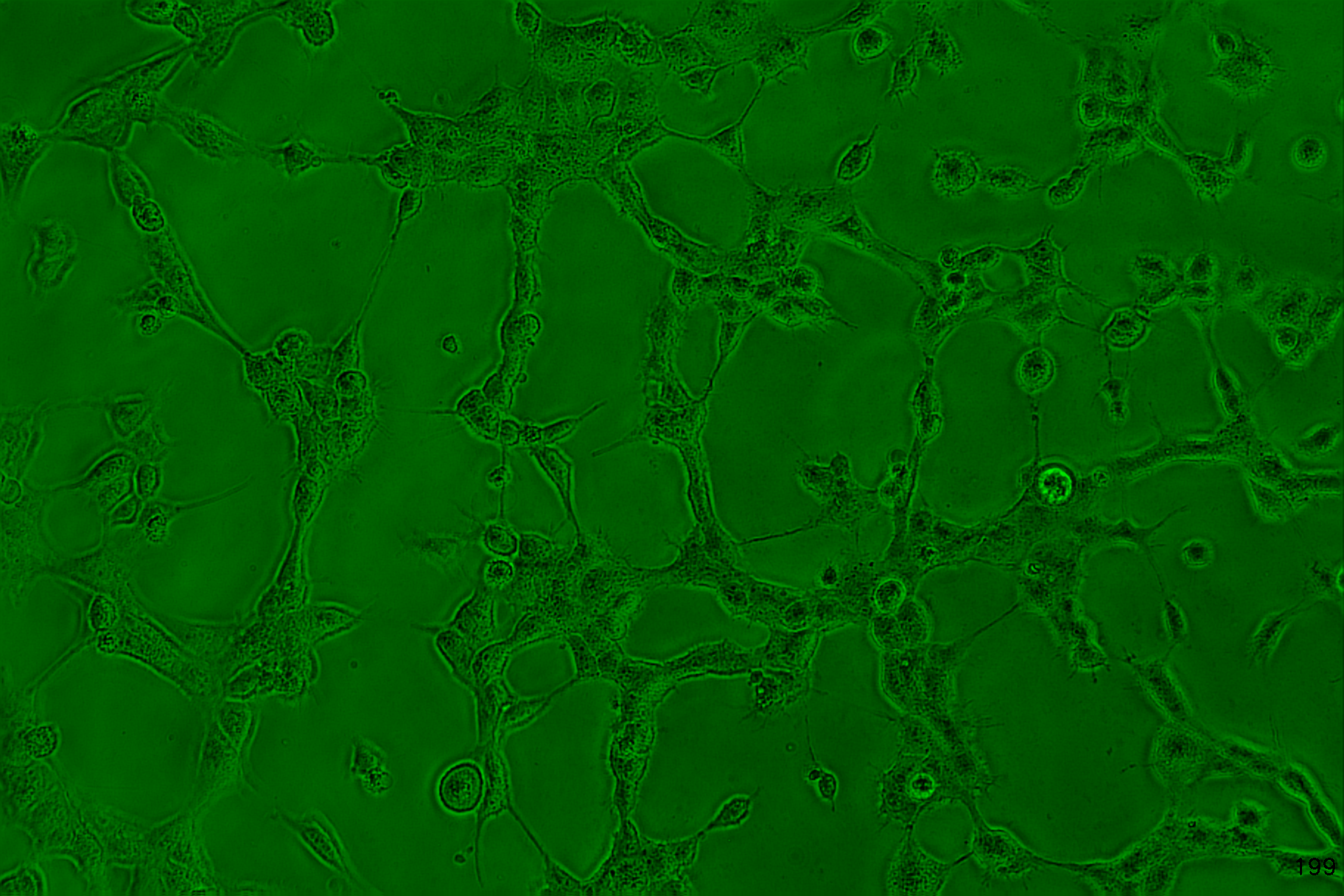

Supplement: Supplementary file 1 [file DataSheet3.zip › Figure1-original/Figure1B/200μM.tif]

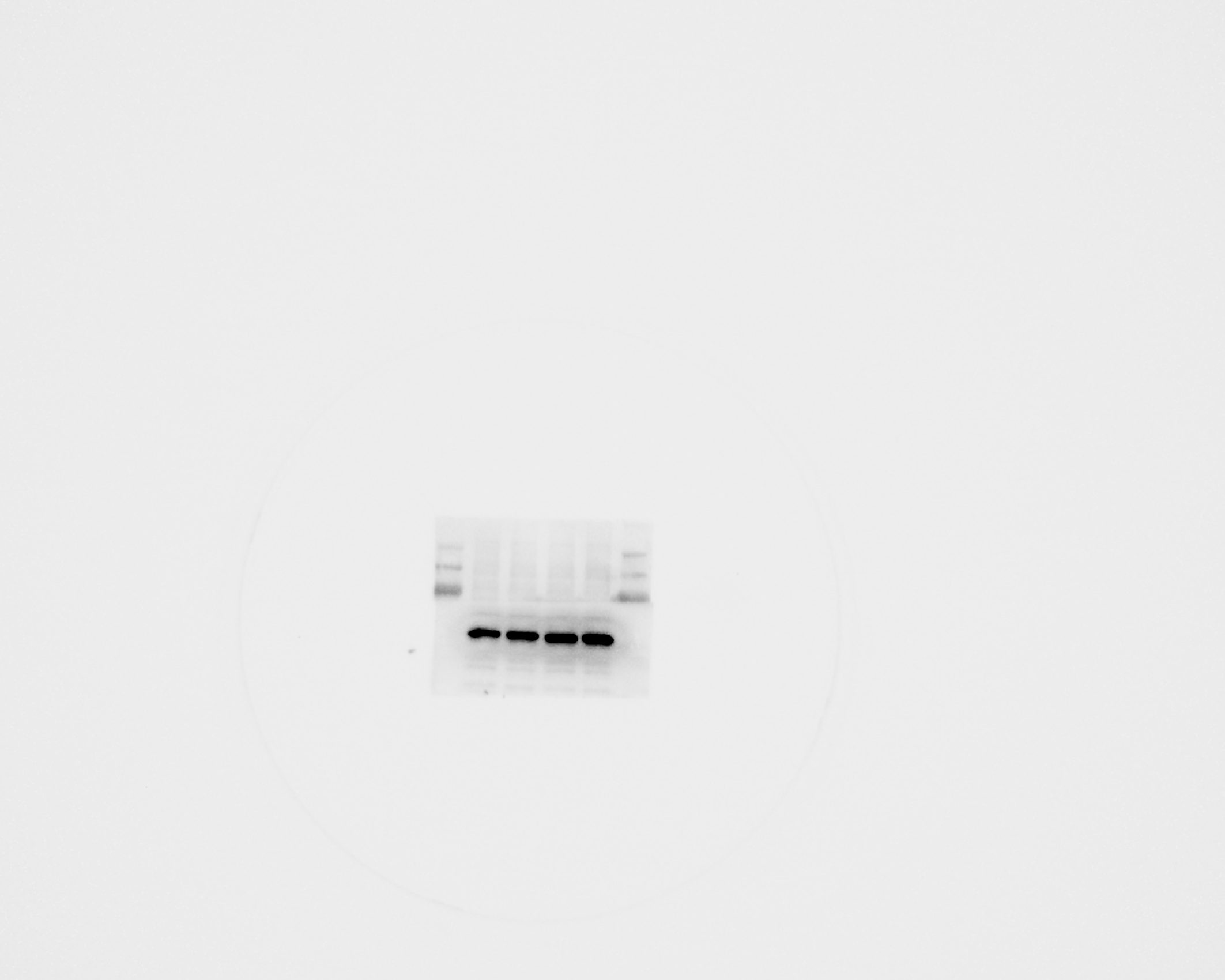

Supplement: Supplementary file 2 [file DataSheet8.zip › 1MMP9/1MMP9&beta(Chemiluminescence).tif]

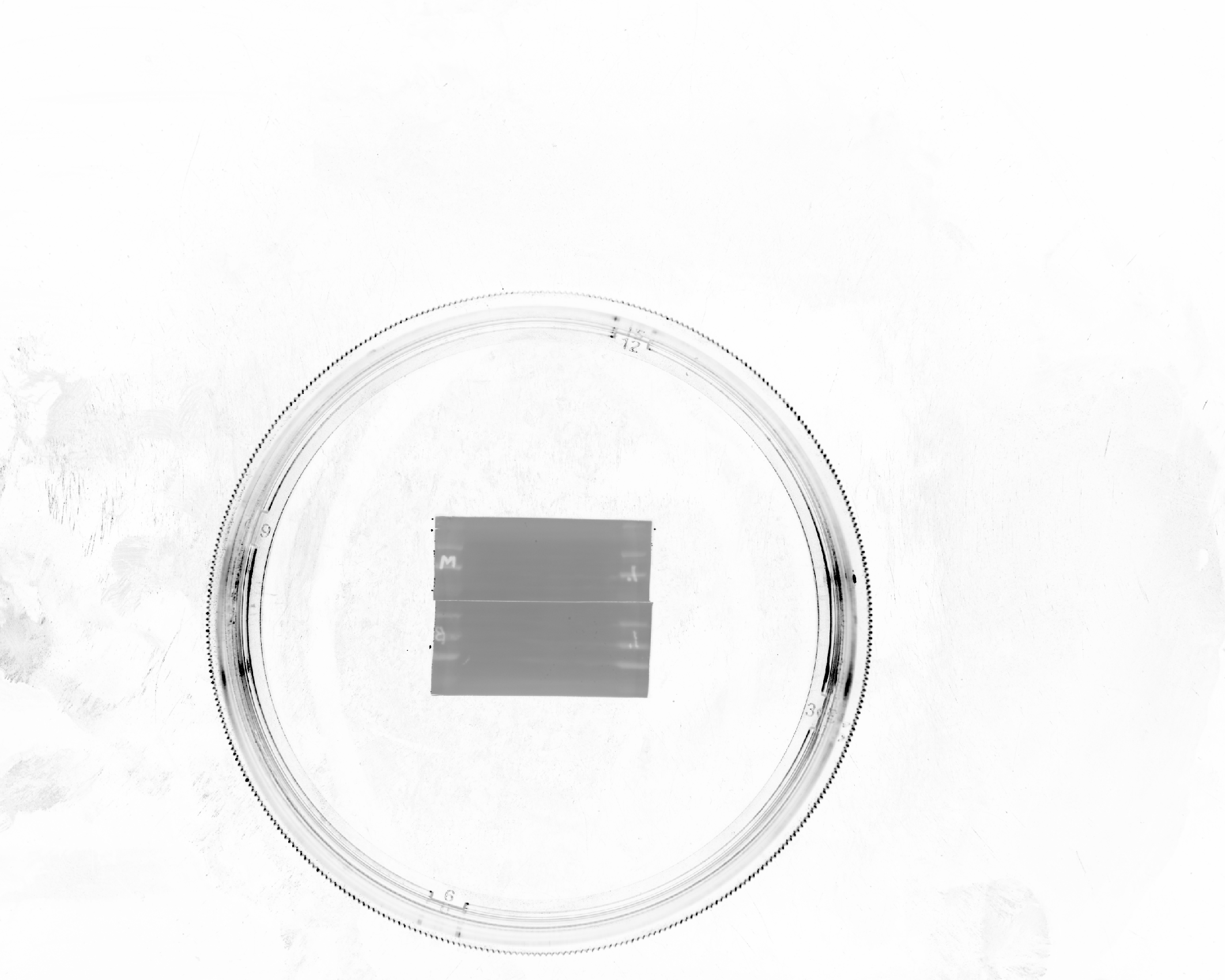

Supplement: Supplementary file 2 [file DataSheet8.zip › 1MMP9/1MMP9&beta-(Colorimetric).tif]

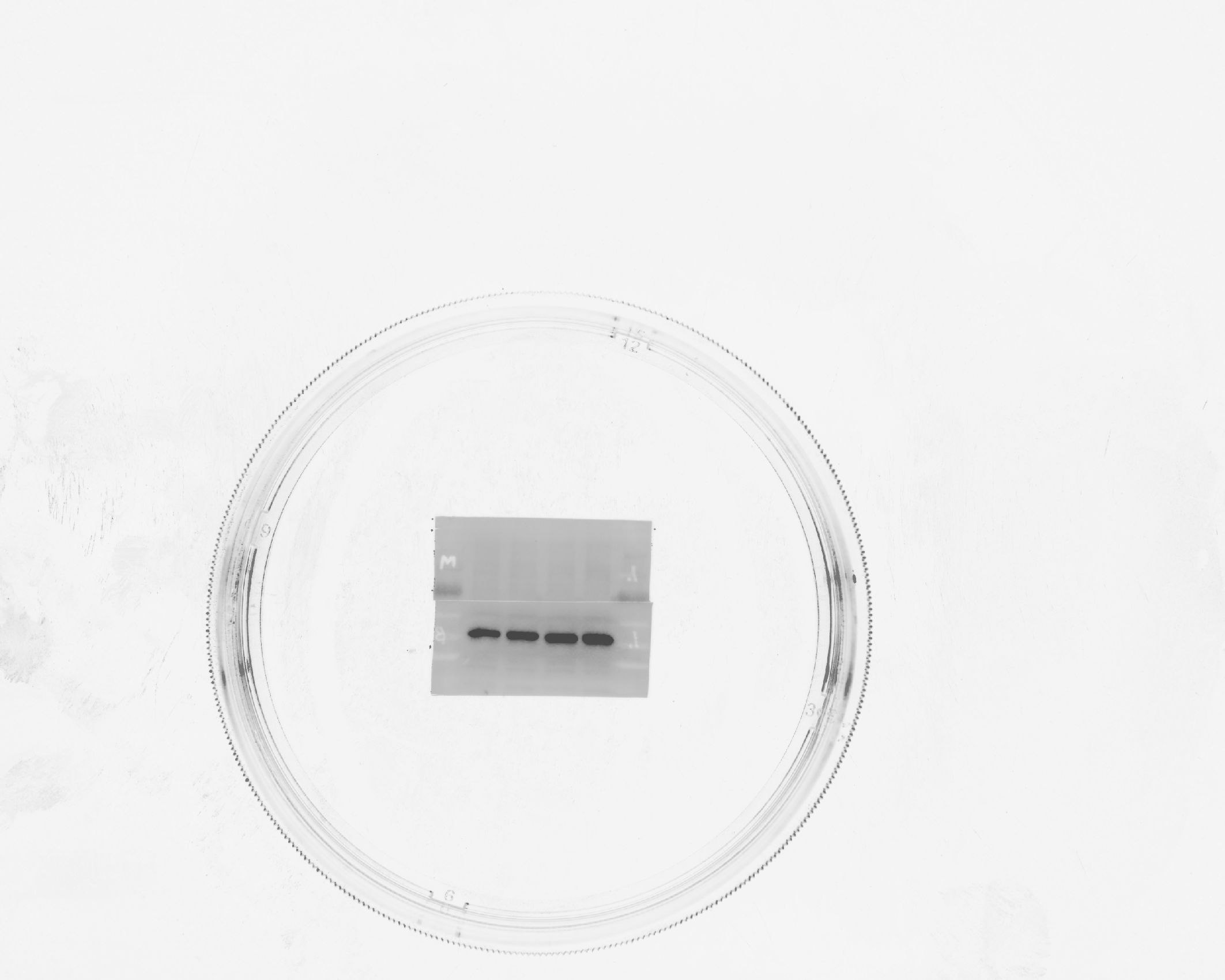

Supplement: Supplementary file 2 [file DataSheet8.zip › 1MMP9/1MMP9&beta-(复合).tif]

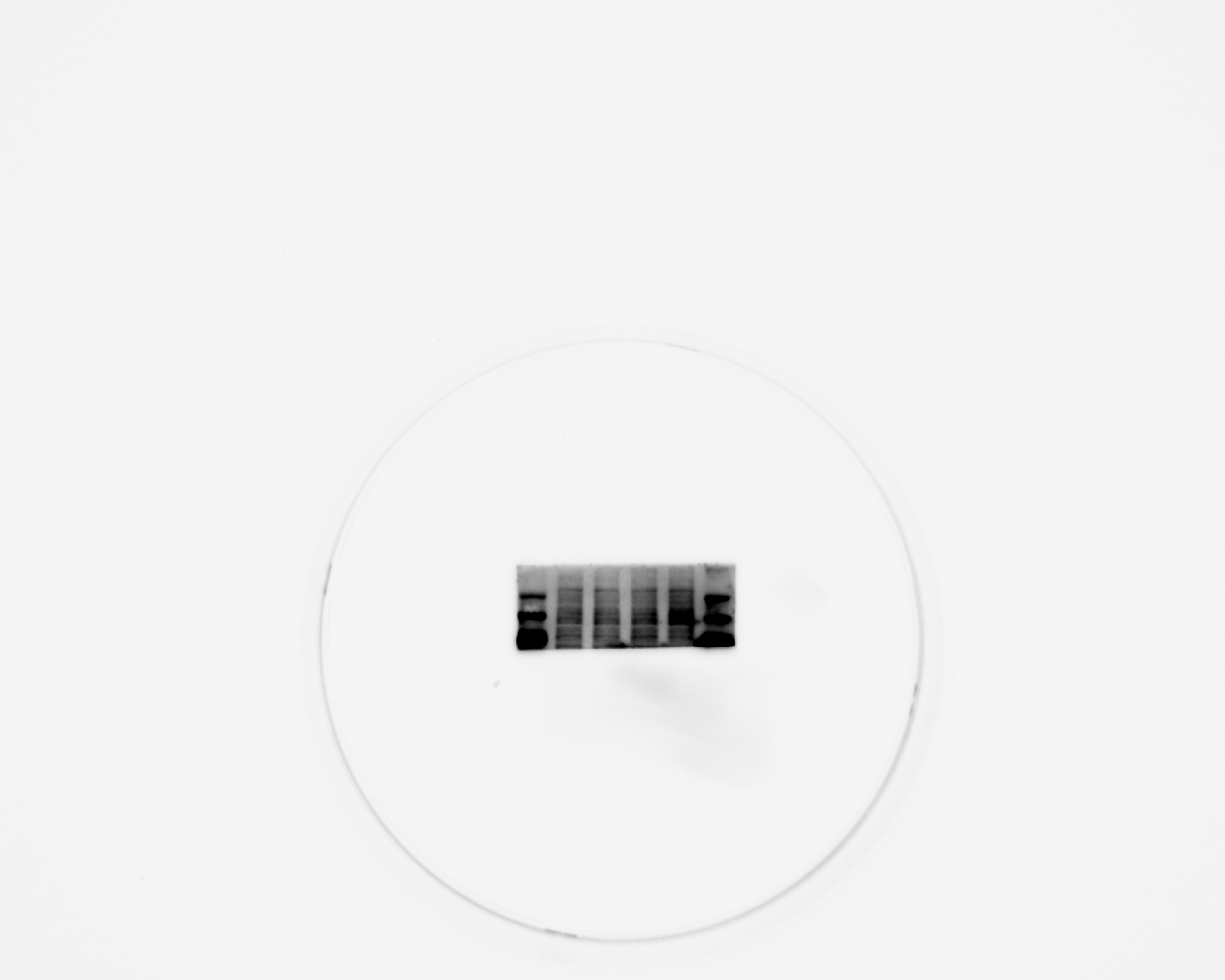

Supplement: Supplementary file 2 [file DataSheet8.zip › 1MMP9/1MMP9(Chemiluminescence).tif]

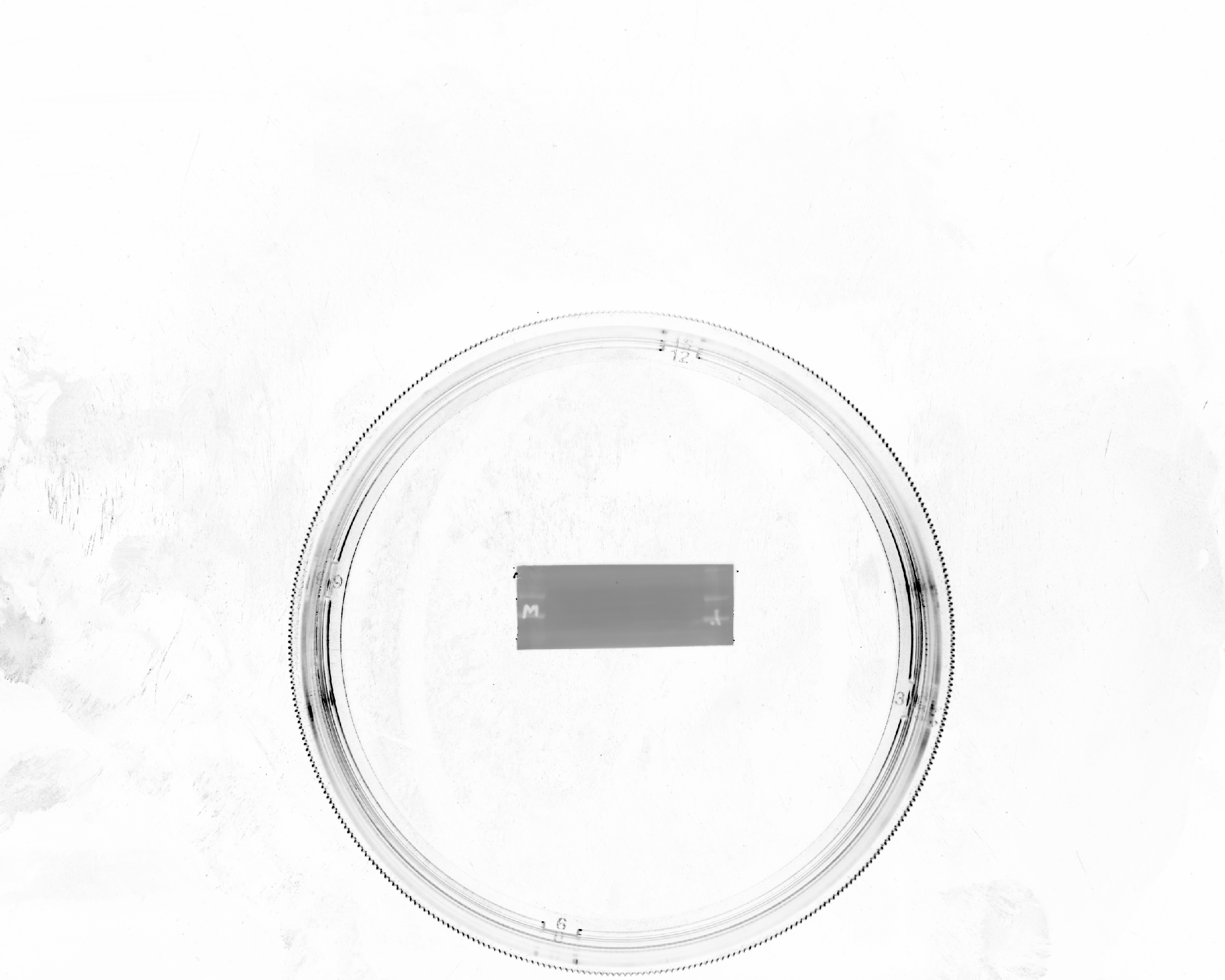

Supplement: Supplementary file 2 [file DataSheet8.zip › 1MMP9/1MMP9-(Colorimetric).tif]

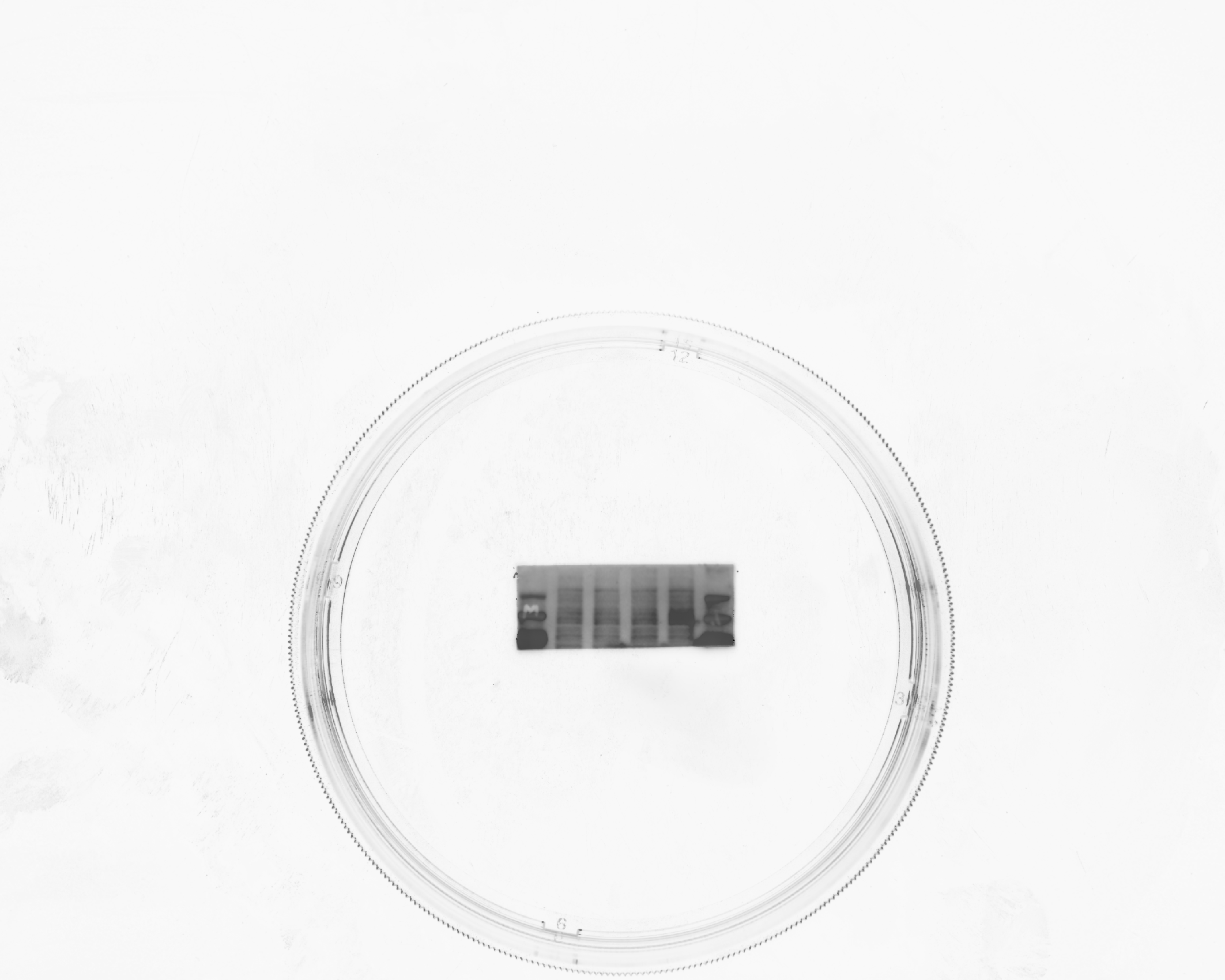

Supplement: Supplementary file 2 [file DataSheet8.zip › 1MMP9/1MMP9-(复合).tif]

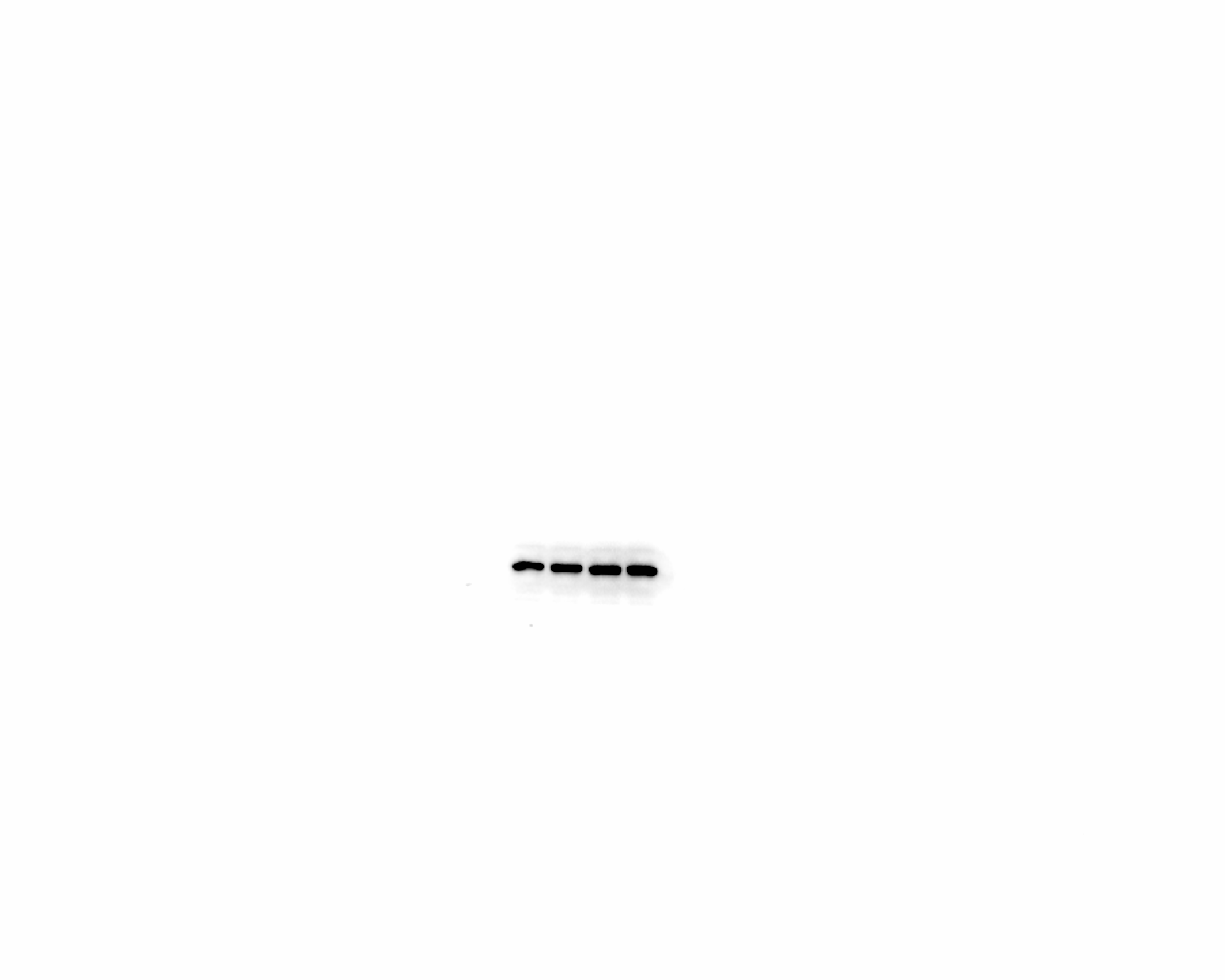

Supplement: Supplementary file 2 [file DataSheet8.zip › 1MMP9/1MMP9-beta(Chemiluminescence).tif]

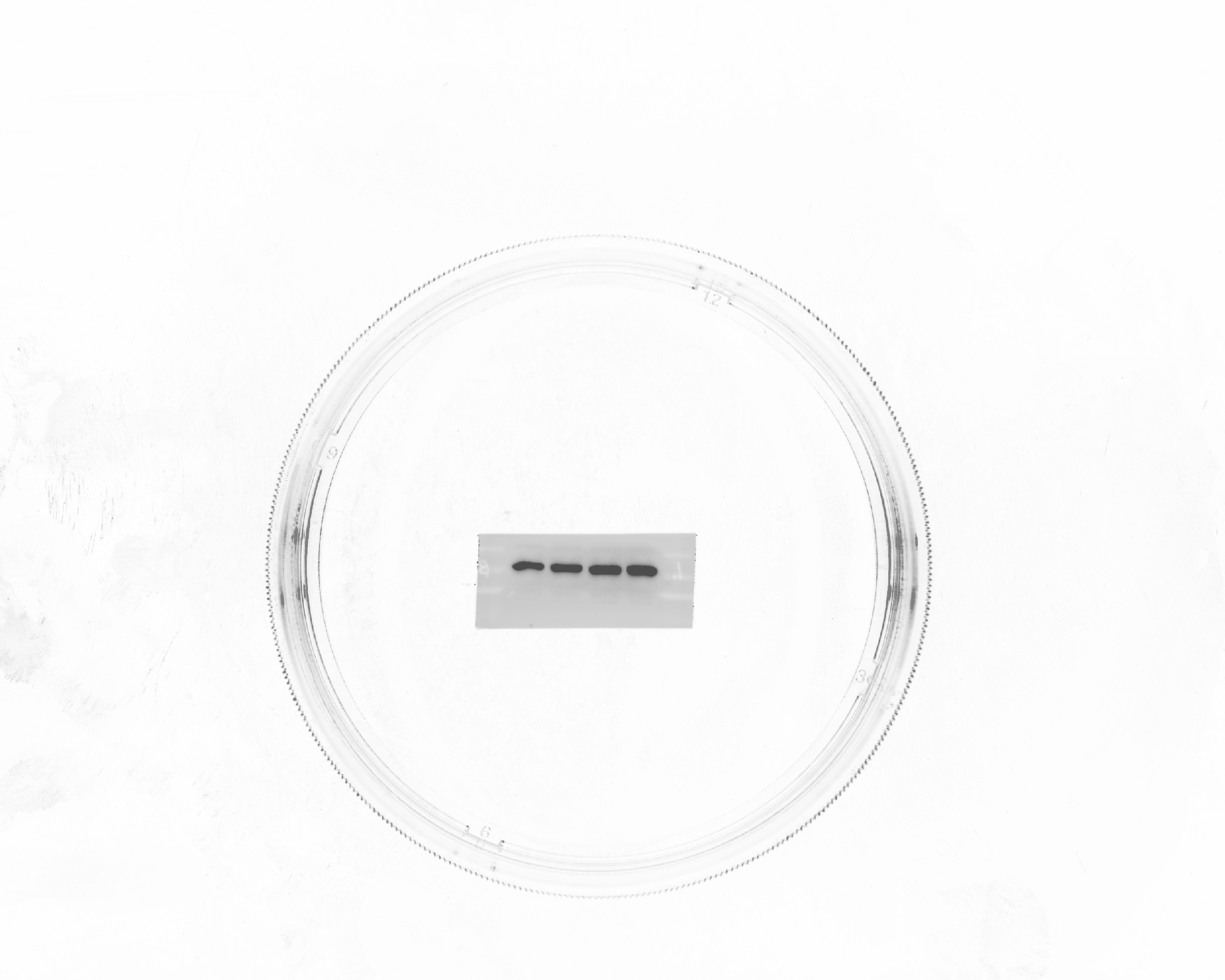

Supplement: Supplementary file 2 [file DataSheet8.zip › 1MMP9/1MMP9-beta(复合).tif]

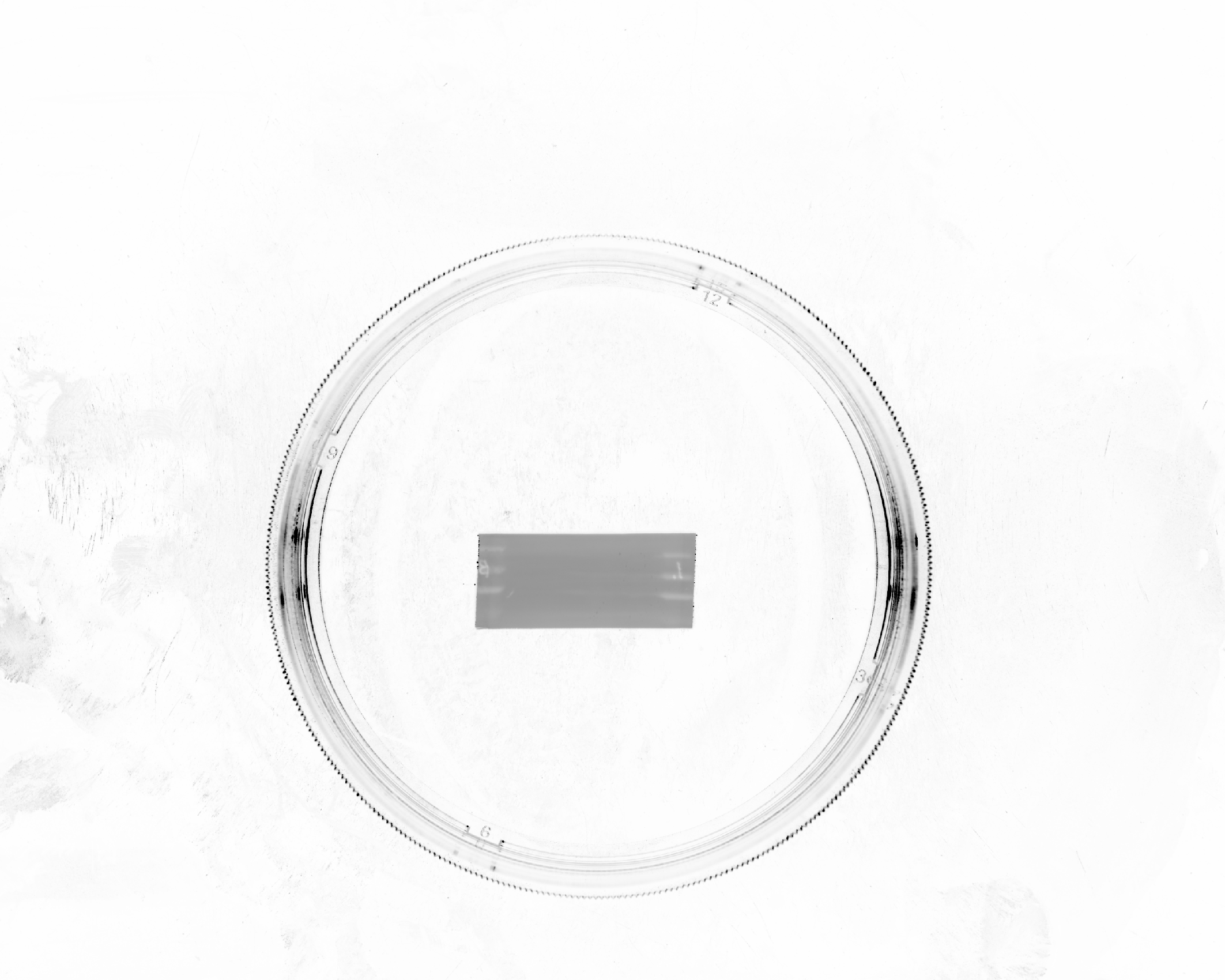

Supplement: Supplementary file 2 [file DataSheet8.zip › 1MMP9/1MMP9-beta-(Colorimetric).tif]

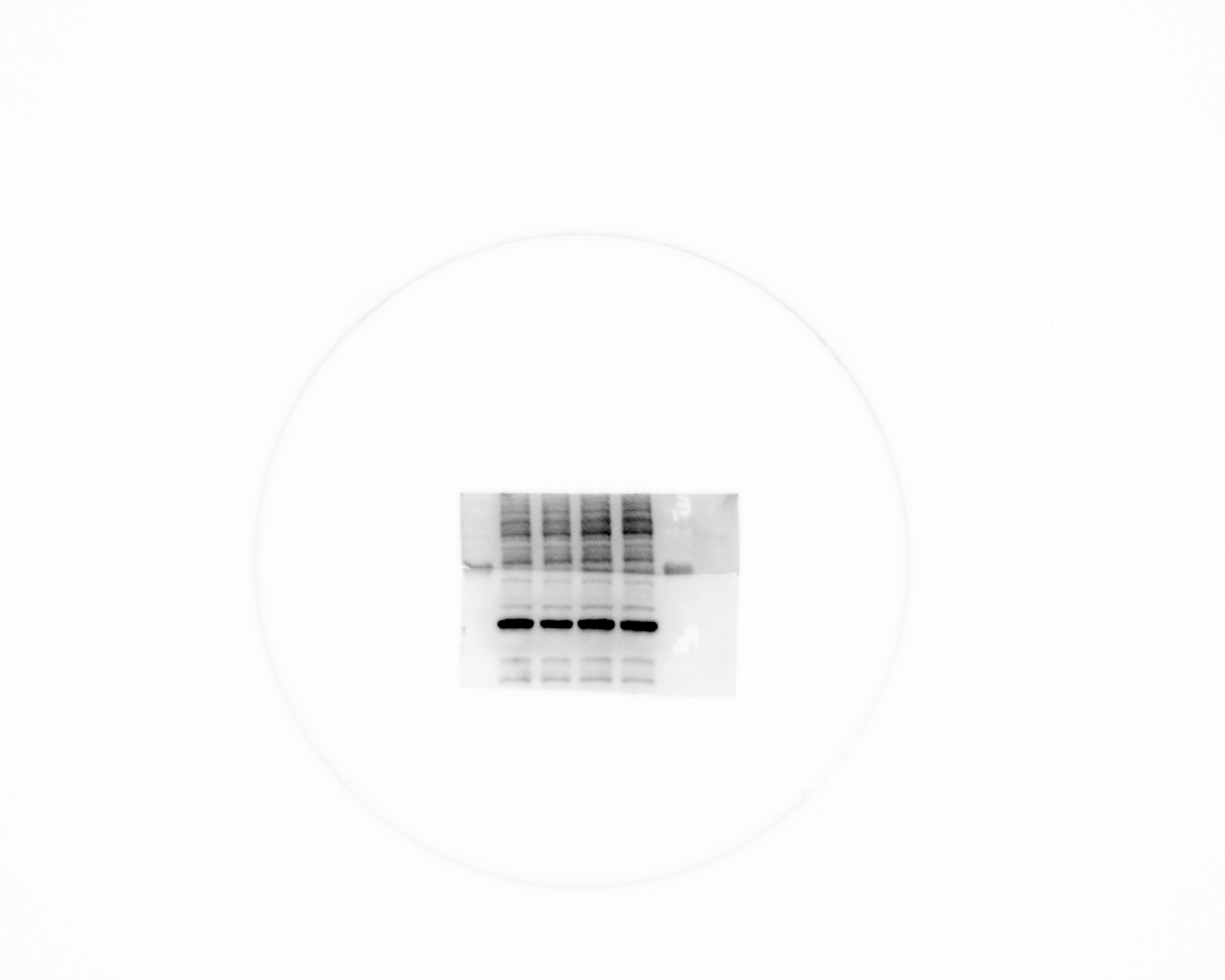

Supplement: Supplementary file 3 [file DataSheet9.zip › 2MMP9/2MMP9&beta(Chemiluminescence).tif]

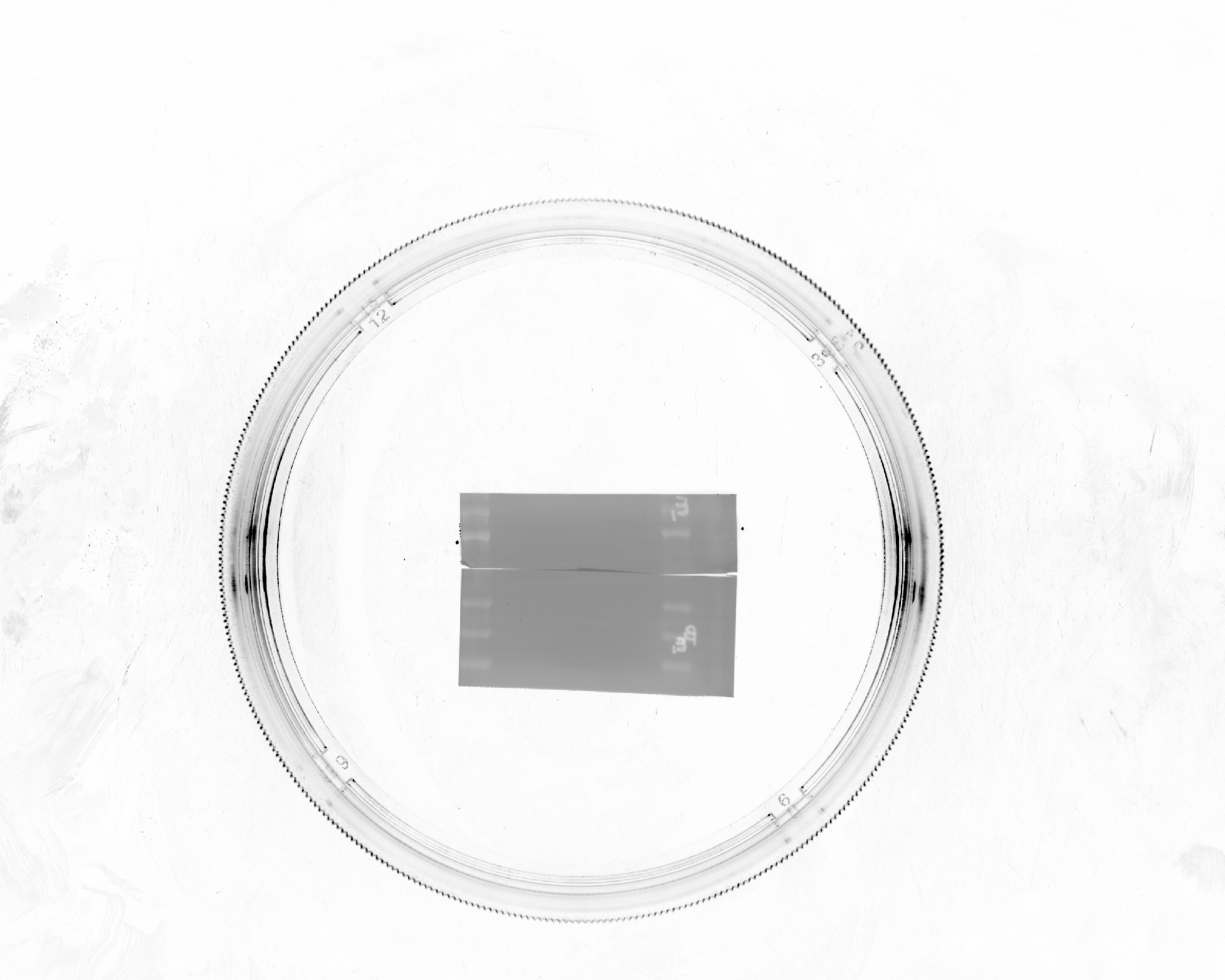

Supplement: Supplementary file 3 [file DataSheet9.zip › 2MMP9/2MMP9&beta-(Colorimetric).tif]

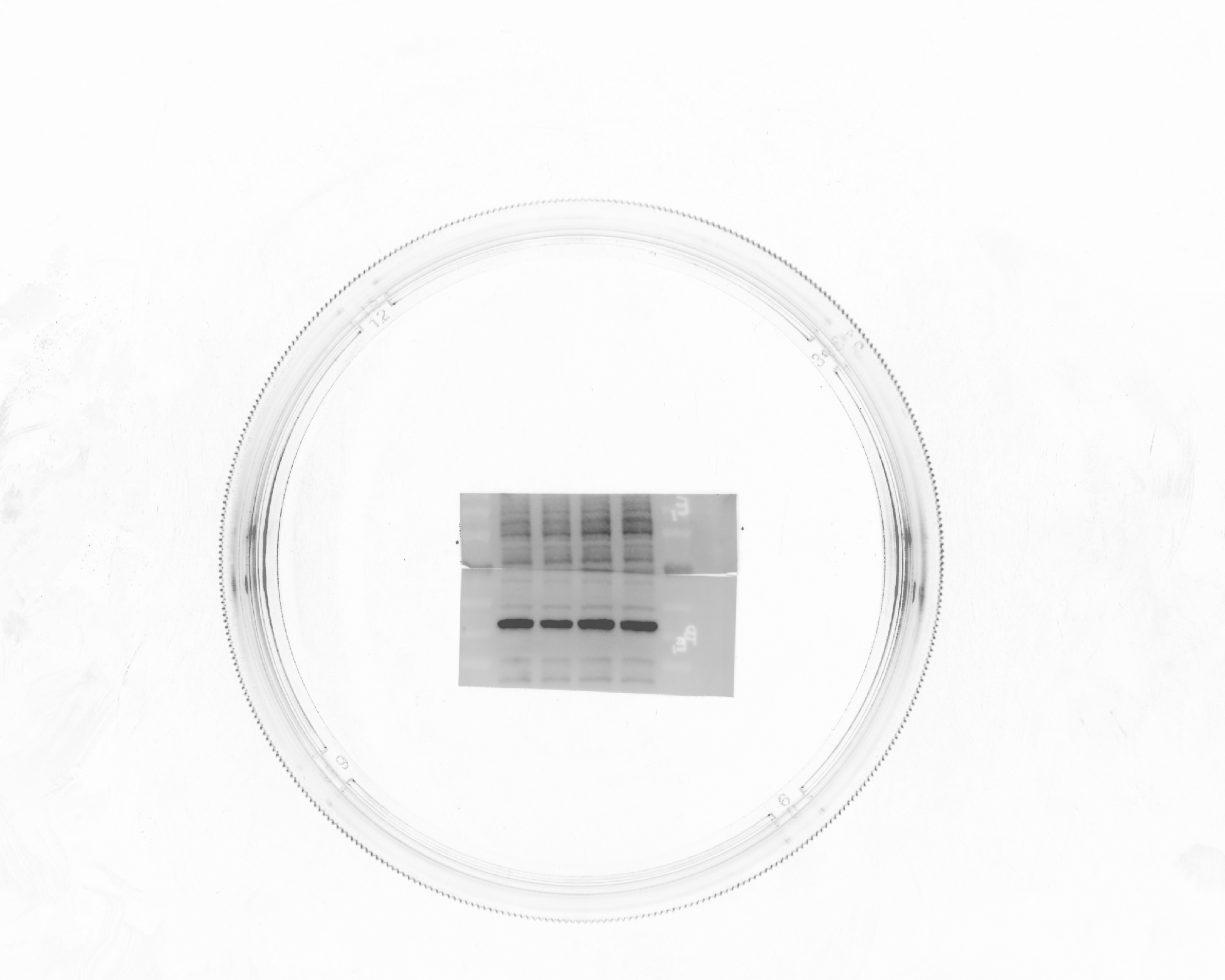

Supplement: Supplementary file 3 [file DataSheet9.zip › 2MMP9/2MMP9&beta-(复合).tif]

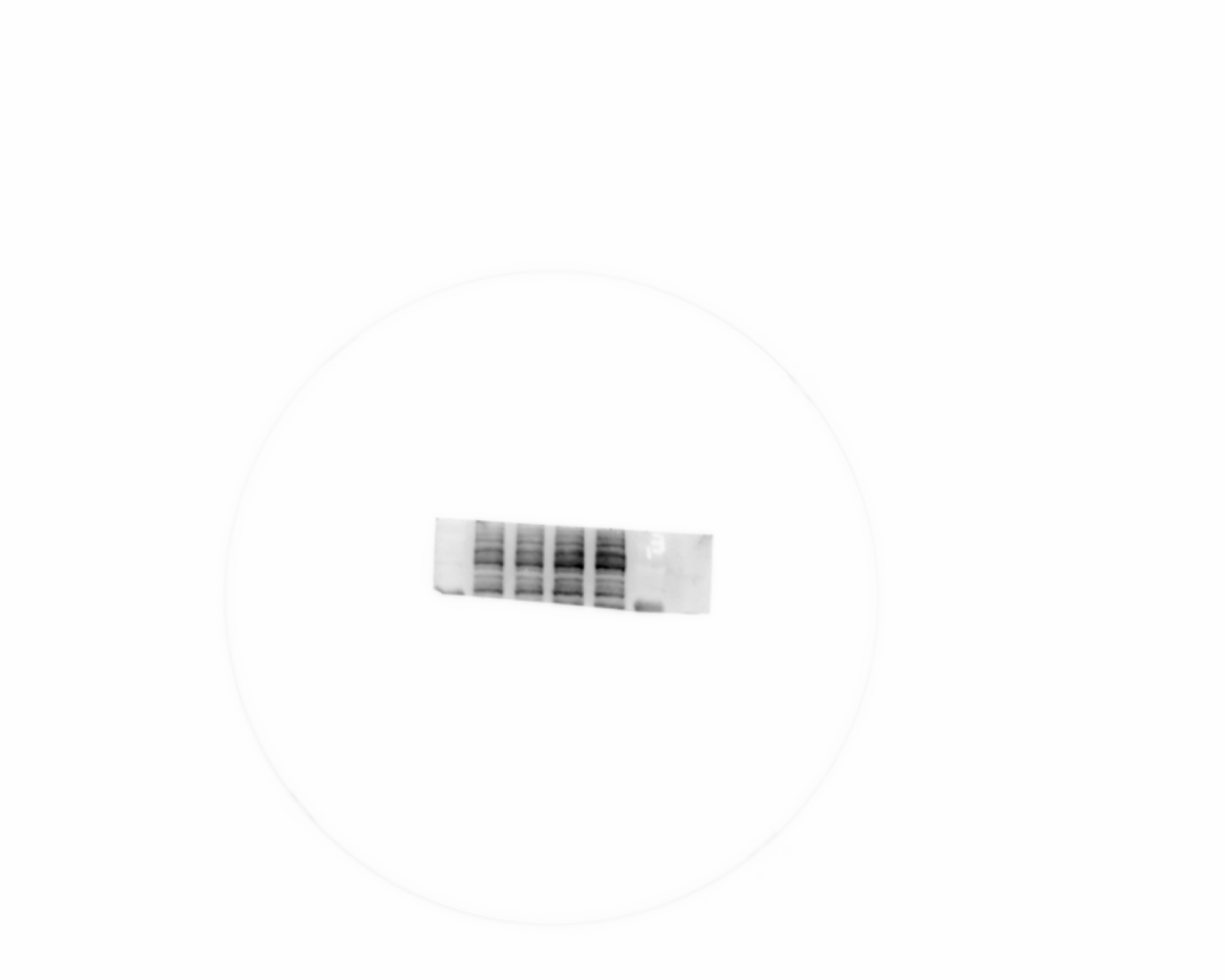

Supplement: Supplementary file 3 [file DataSheet9.zip › 2MMP9/2MMP9(Chemiluminescence).tif]

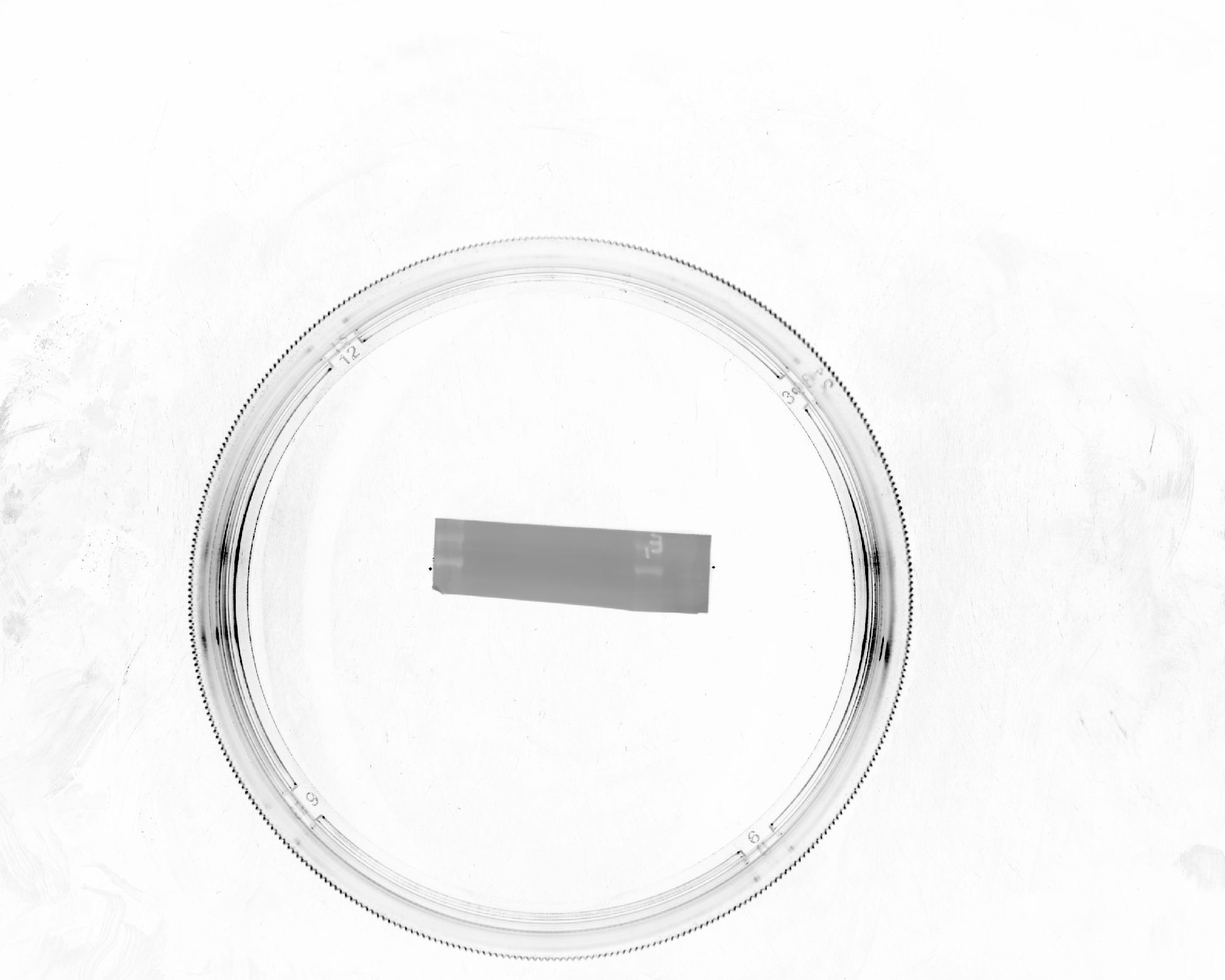

Supplement: Supplementary file 3 [file DataSheet9.zip › 2MMP9/2MMP9-(Colorimetric).tif]

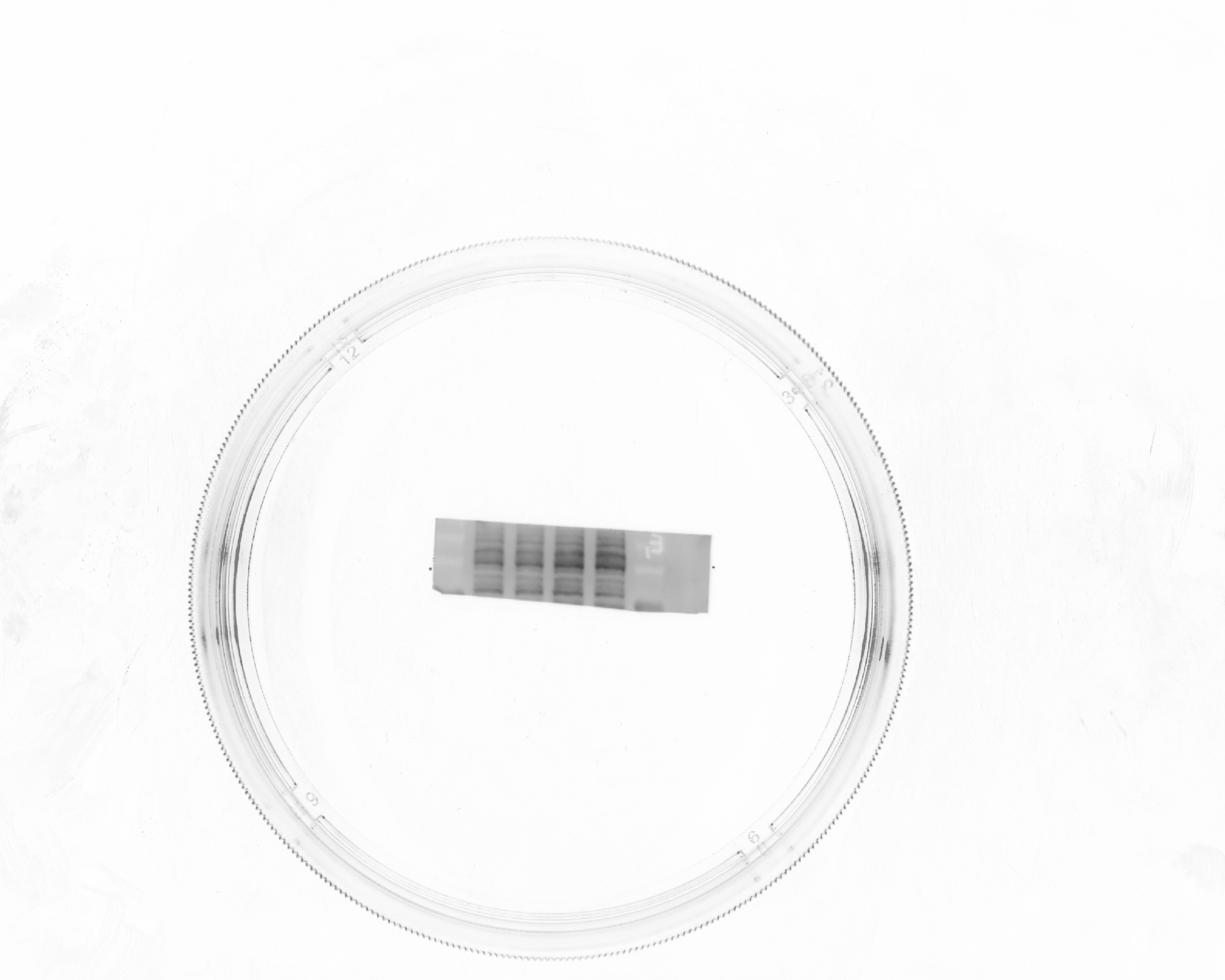

Supplement: Supplementary file 3 [file DataSheet9.zip › 2MMP9/2MMP9-(复合).tif]

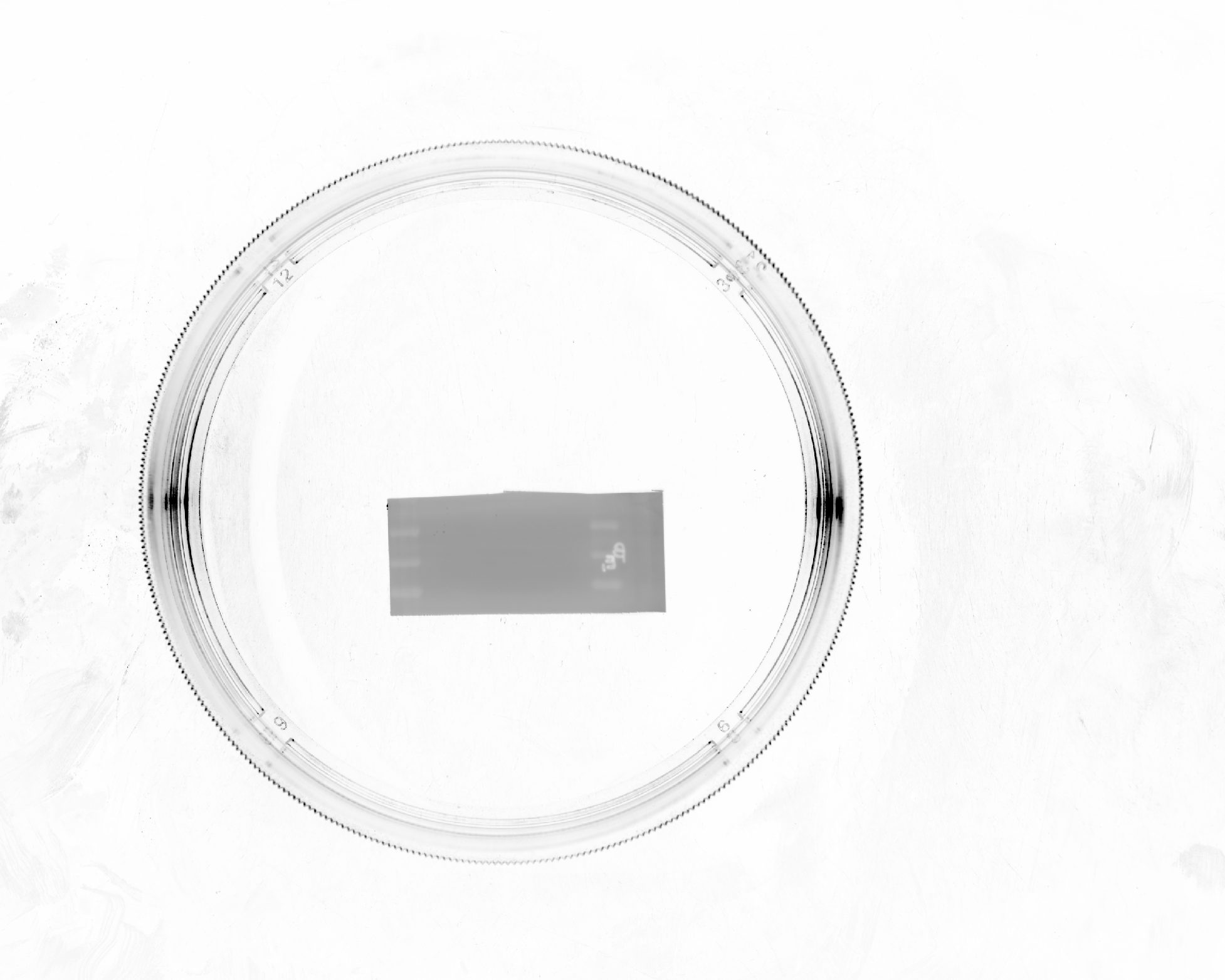

Supplement: Supplementary file 3 [file DataSheet9.zip › 2MMP9/2MMP9-beta-(Colorimetric).tif]

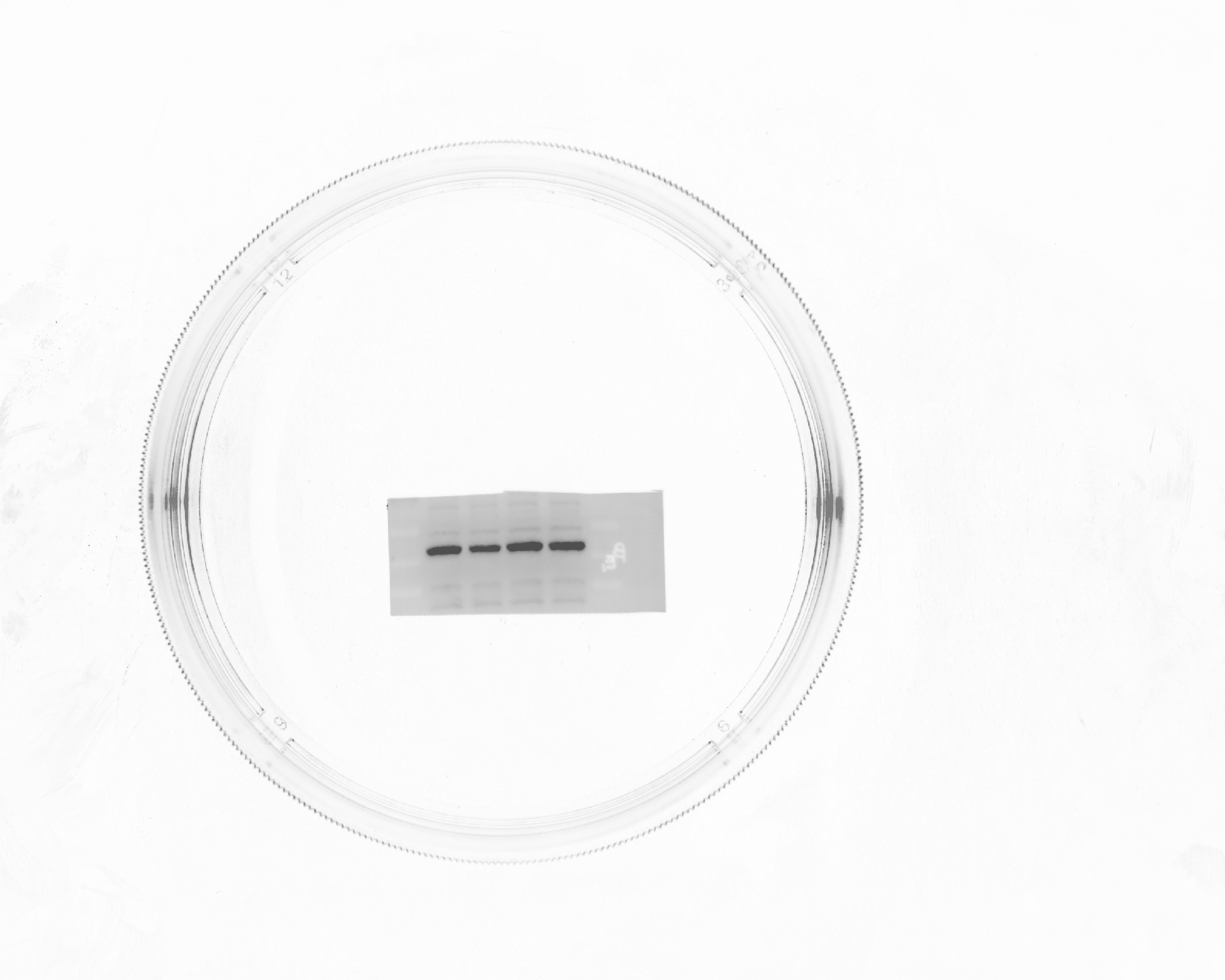

Supplement: Supplementary file 3 [file DataSheet9.zip › 2MMP9/2MMP9-beta-(复合).tif]

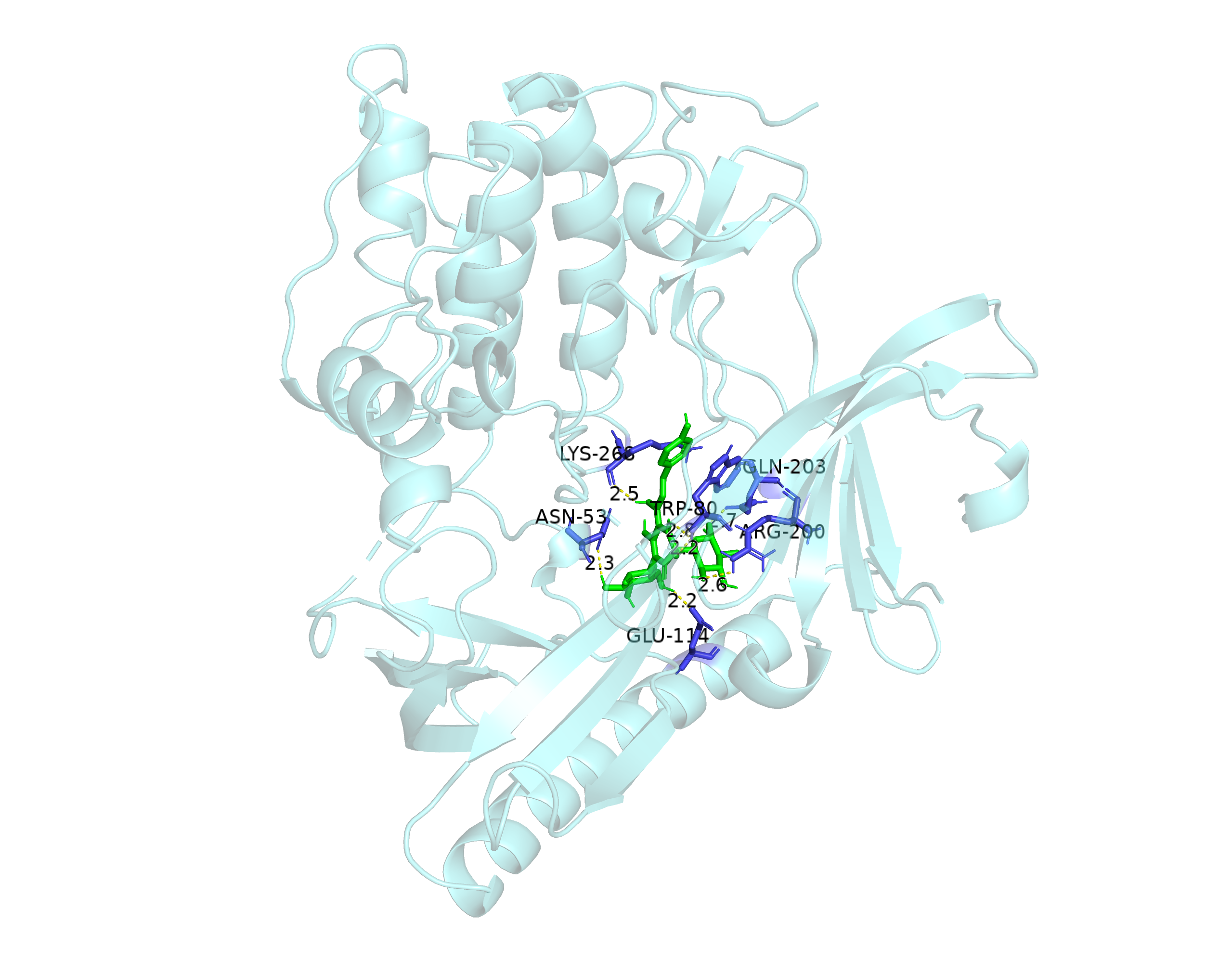

Supplement: Supplementary file 4 [file DataSheet4.zip › Figure4-original data/A-A1/AKT1大.png]

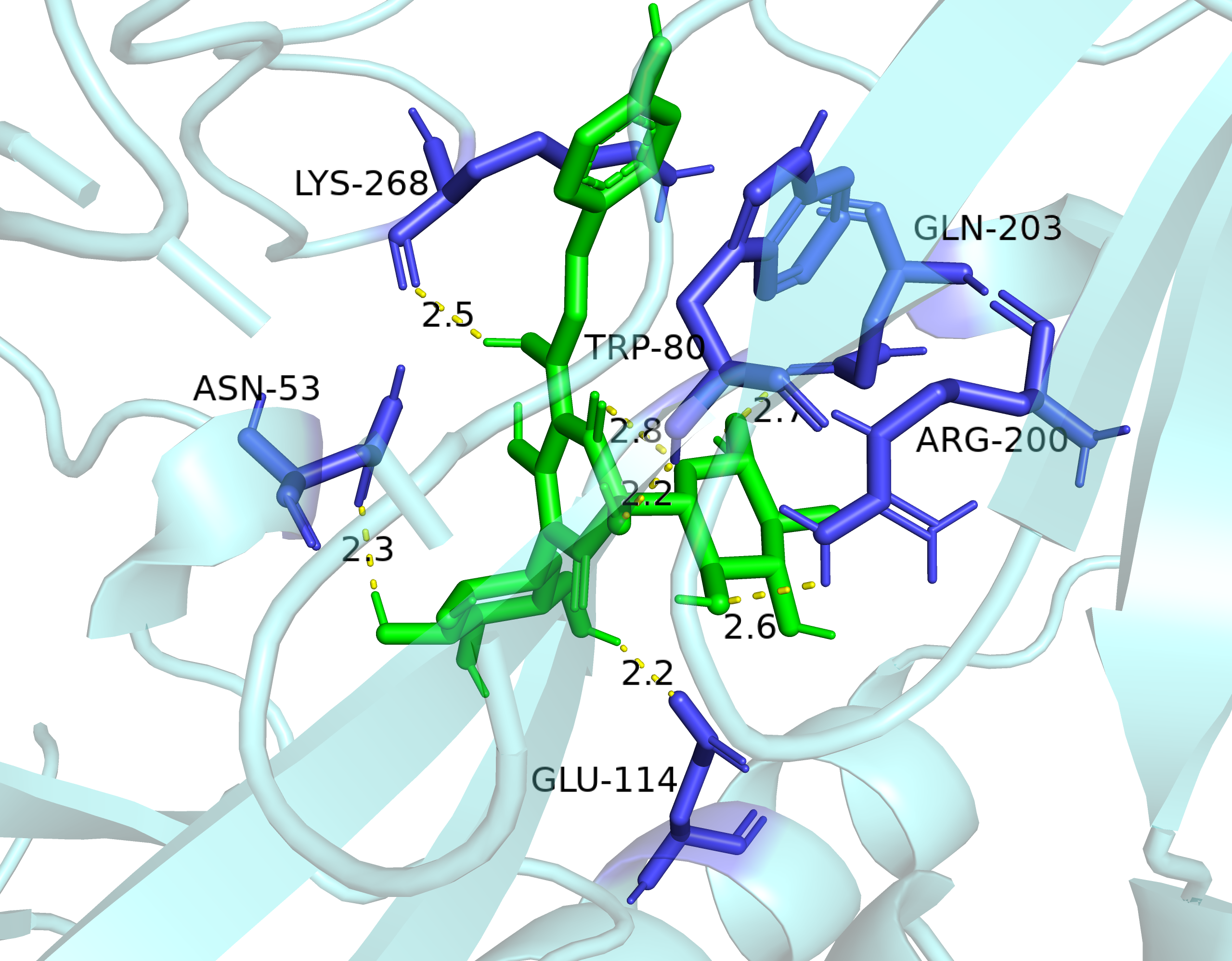

Supplement: Supplementary file 4 [file DataSheet4.zip › Figure4-original data/A-A1/AKT1小.png]

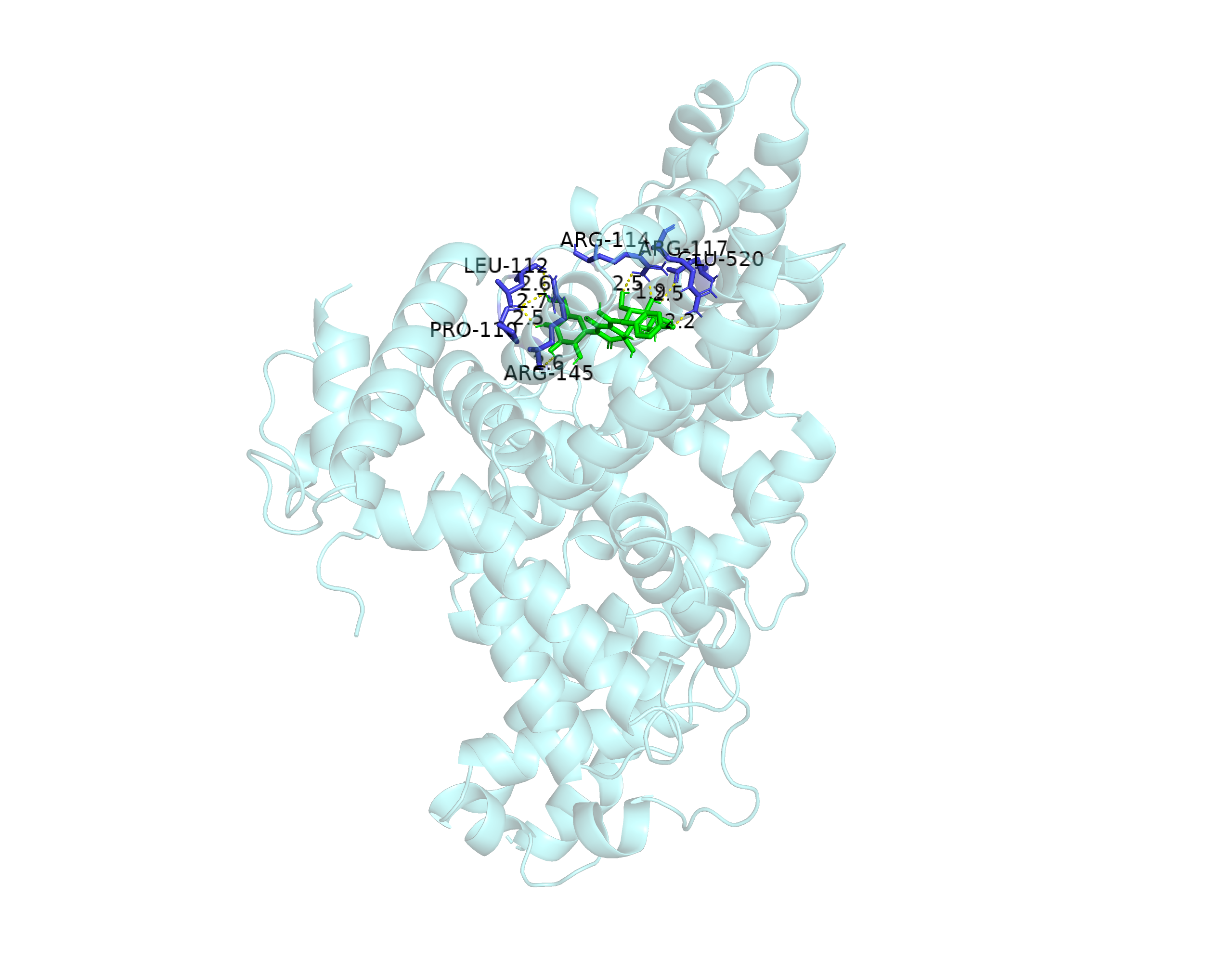

Supplement: Supplementary file 4 [file DataSheet4.zip › Figure4-original data/B-B1/ALB大.png]

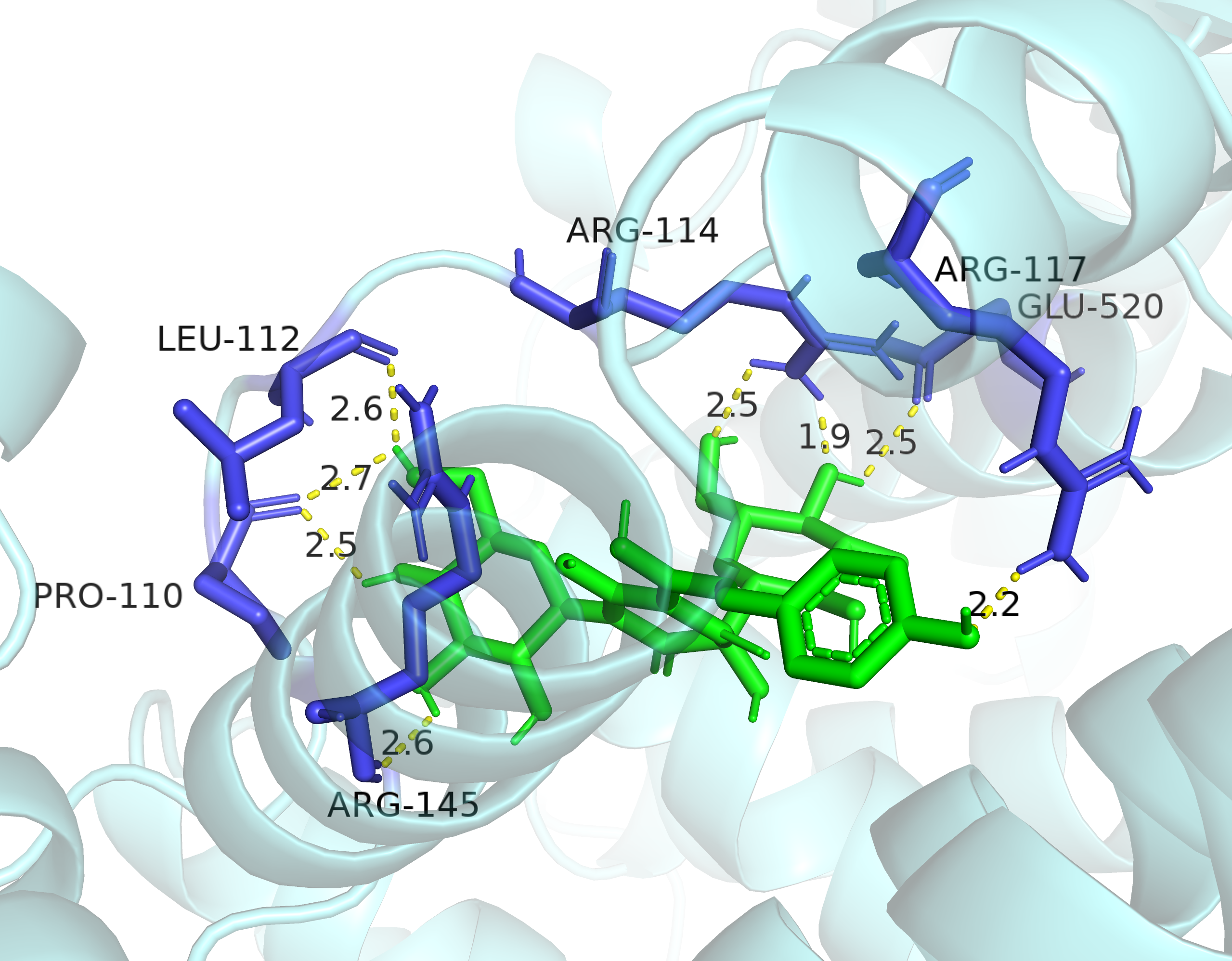

Supplement: Supplementary file 4 [file DataSheet4.zip › Figure4-original data/B-B1/ALB小.png]

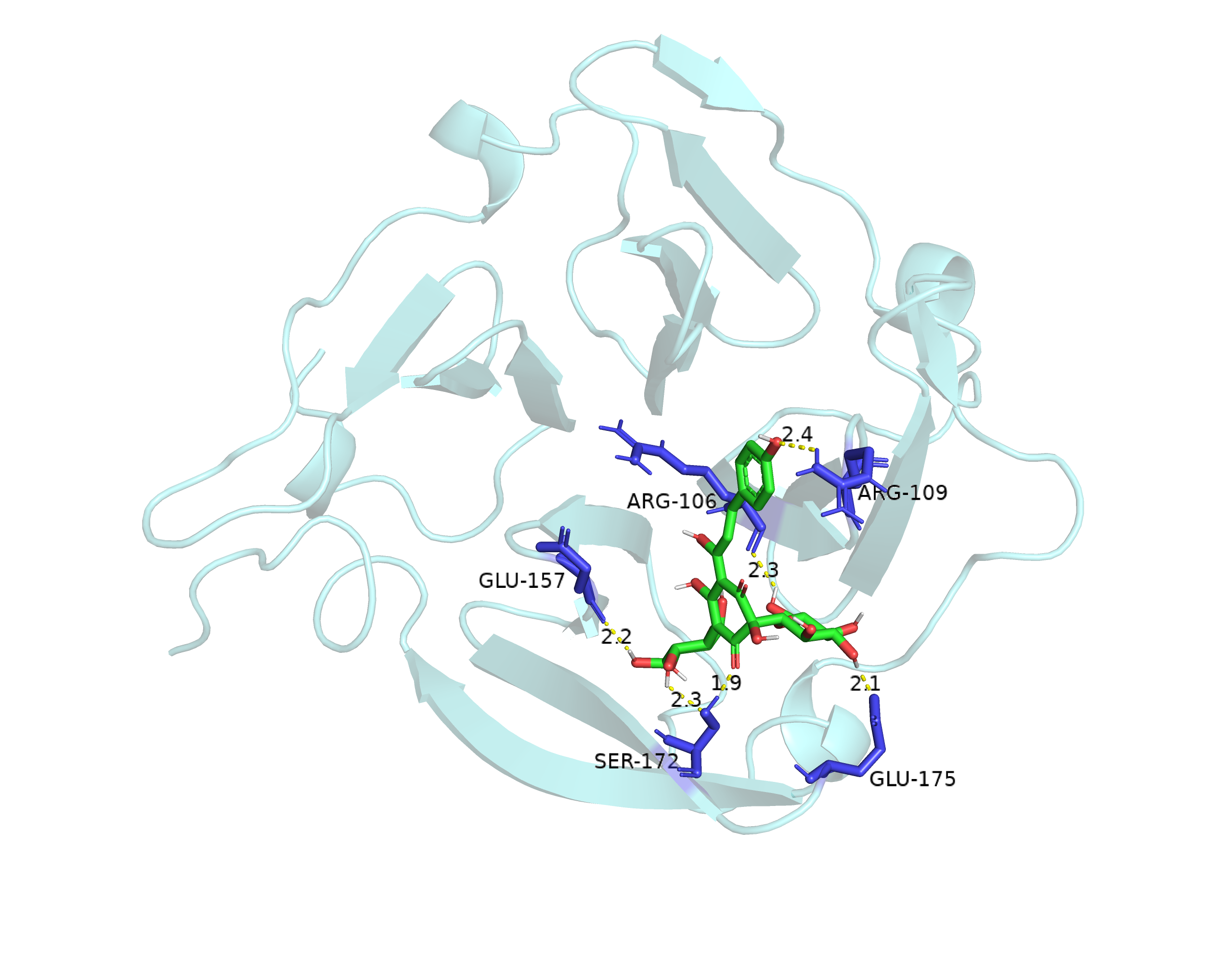

Supplement: Supplementary file 4 [file DataSheet4.zip › Figure4-original data/C-C1/MMP9大.png]

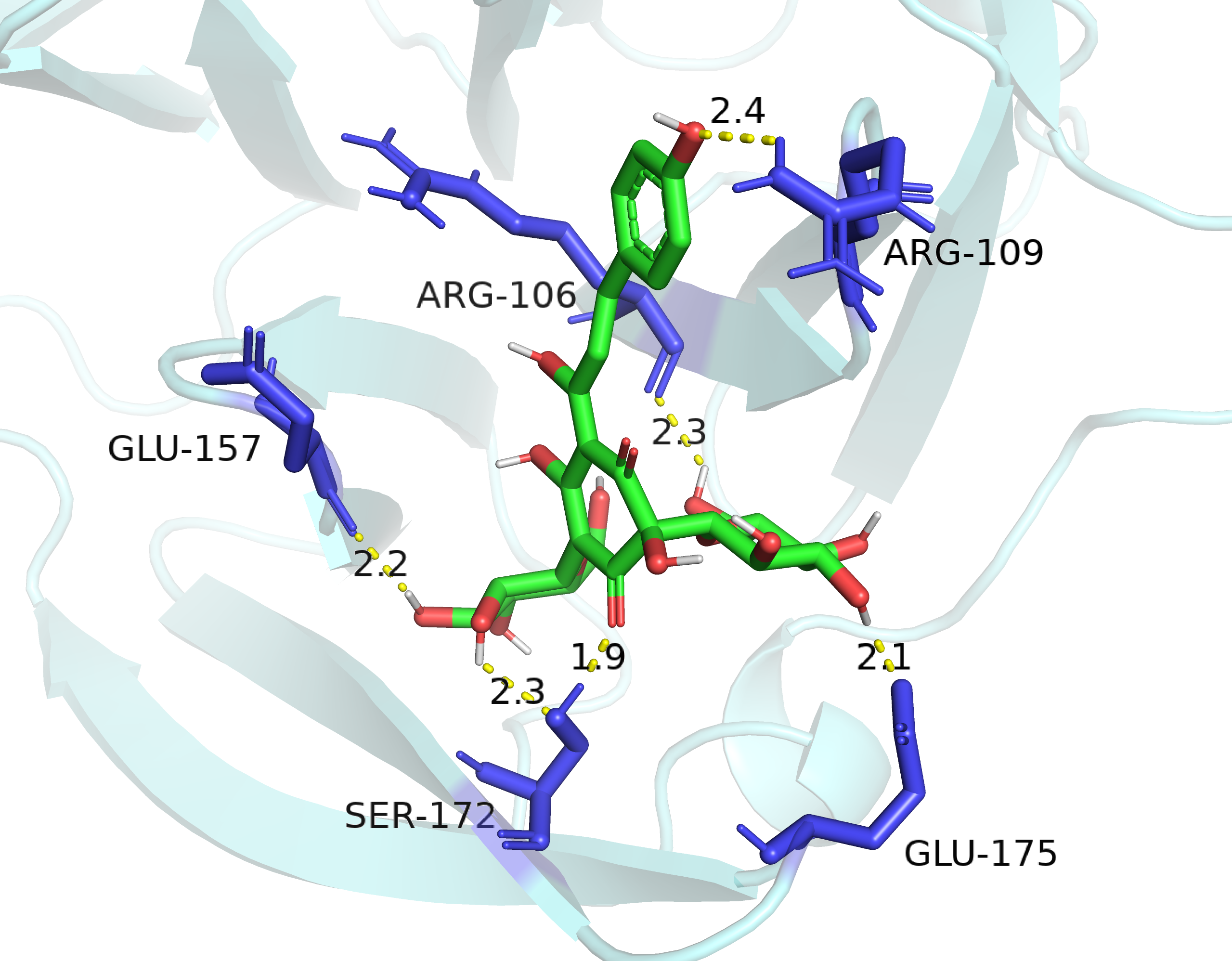

Supplement: Supplementary file 4 [file DataSheet4.zip › Figure4-original data/C-C1/MMP9小.png]

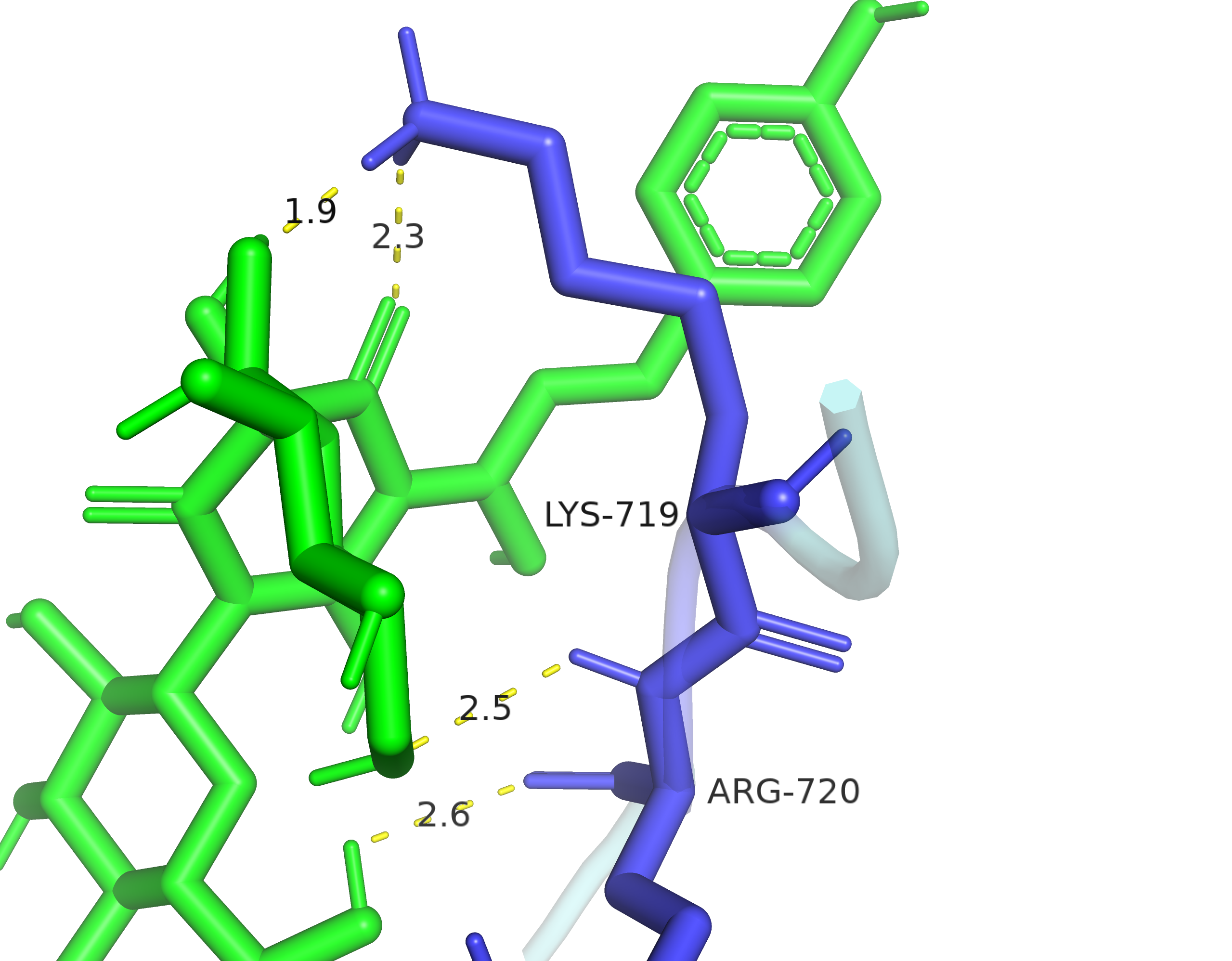

Supplement: Supplementary file 4 [file DataSheet4.zip › Figure4-original data/D-D1/HIF1A小.png]

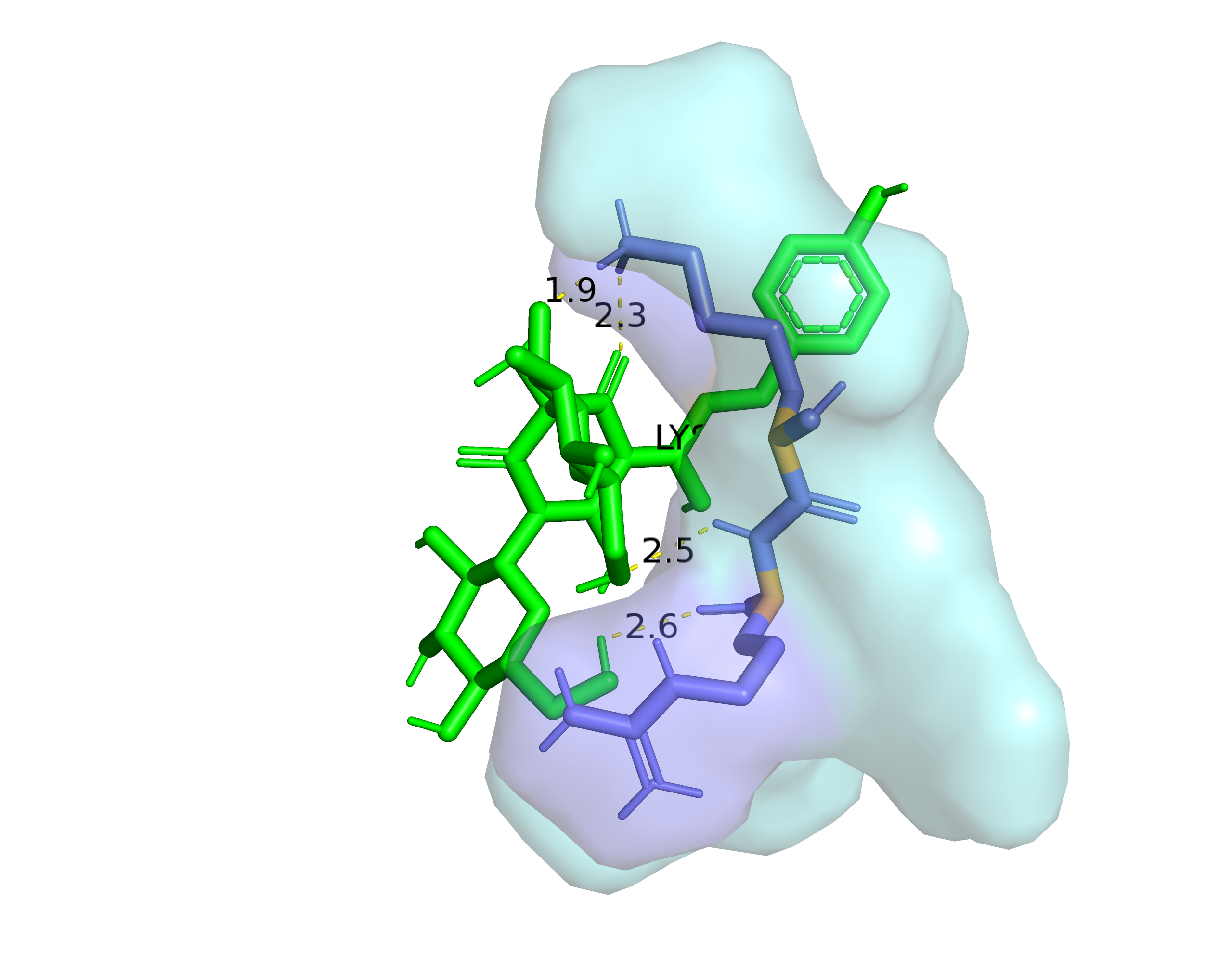

Supplement: Supplementary file 4 [file DataSheet4.zip › Figure4-original data/D-D1/HIFA1大.png]

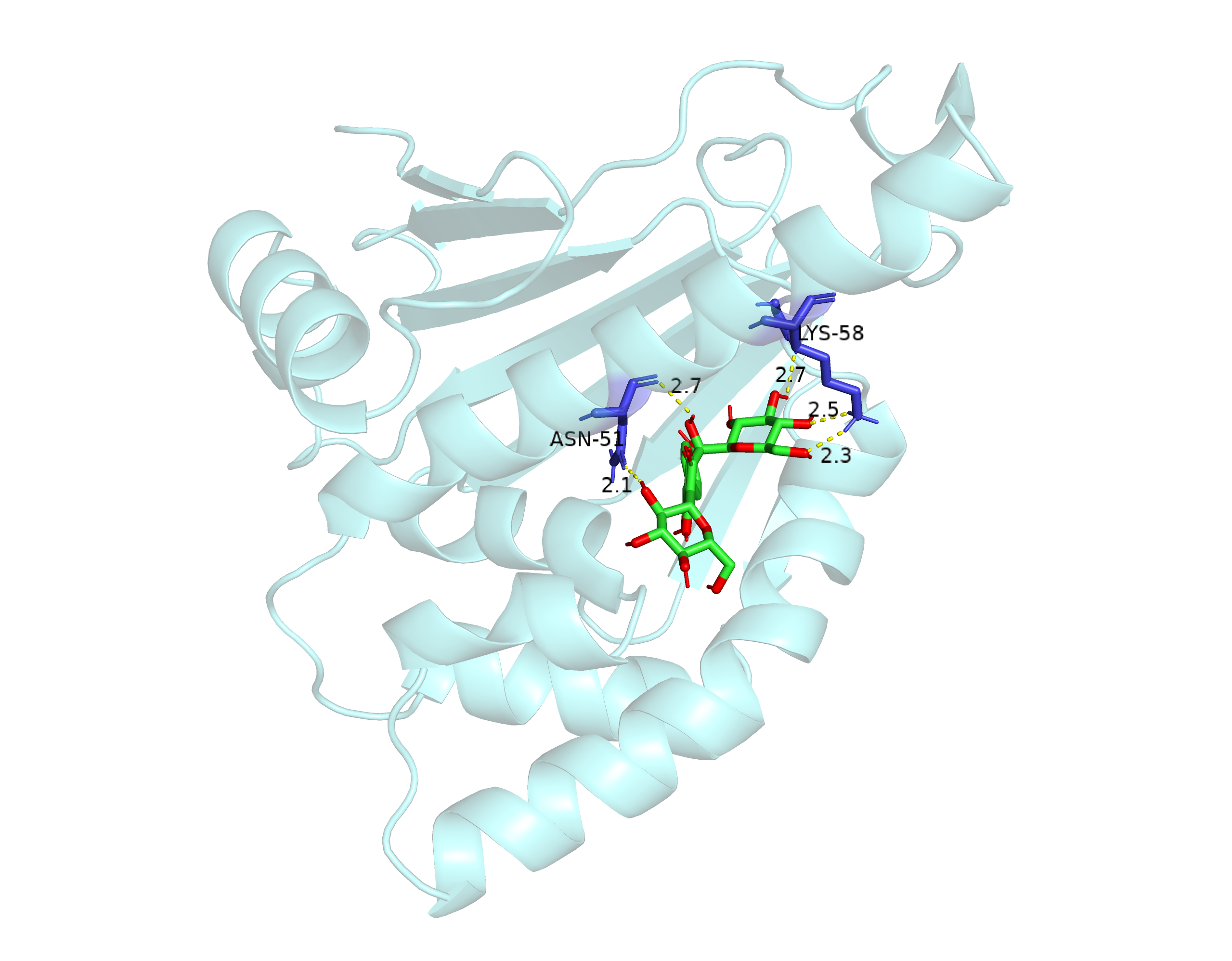

Supplement: Supplementary file 4 [file DataSheet4.zip › Figure4-original data/E-E1/HAA1大.png]

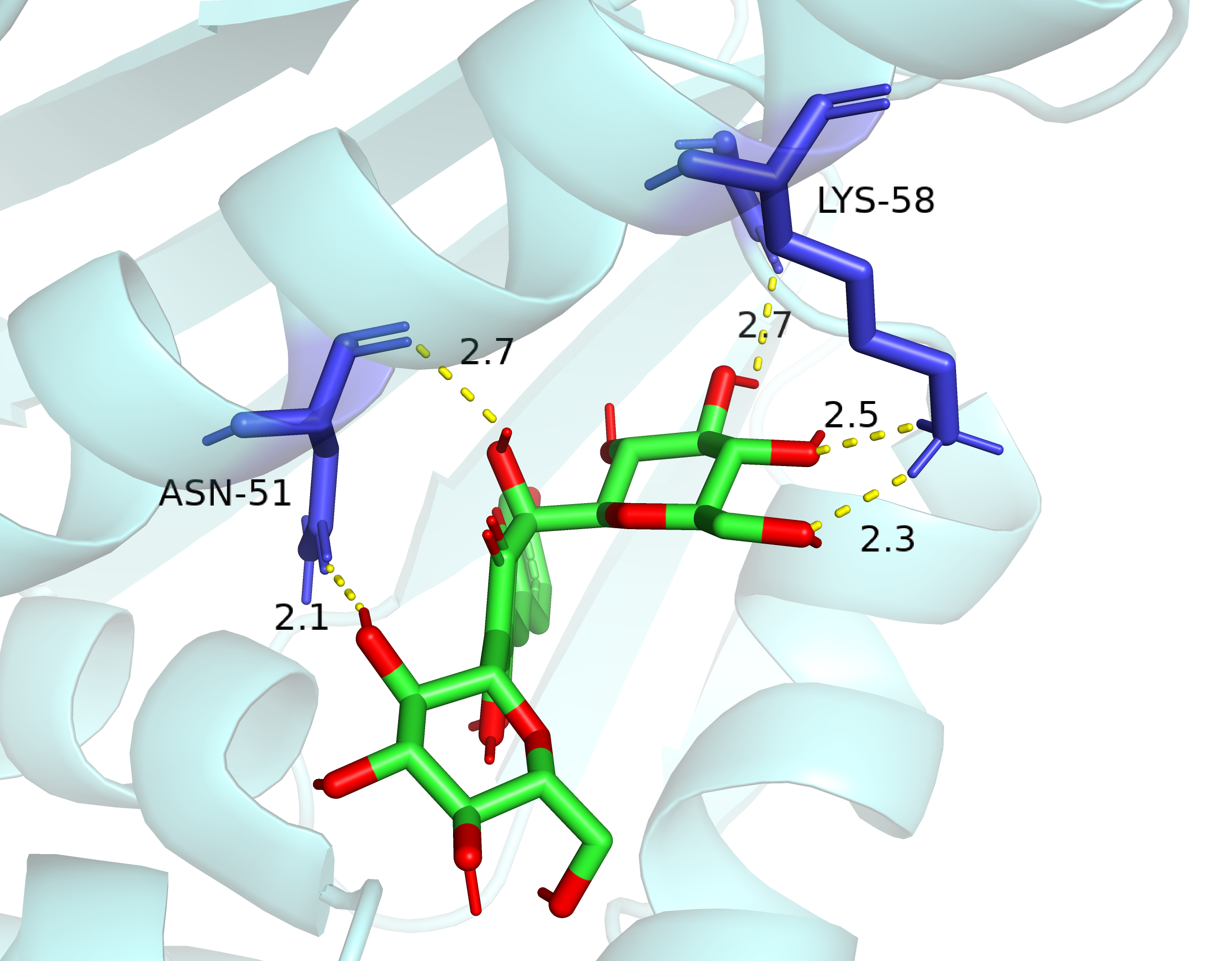

Supplement: Supplementary file 4 [file DataSheet4.zip › Figure4-original data/E-E1/HAA1小.png]

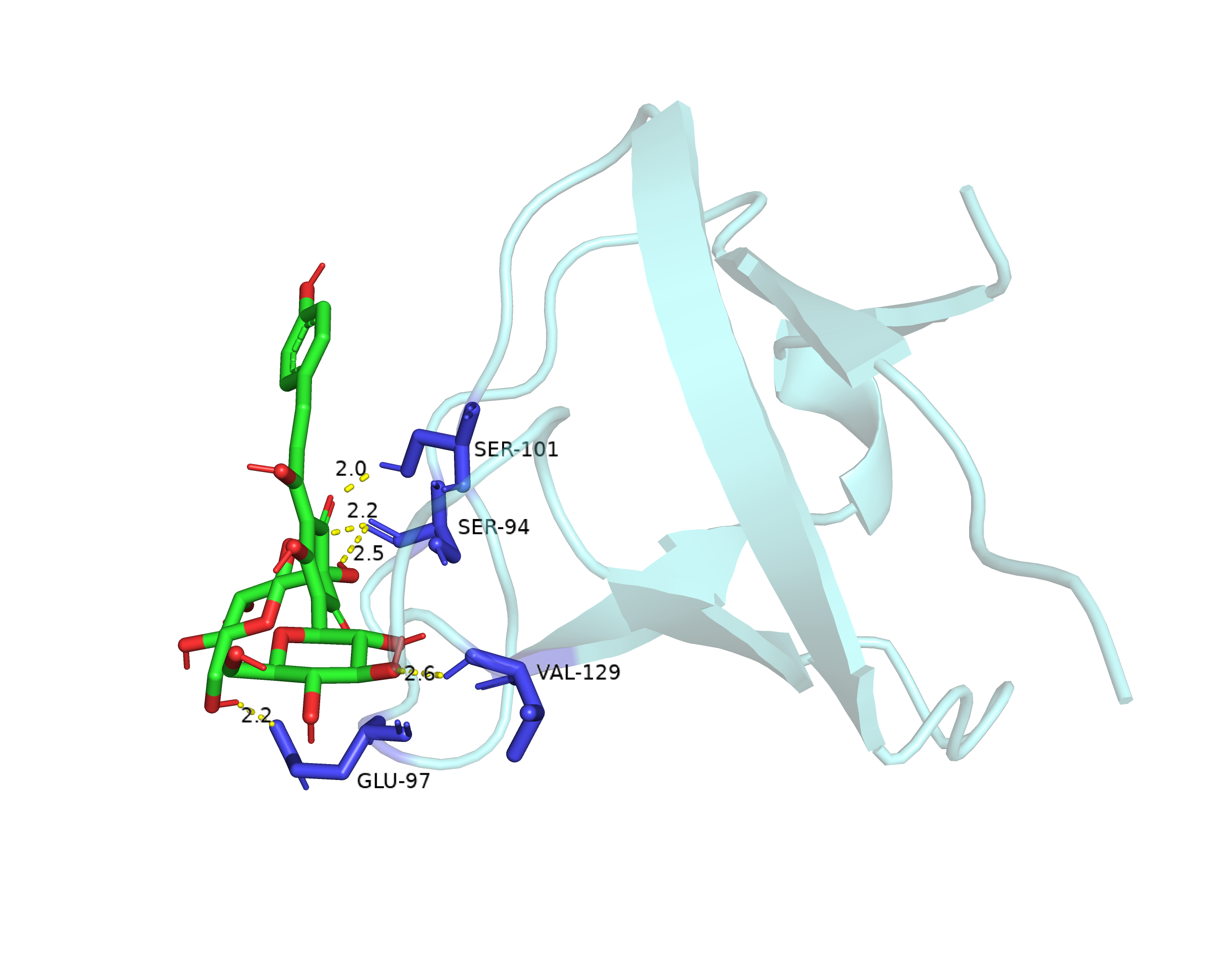

Supplement: Supplementary file 4 [file DataSheet4.zip › Figure4-original data/F-F1/SRC大.png]

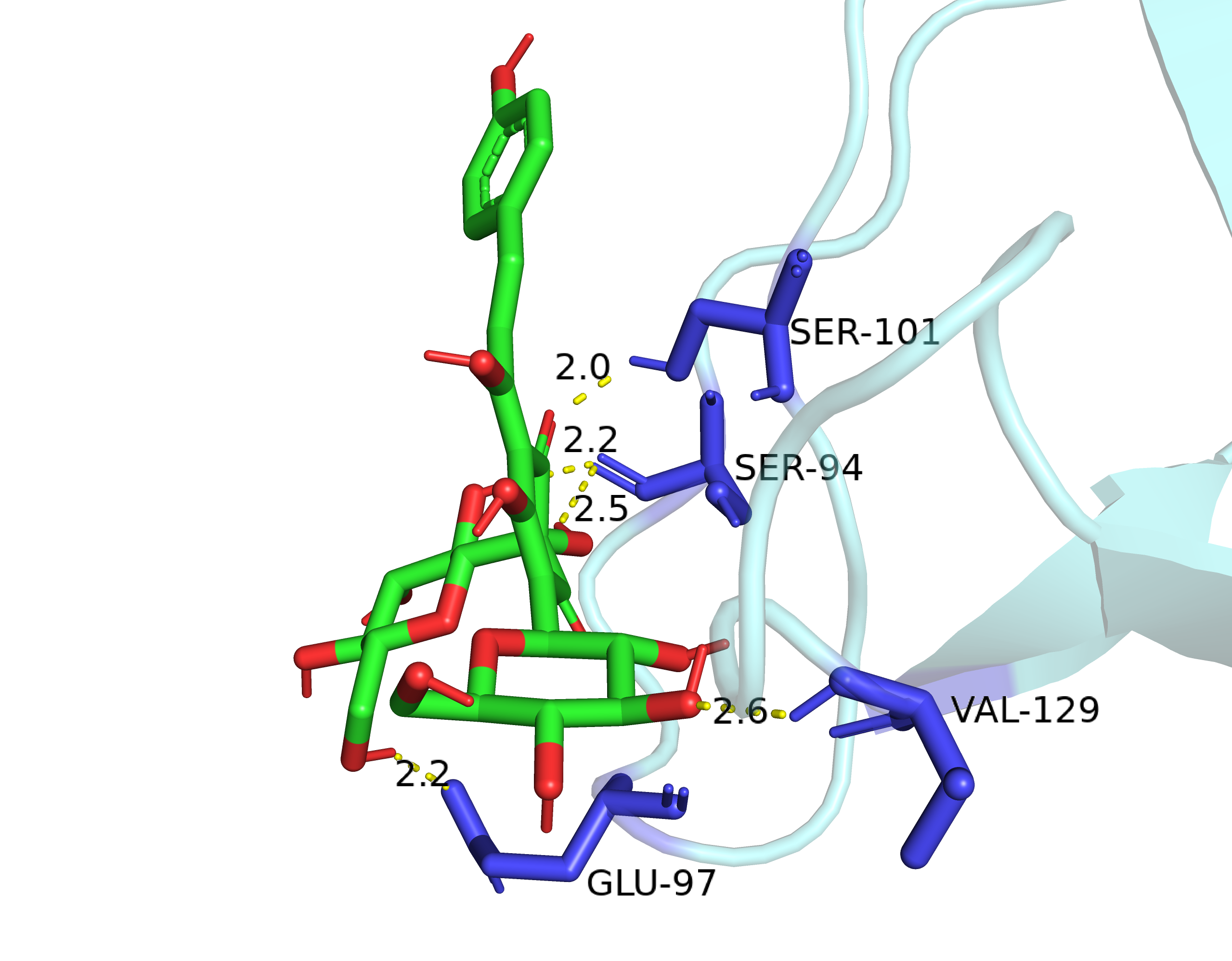

Supplement: Supplementary file 4 [file DataSheet4.zip › Figure4-original data/F-F1/SRC小.png]

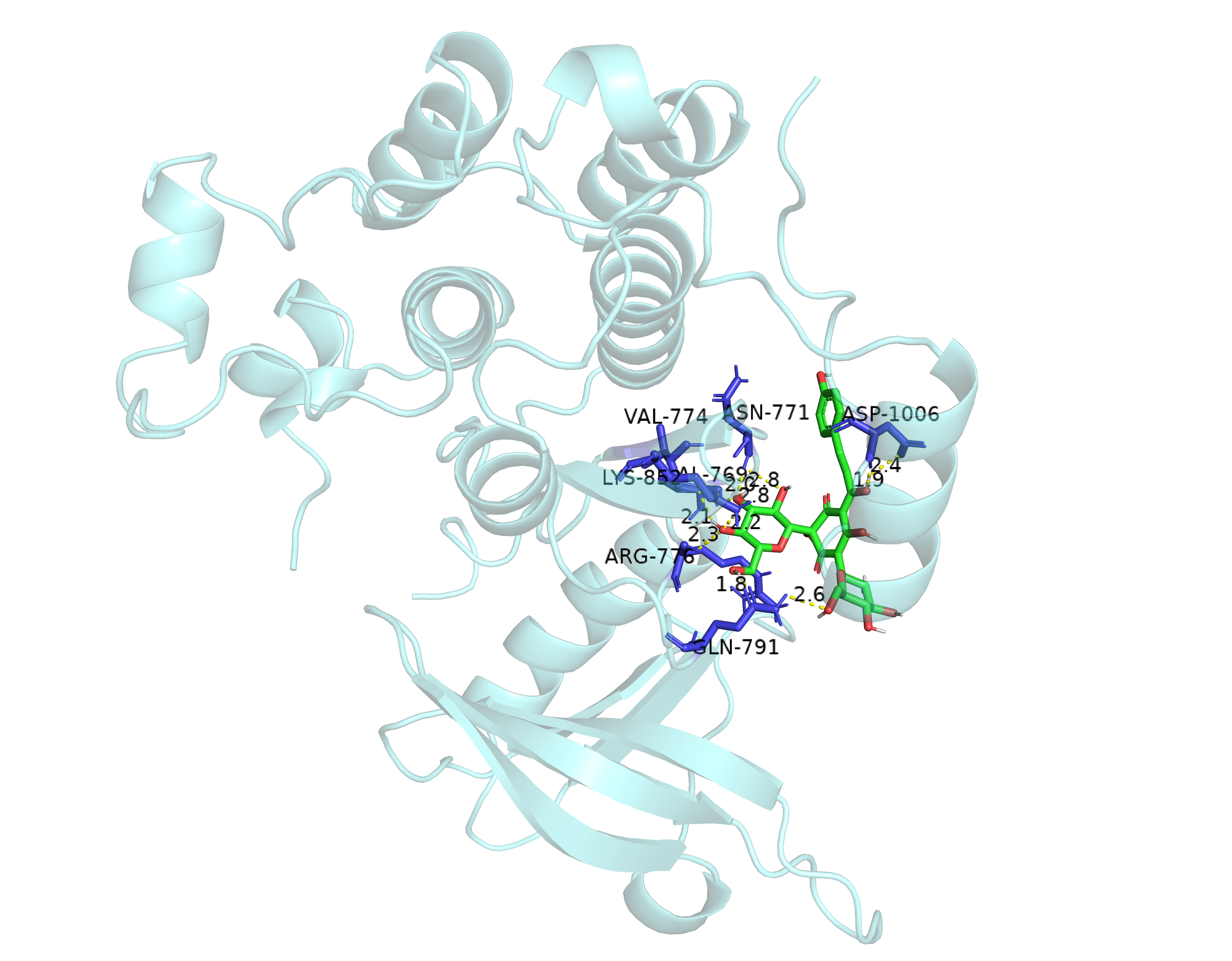

Supplement: Supplementary file 4 [file DataSheet4.zip › Figure4-original data/G-G1/EGFR大.png]

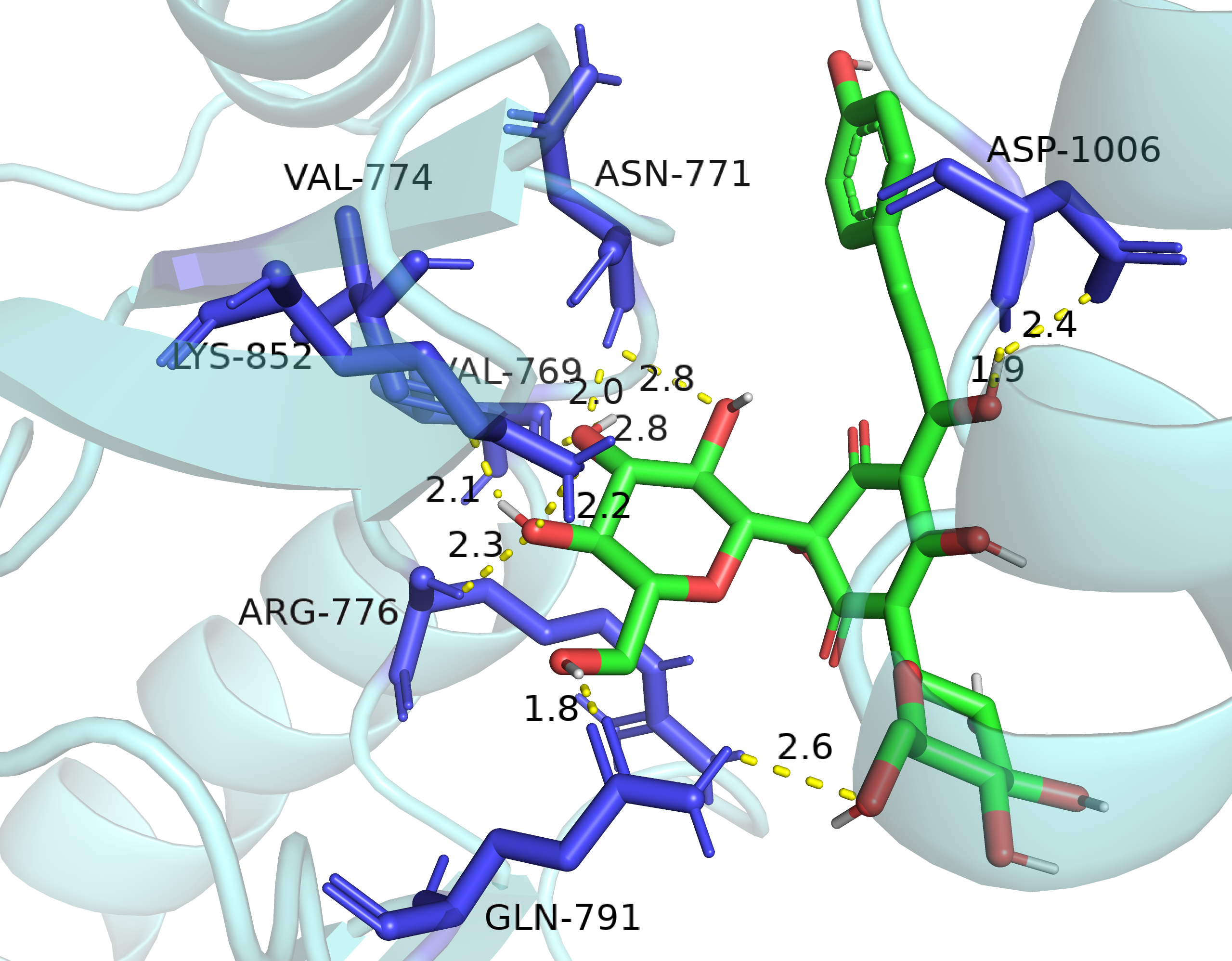

Supplement: Supplementary file 4 [file DataSheet4.zip › Figure4-original data/G-G1/EGFR小.png]

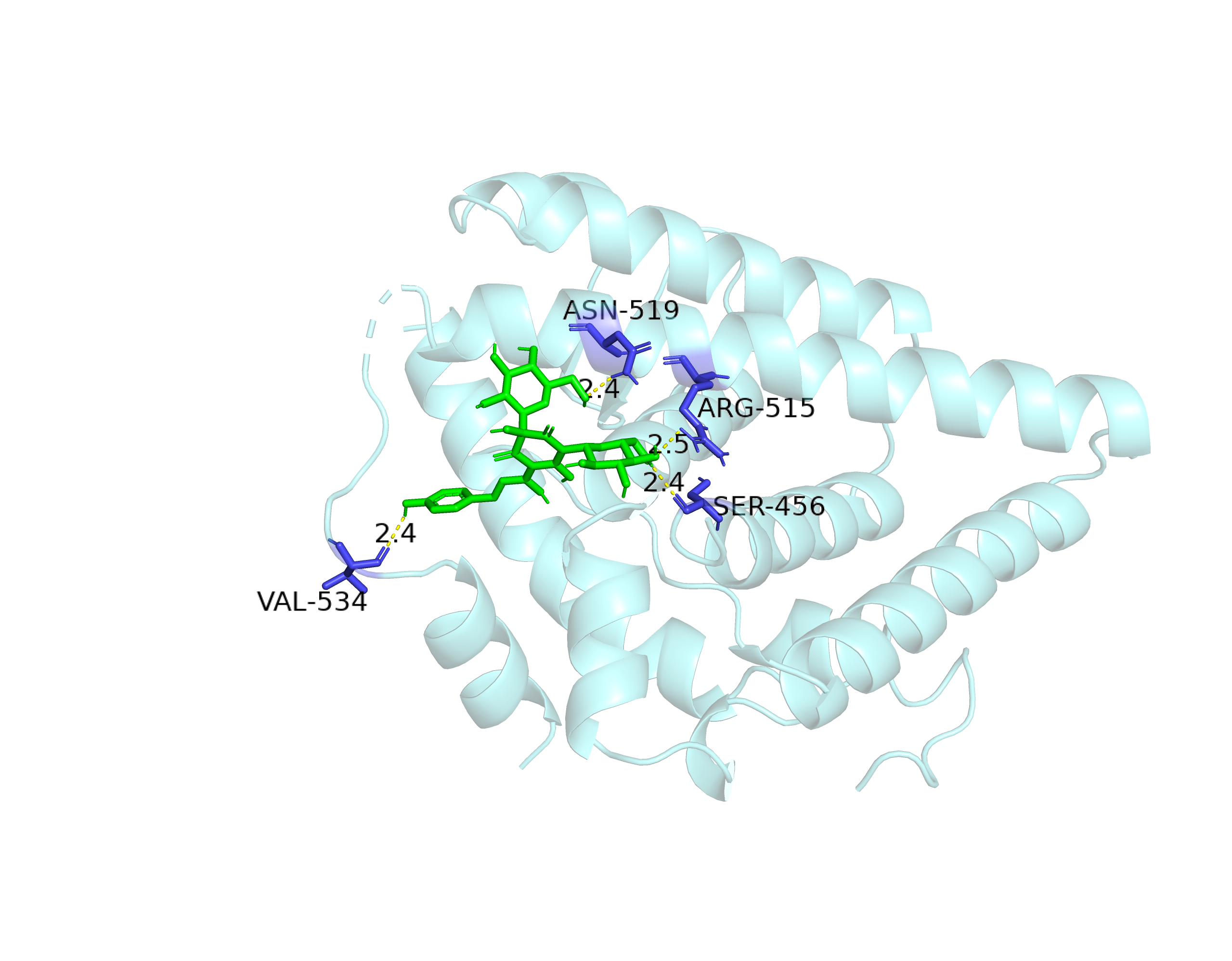

Supplement: Supplementary file 4 [file DataSheet4.zip › Figure4-original data/H-H1/ESR1大.png]

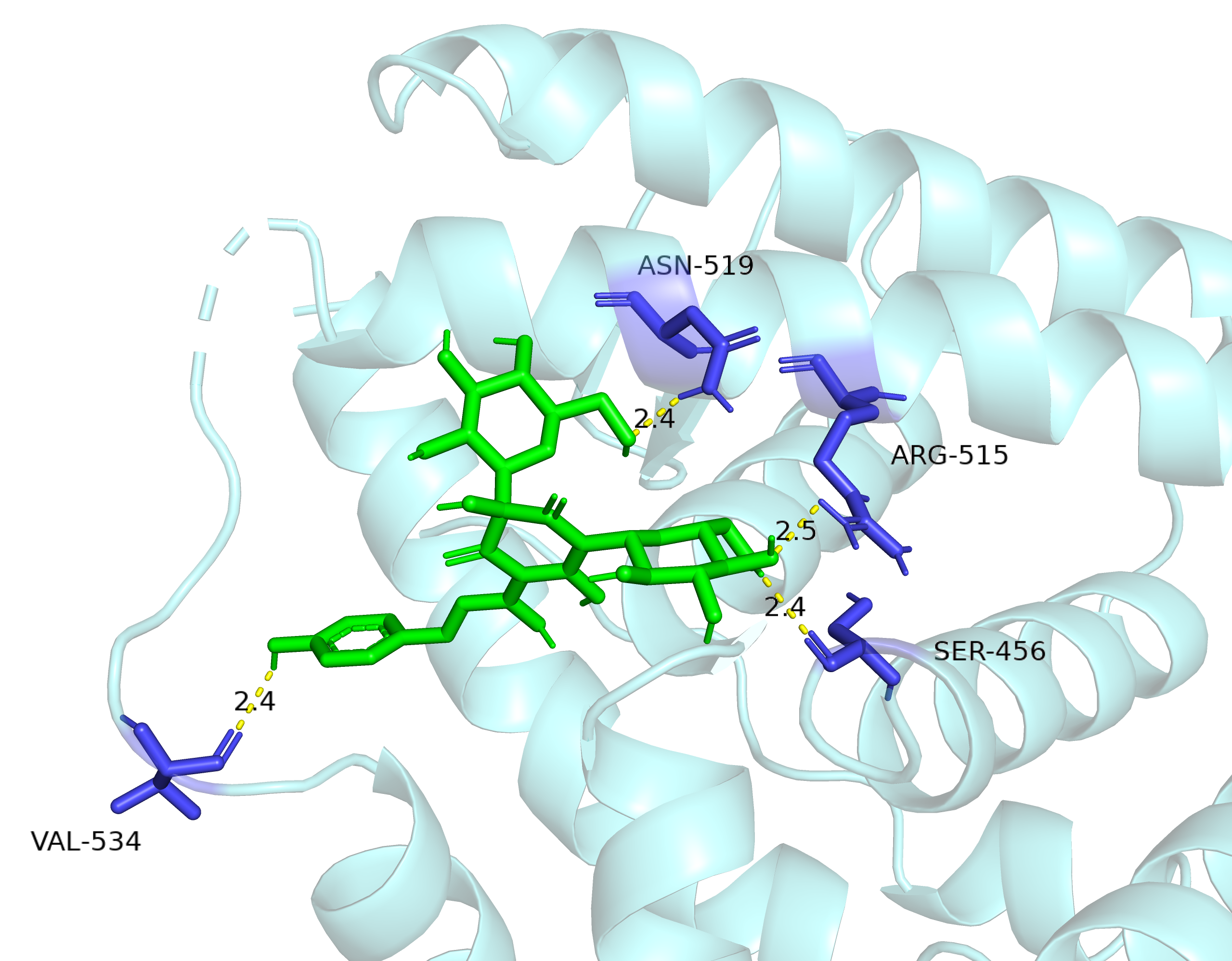

Supplement: Supplementary file 4 [file DataSheet4.zip › Figure4-original data/H-H1/ESR1小.png]

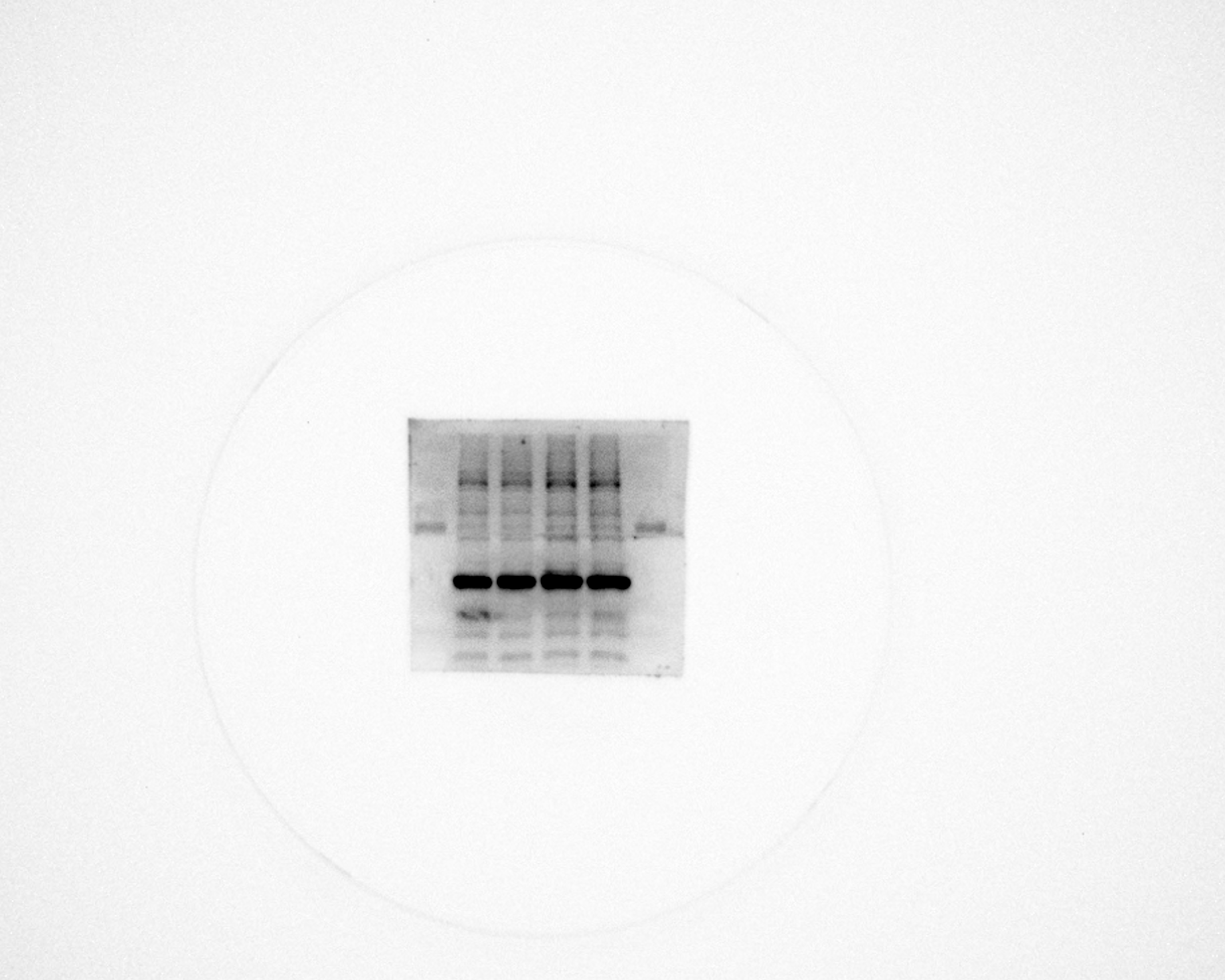

Supplement: Supplementary file 6 [file DataSheet10.zip › 3MMP9/3MMP9&beta(Chemiluminescence).tif]

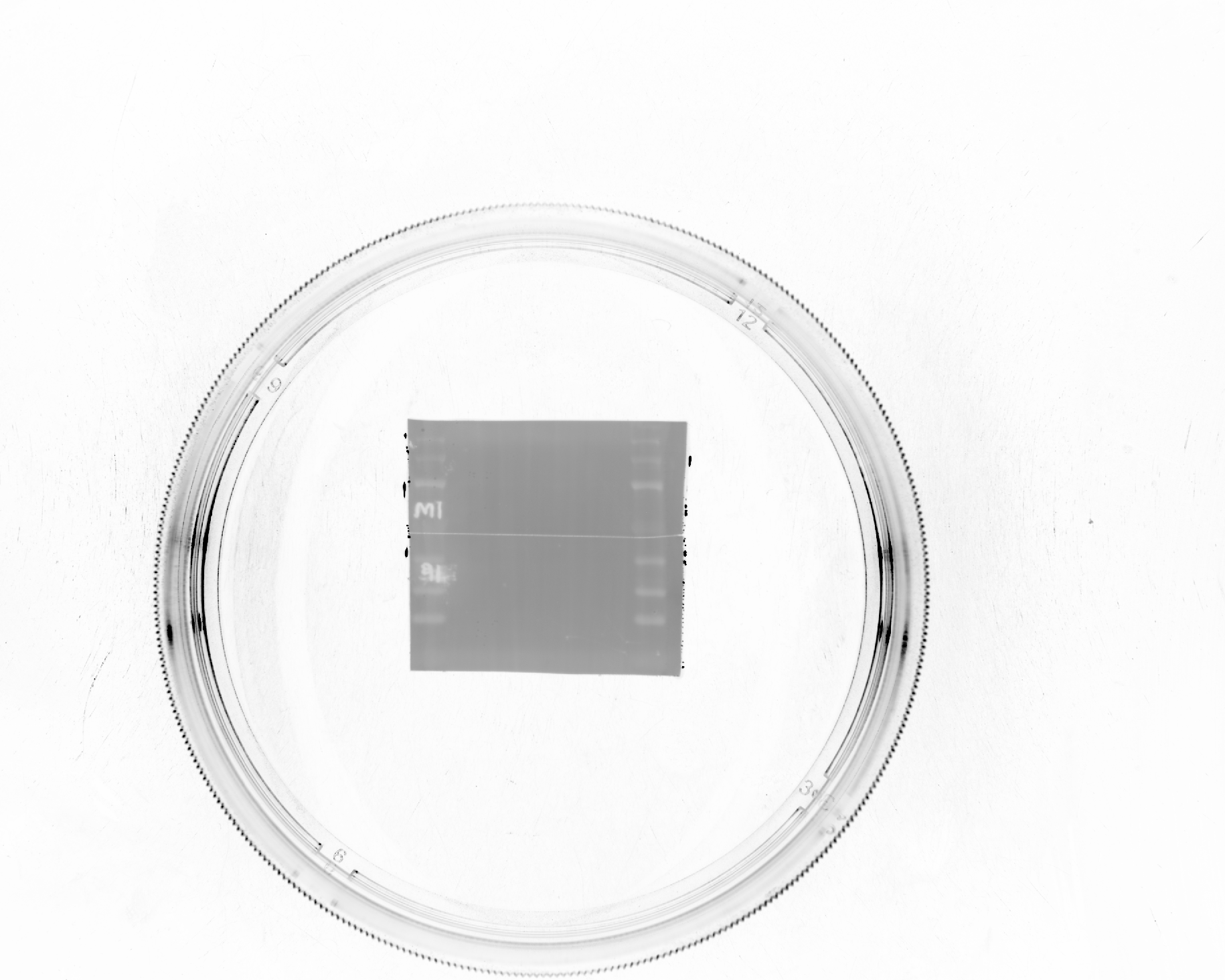

Supplement: Supplementary file 6 [file DataSheet10.zip › 3MMP9/3MMP9&beta-(Colorimetric).tif]

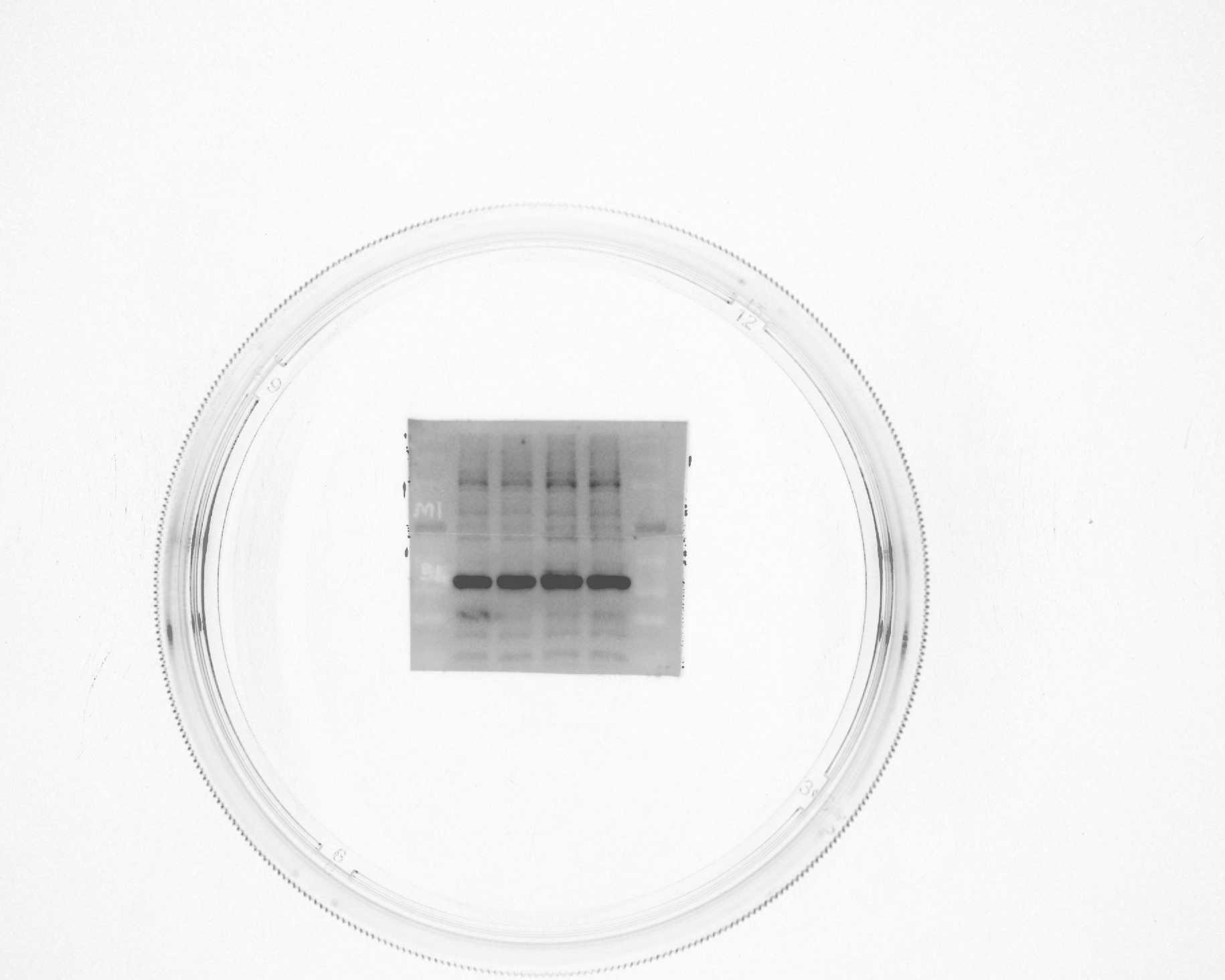

Supplement: Supplementary file 6 [file DataSheet10.zip › 3MMP9/3MMP9&beta-(复合).tif]

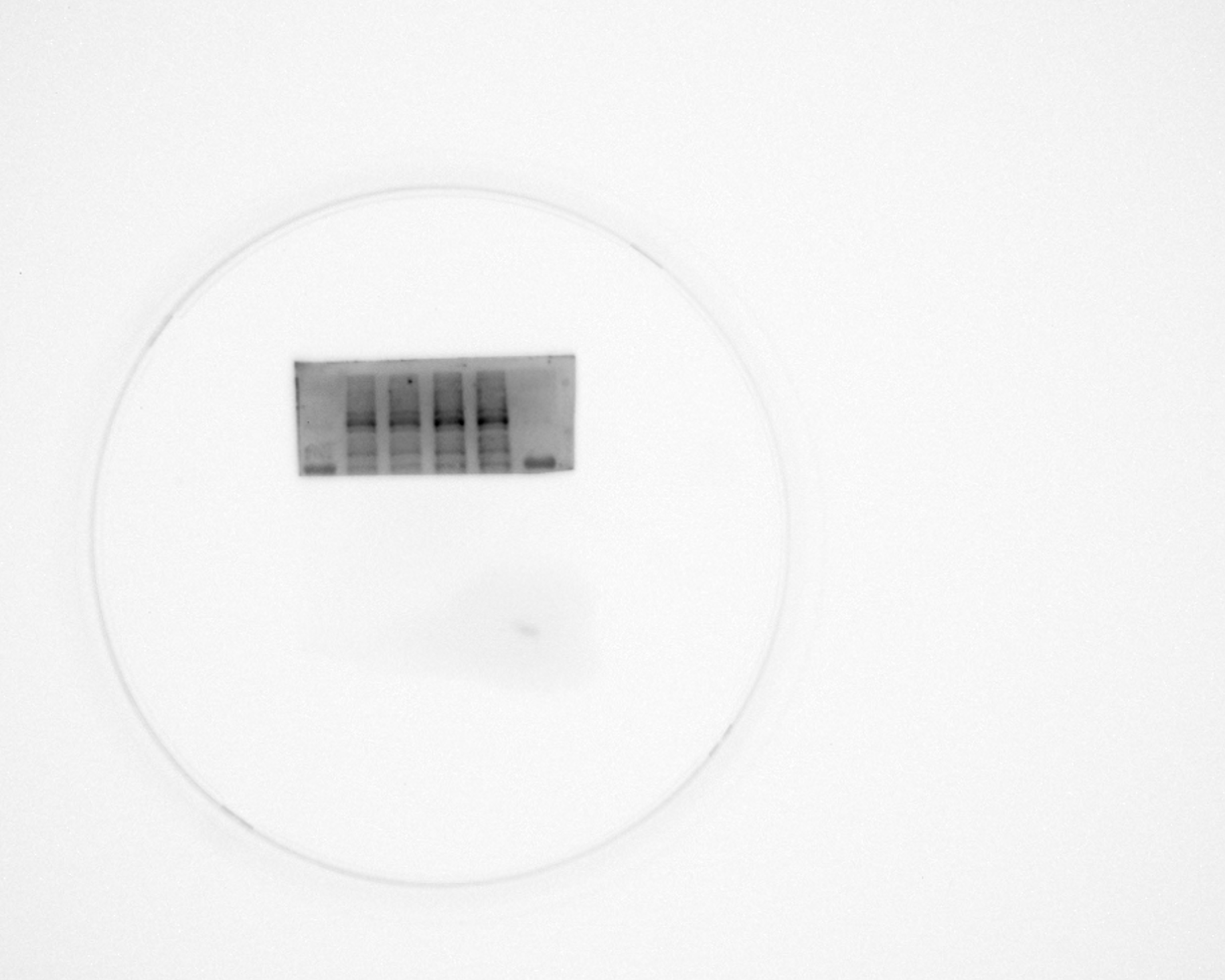

Supplement: Supplementary file 6 [file DataSheet10.zip › 3MMP9/3MMP9(Chemiluminescence).tif]

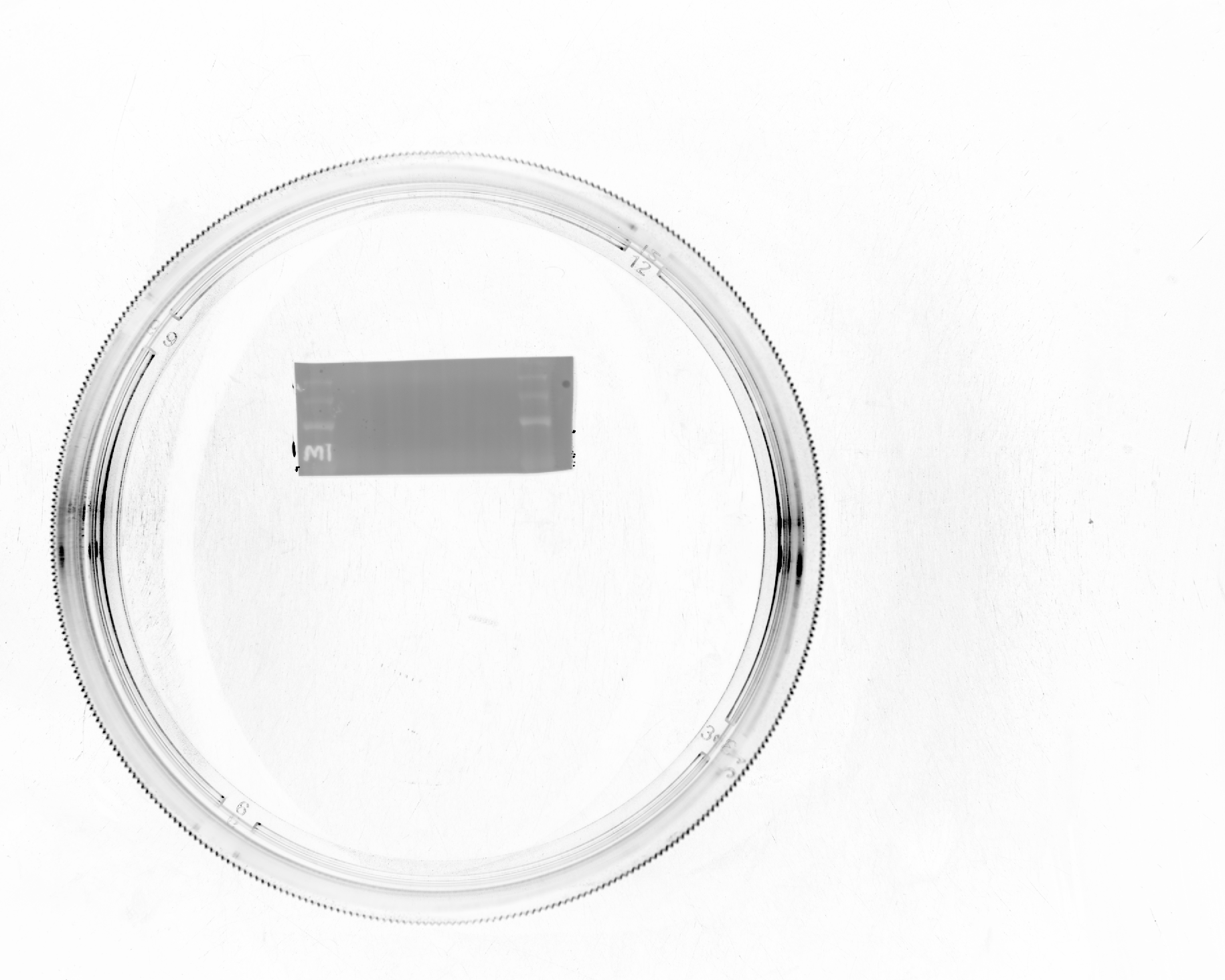

Supplement: Supplementary file 6 [file DataSheet10.zip › 3MMP9/3MMP9-(Colorimetric).tif]

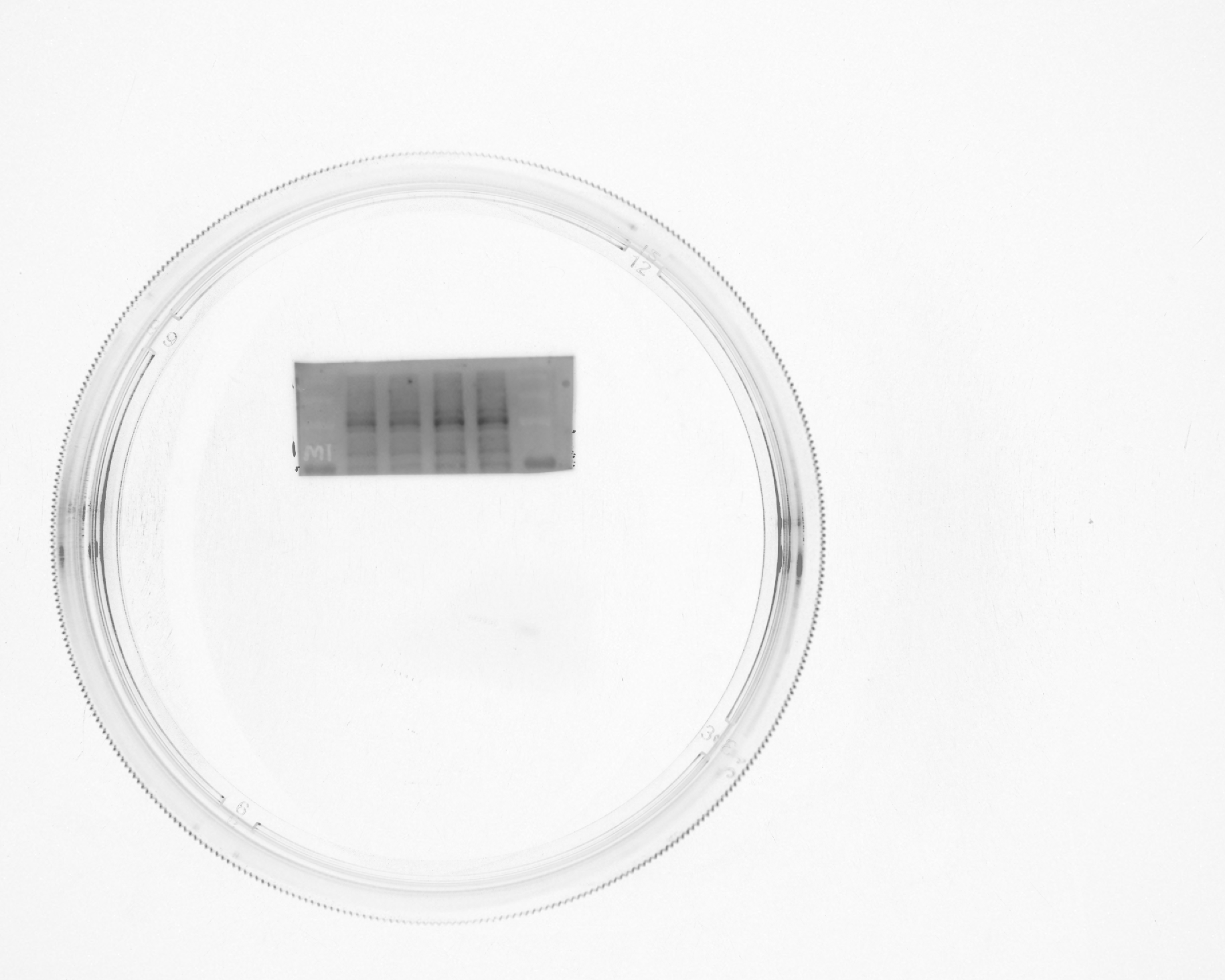

Supplement: Supplementary file 6 [file DataSheet10.zip › 3MMP9/3MMP9-(复合).tif]

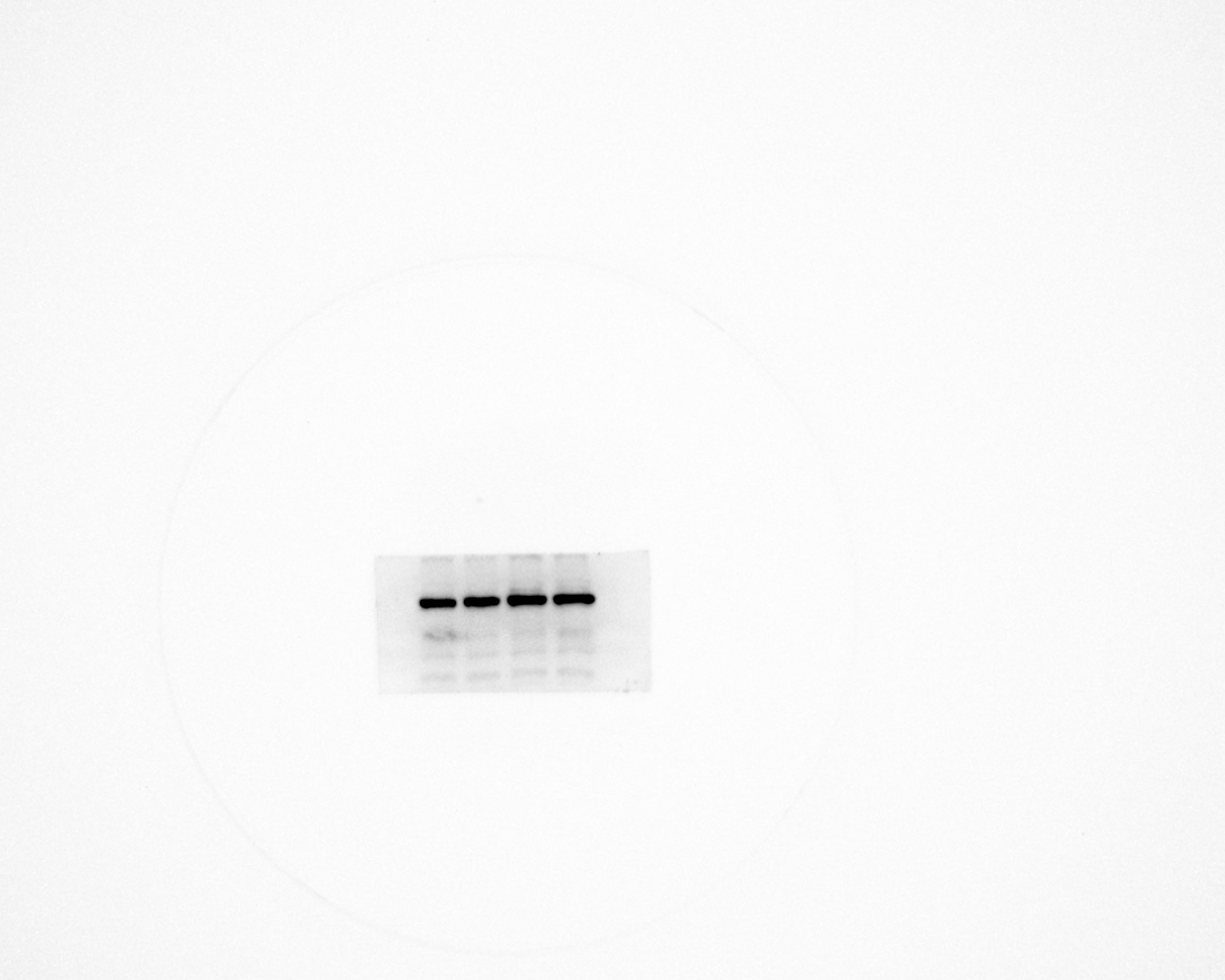

Supplement: Supplementary file 6 [file DataSheet10.zip › 3MMP9/3MMP9-beta(Chemiluminescence).tif]

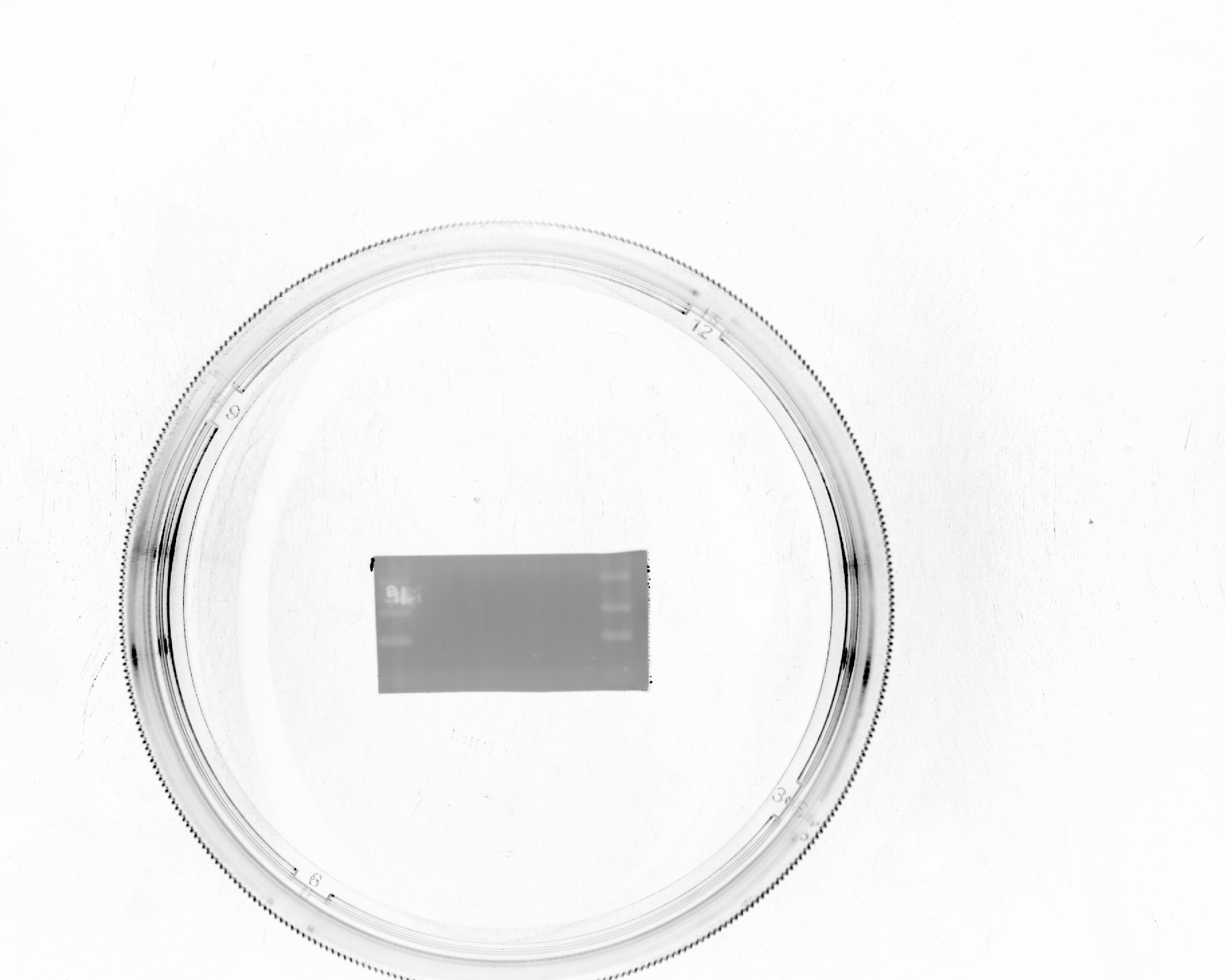

Supplement: Supplementary file 6 [file DataSheet10.zip › 3MMP9/3MMP9-beta-(Colorimetric).tif]

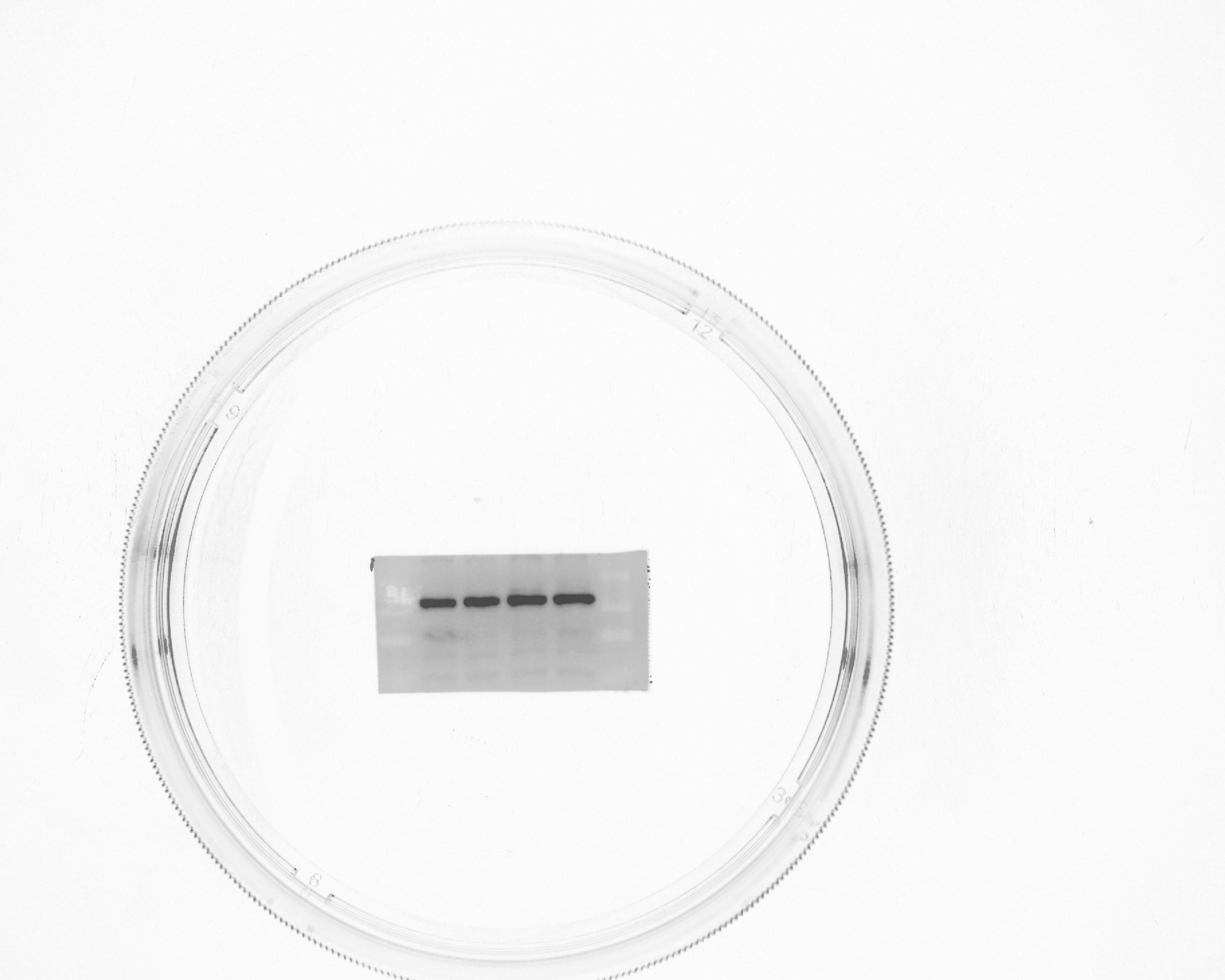

Supplement: Supplementary file 6 [file DataSheet10.zip › 3MMP9/3MMP9-beta-(复合).tif]

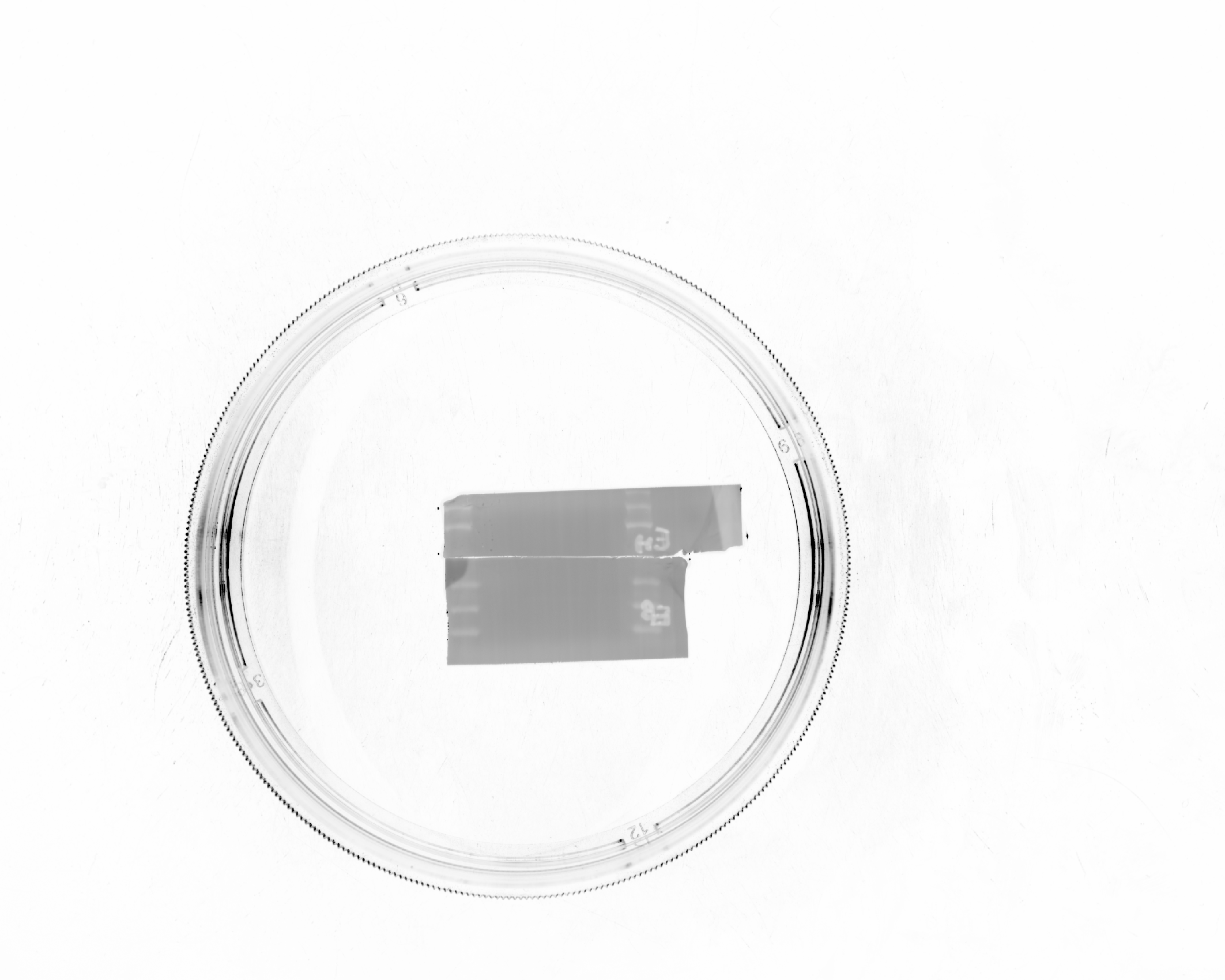

Supplement: Supplementary file 7 [file DataSheet6.zip › 2HIF1α/2HIF1α&1(Colorimetric).tif]

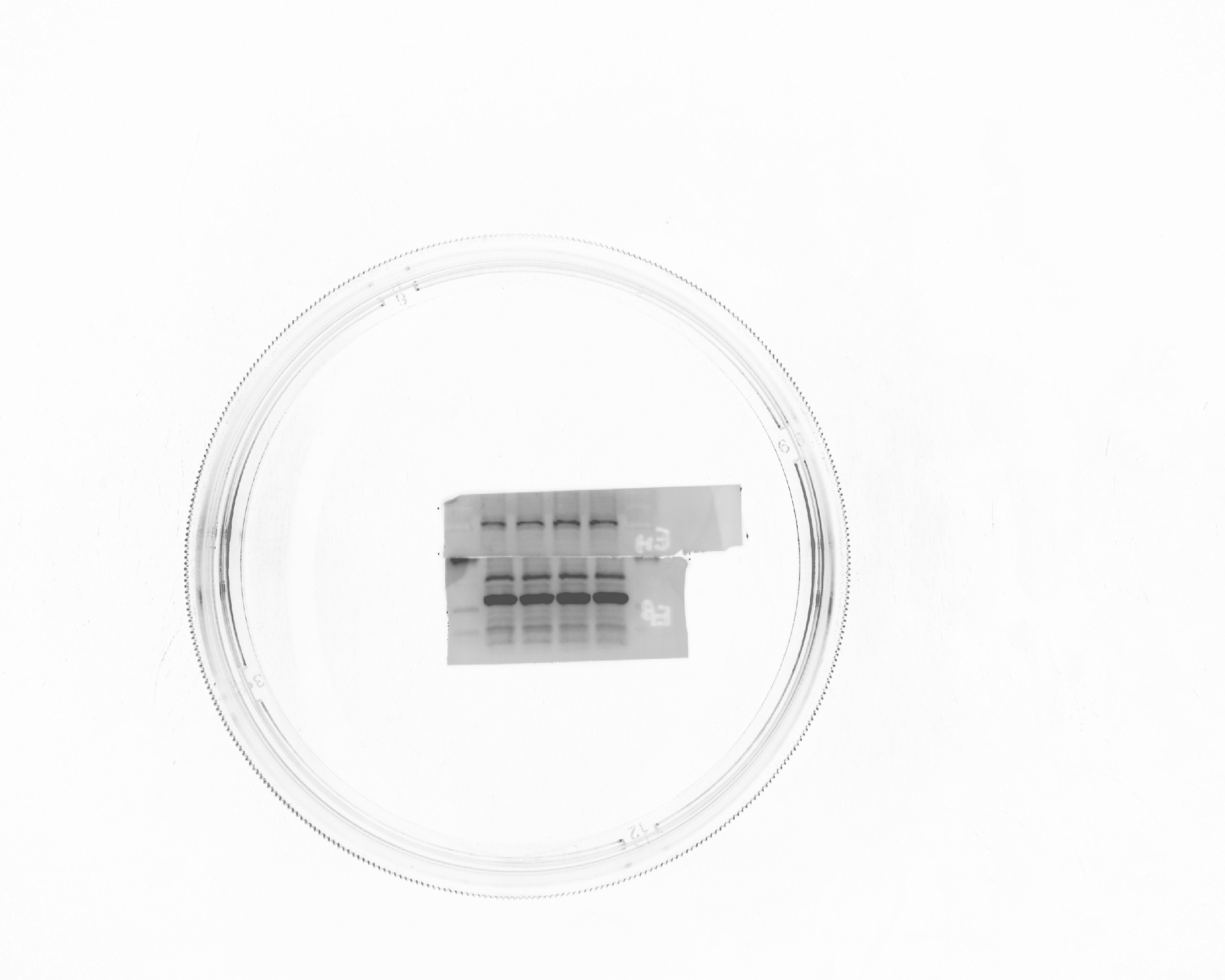

Supplement: Supplementary file 7 [file DataSheet6.zip › 2HIF1α/2HIF1α&1-(复合).tif]

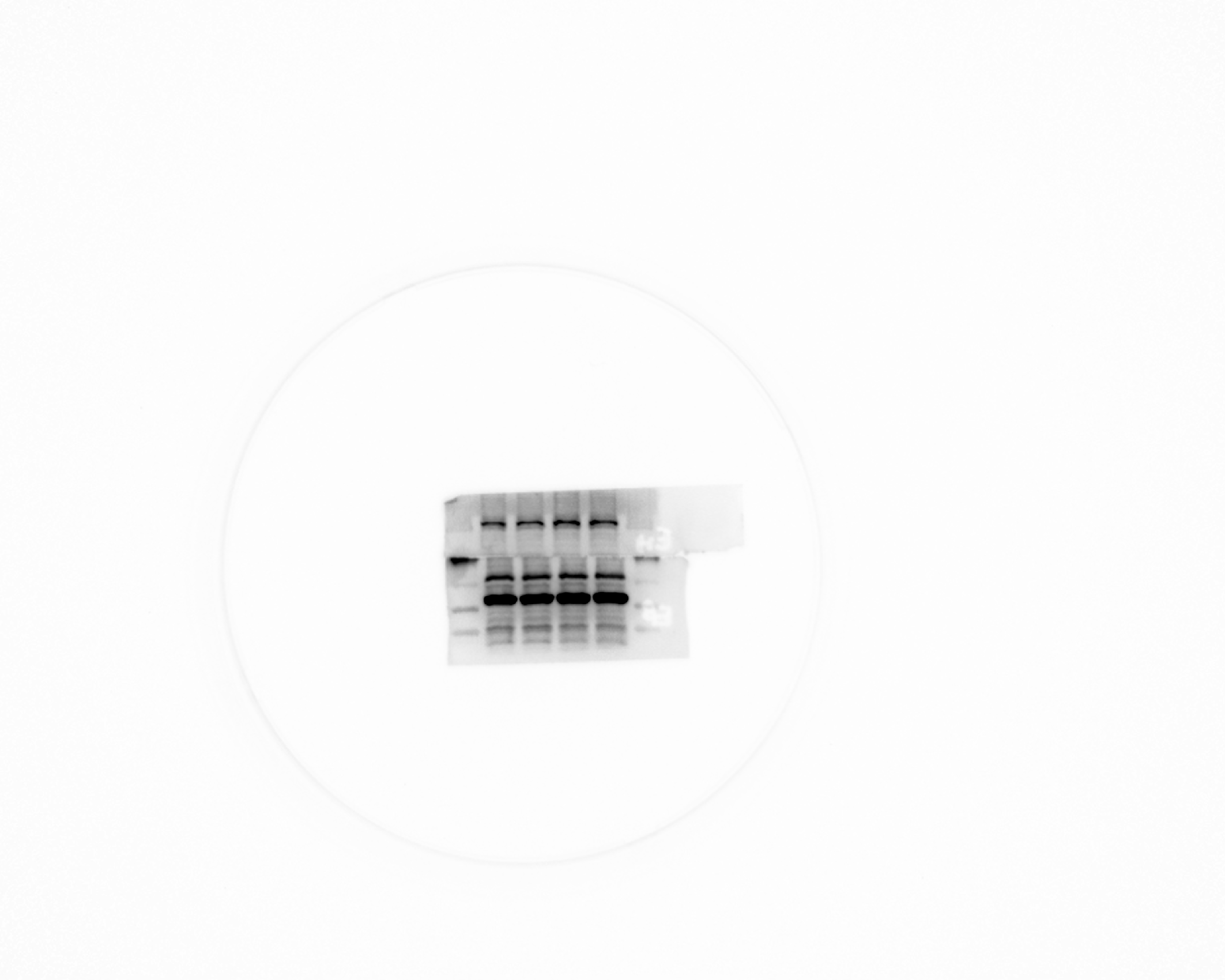

Supplement: Supplementary file 7 [file DataSheet6.zip › 2HIF1α/2HIF1α&beta(Chemiluminescence).tif]

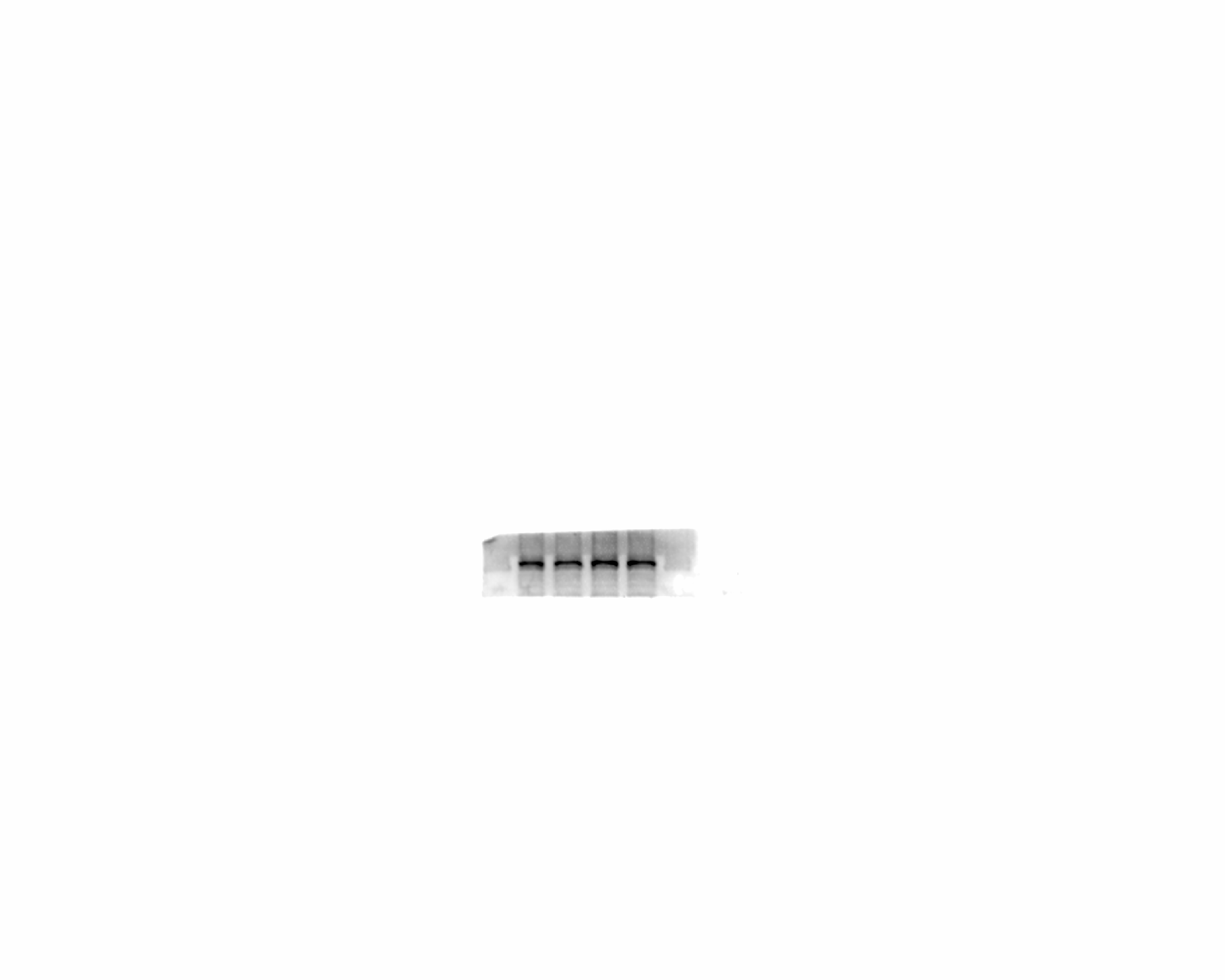

Supplement: Supplementary file 7 [file DataSheet6.zip › 2HIF1α/2HIF1α(Chemiluminescence).tif]

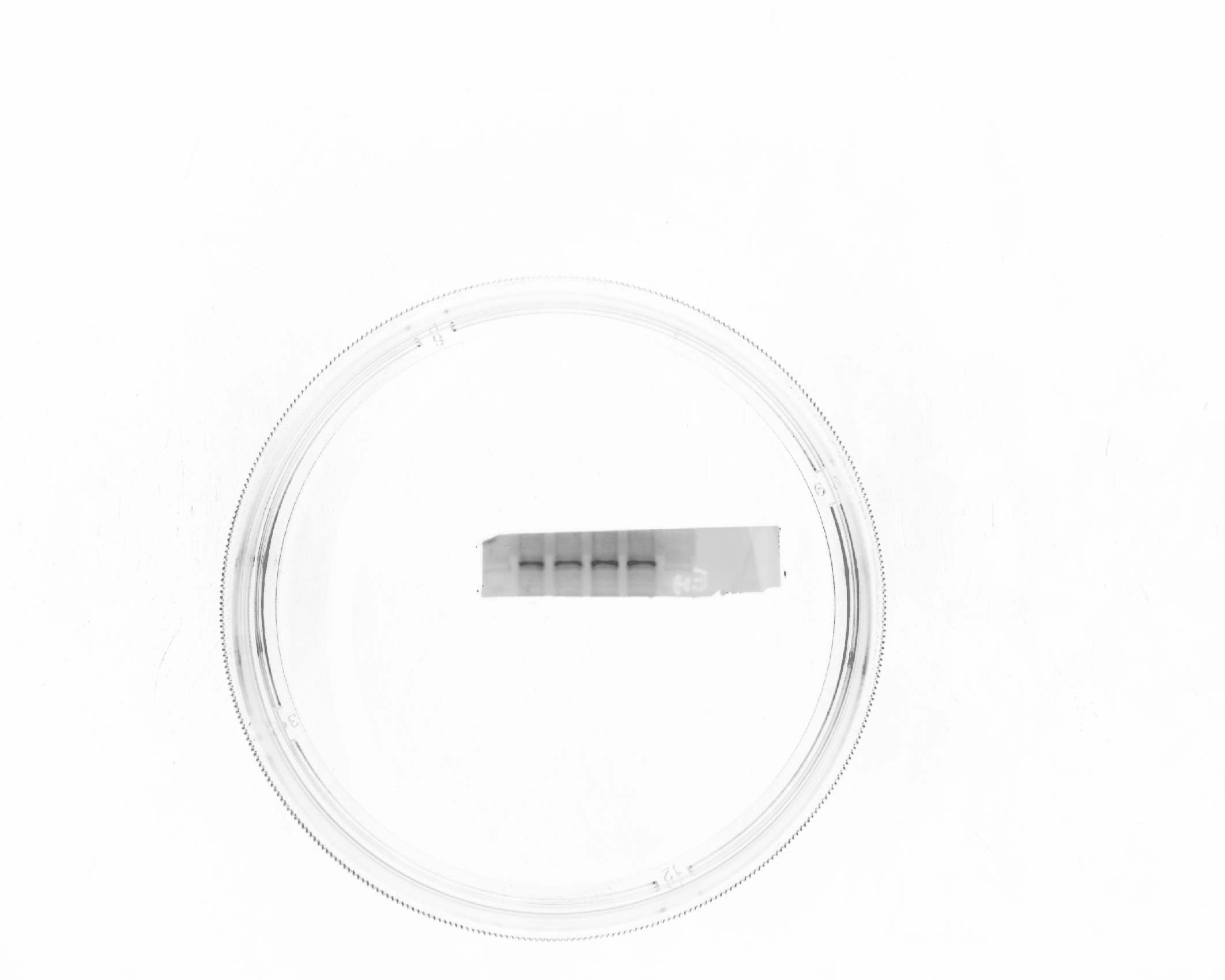

Supplement: Supplementary file 7 [file DataSheet6.zip › 2HIF1α/2HIF1α-(复合).tif]

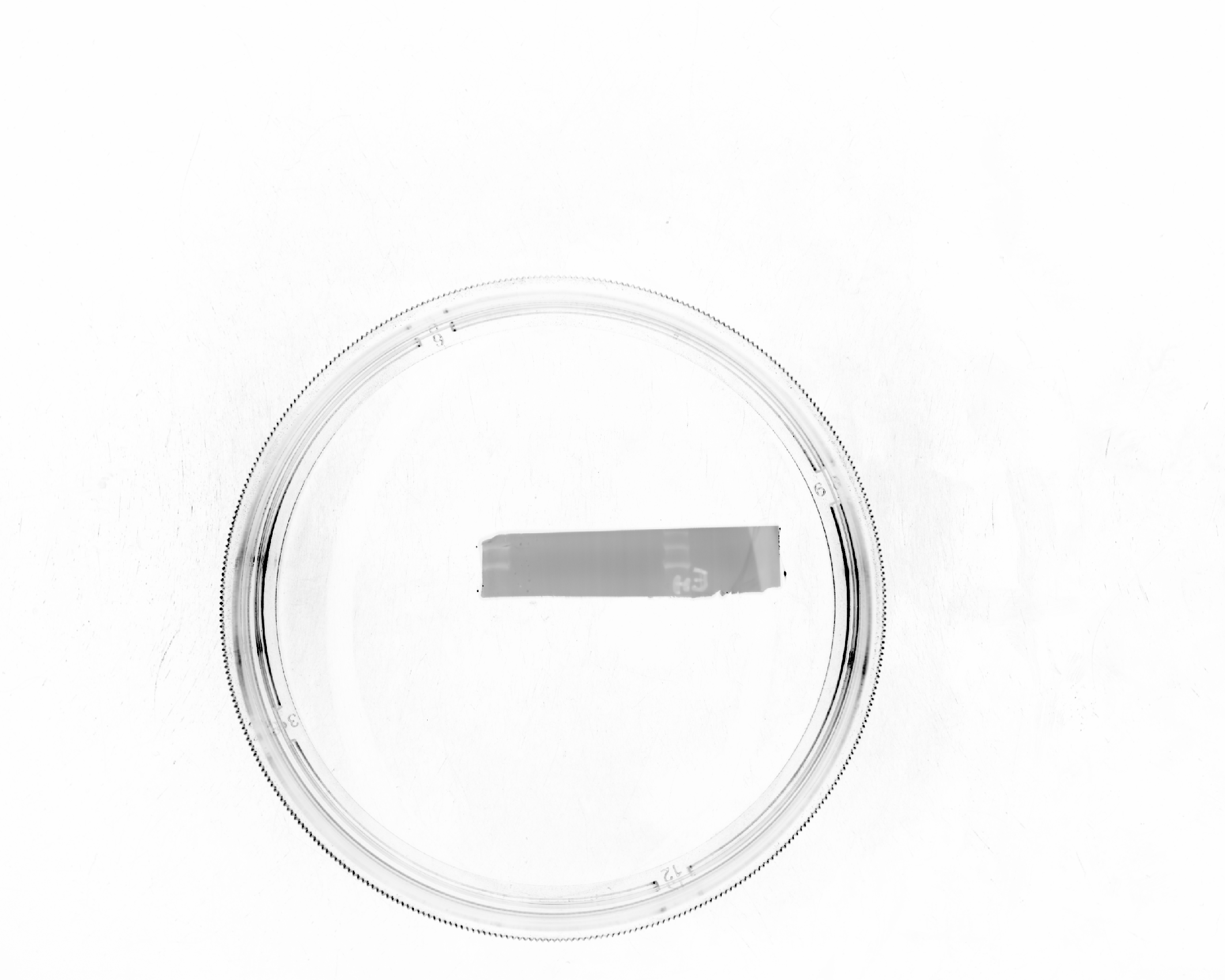

Supplement: Supplementary file 7 [file DataSheet6.zip › 2HIF1α/2HIF1α--(Colorimetric).tif]

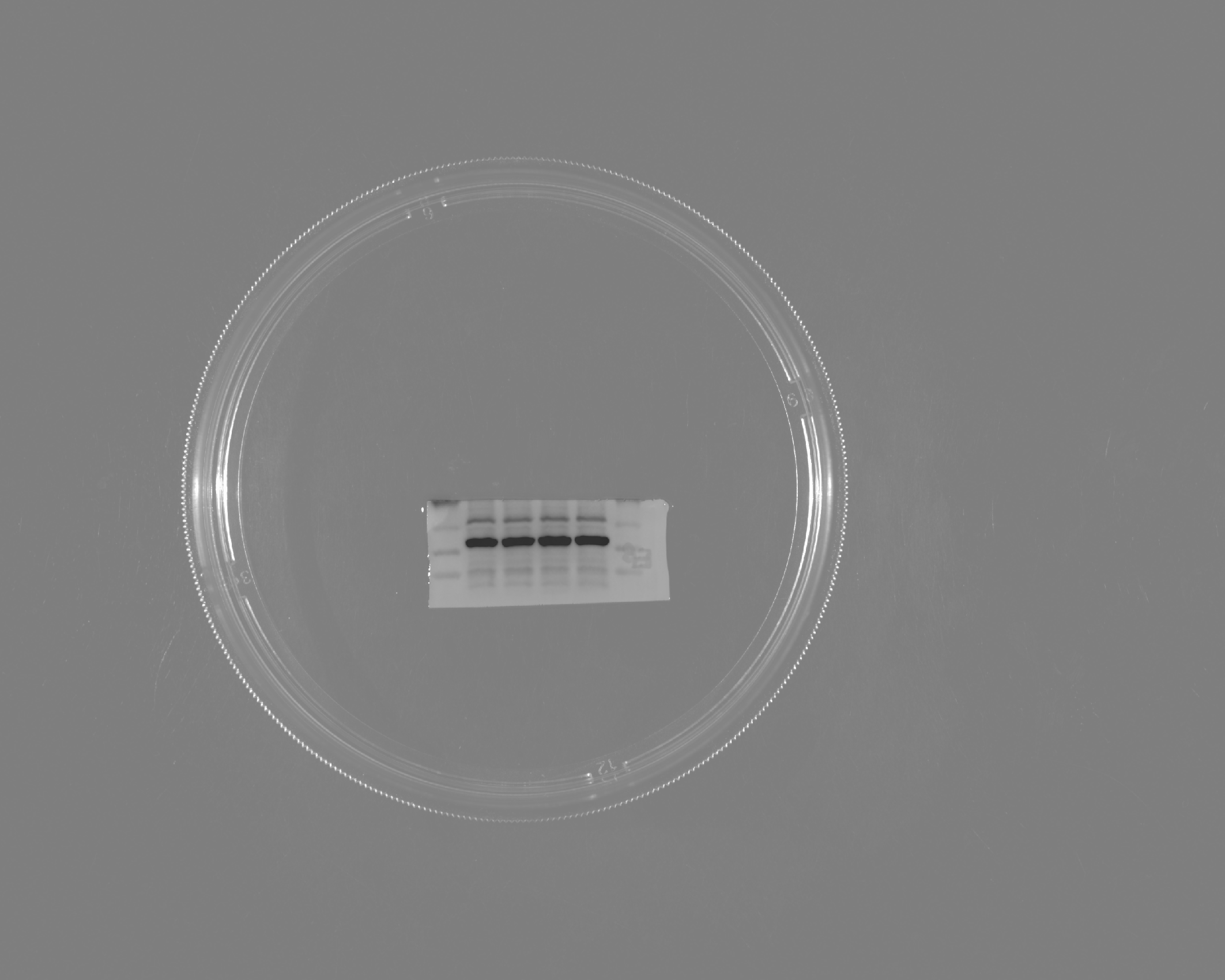

Supplement: Supplementary file 7 [file DataSheet6.zip › 2HIF1α/2HIF1α--beta(复合).tif]

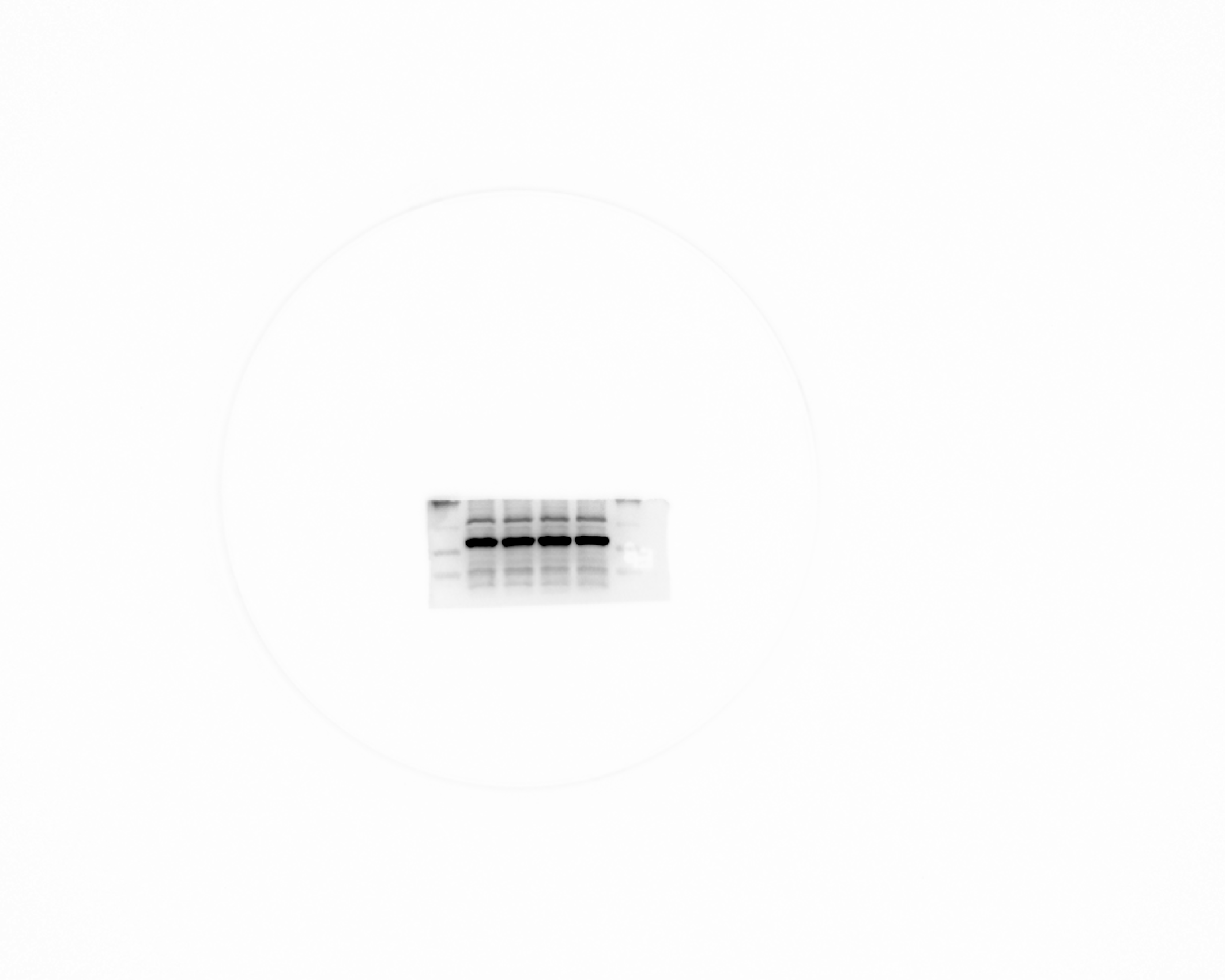

Supplement: Supplementary file 7 [file DataSheet6.zip › 2HIF1α/2HIF1α-beta(Chemiluminescence).tif]

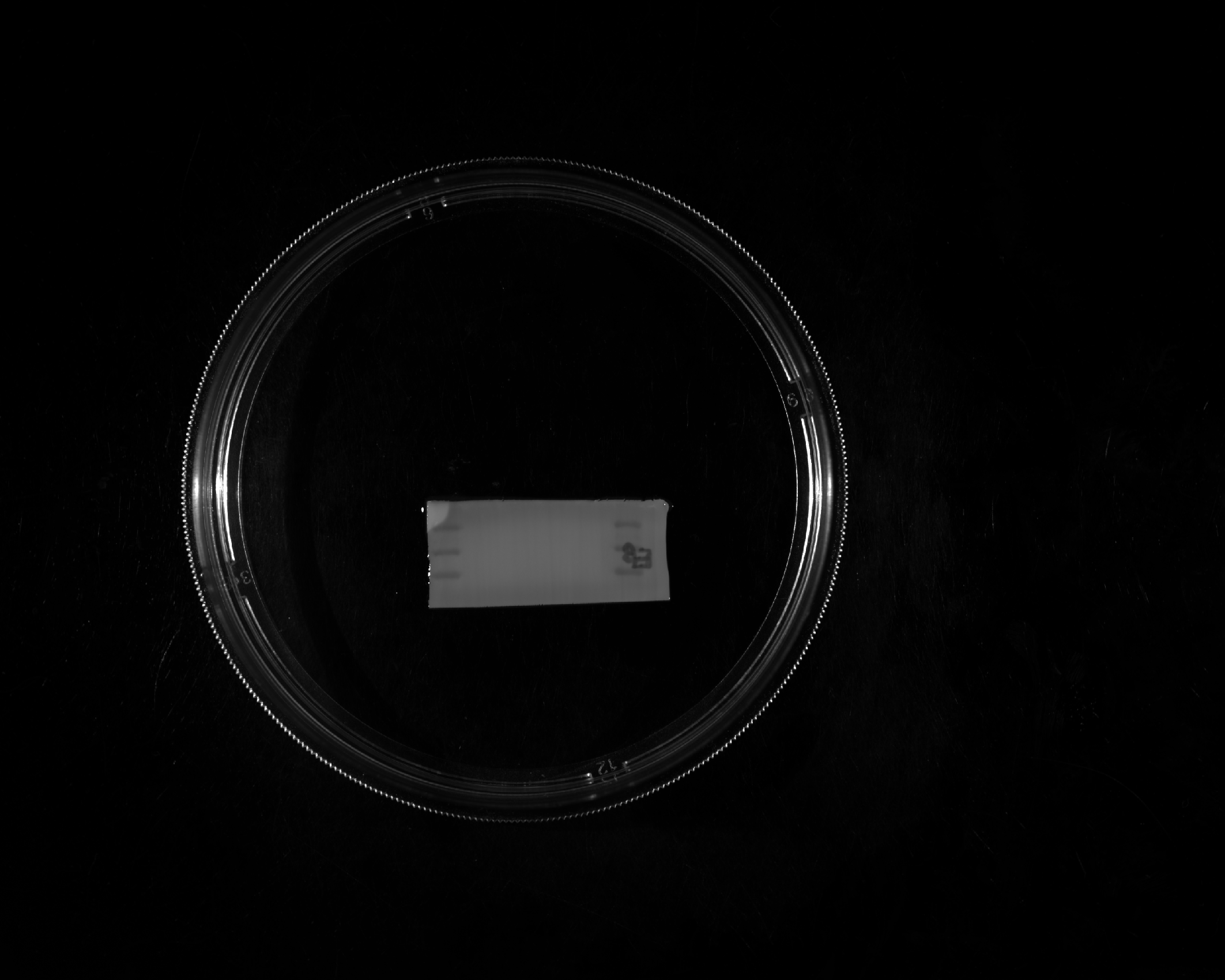

Supplement: Supplementary file 7 [file DataSheet6.zip › 2HIF1α/2HIF1α-beta(Colorimetric).tif]

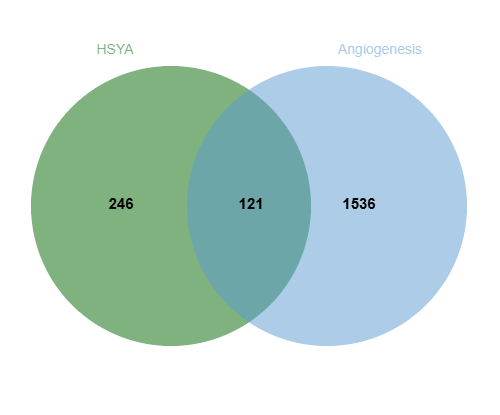

Supplement: Supplementary file 8 [file DataSheet2.zip › Figure2-original data/Figure2A/venn.png]

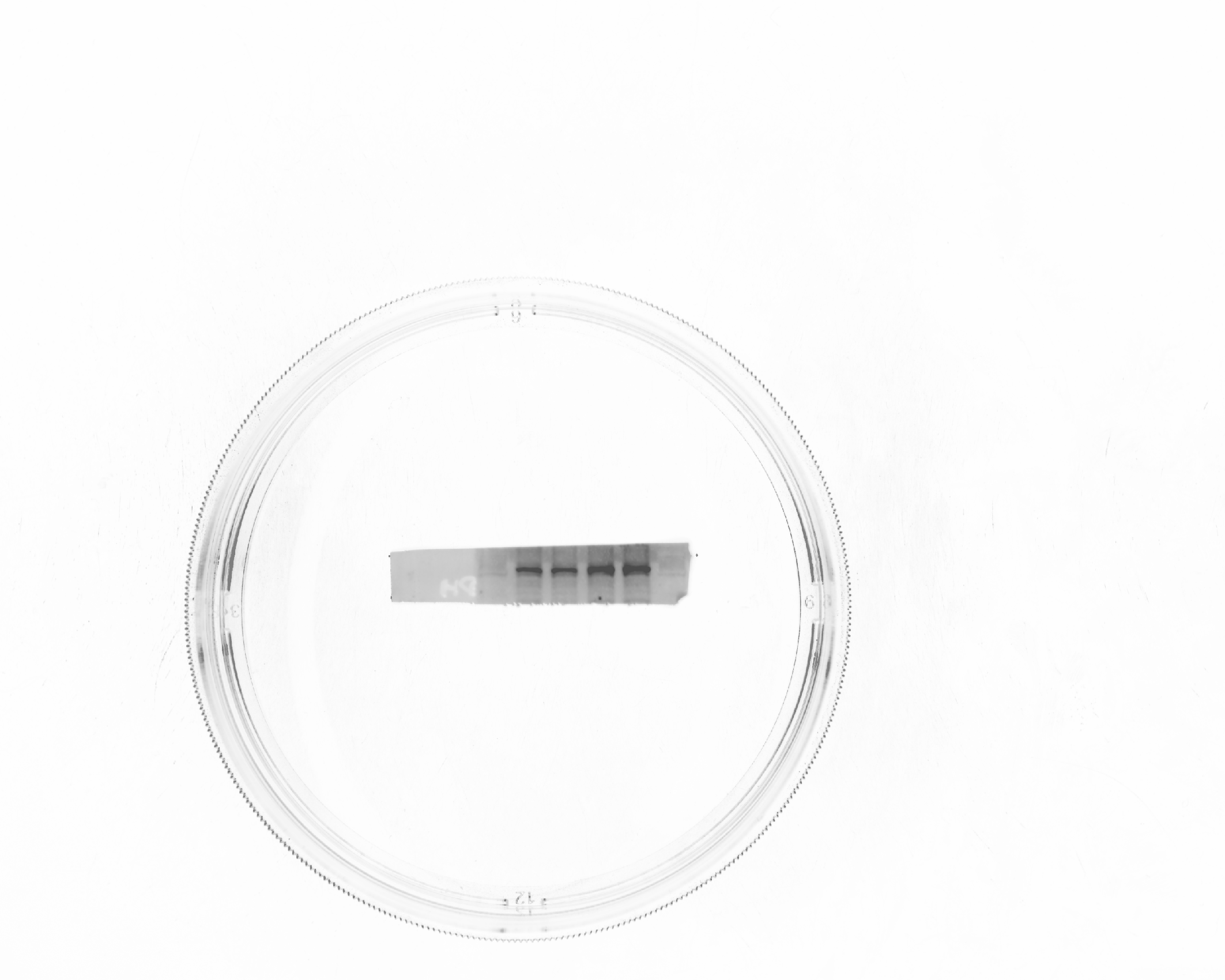

Supplement: Supplementary file 9 [file DataSheet5.zip › 1HIF1α/1-HIF1α(复合).tif]

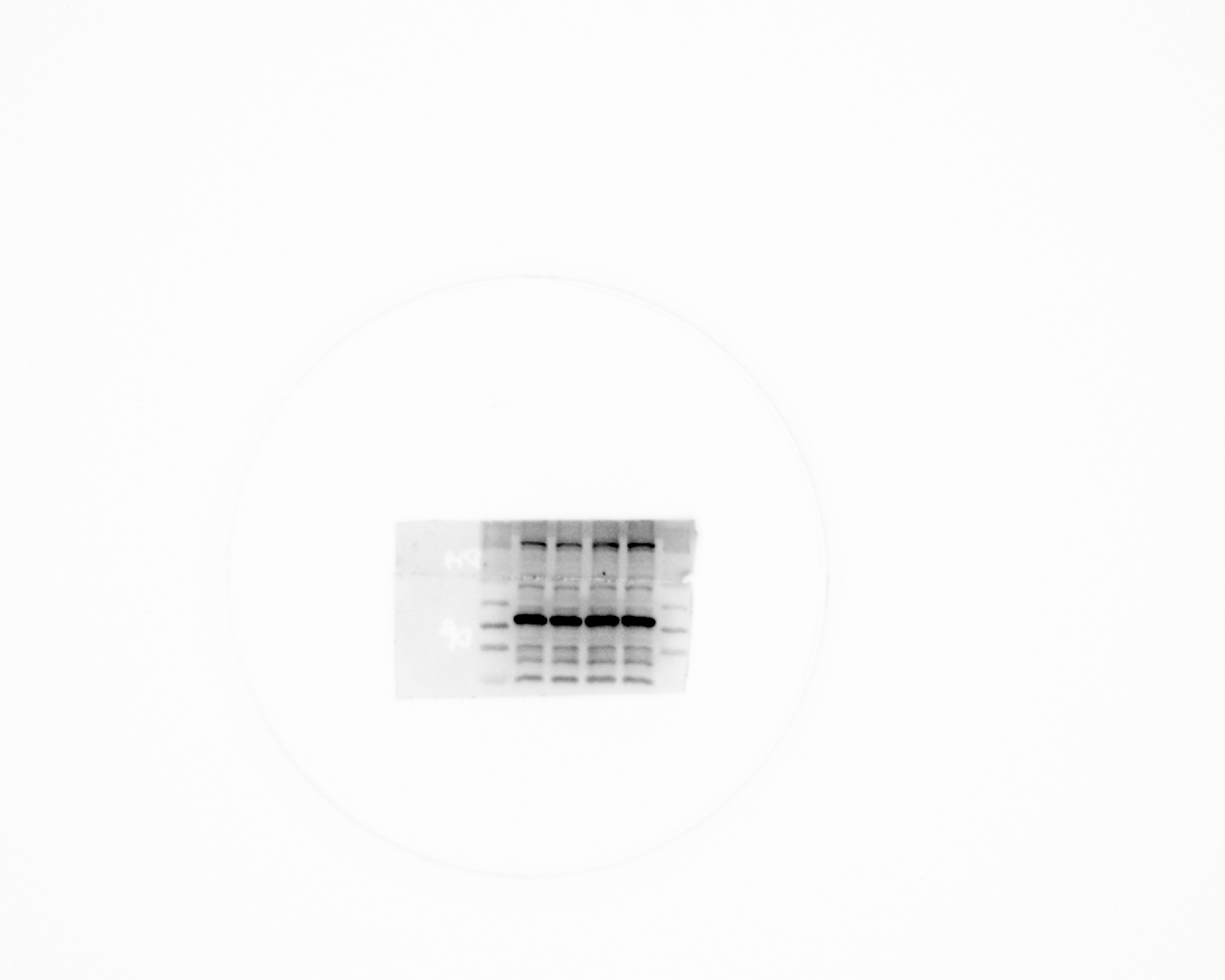

Supplement: Supplementary file 9 [file DataSheet5.zip › 1HIF1α/1HIF1α&beta(Chemiluminescence).tif]

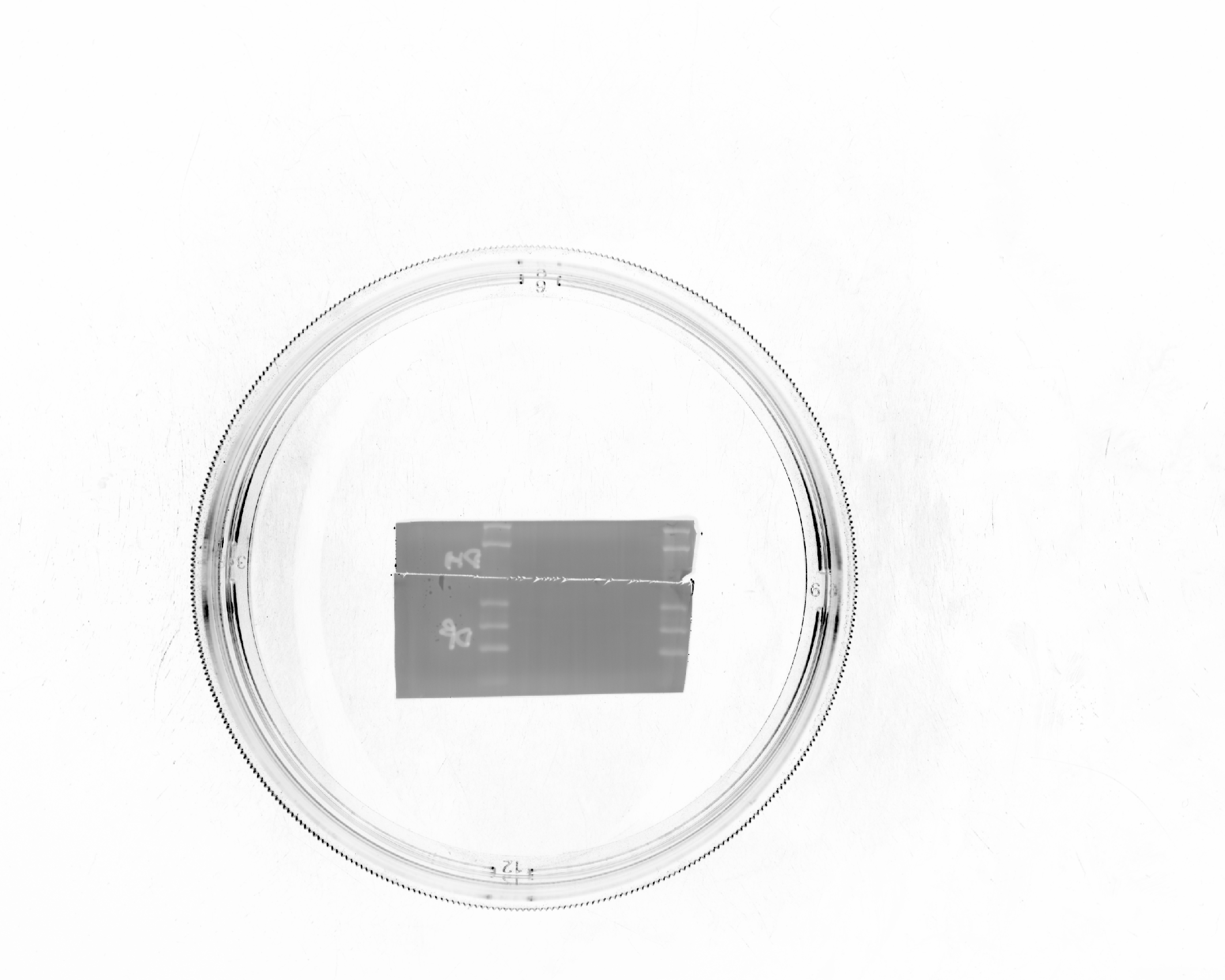

Supplement: Supplementary file 9 [file DataSheet5.zip › 1HIF1α/1HIF1α&beta(Colorimetric).tif]

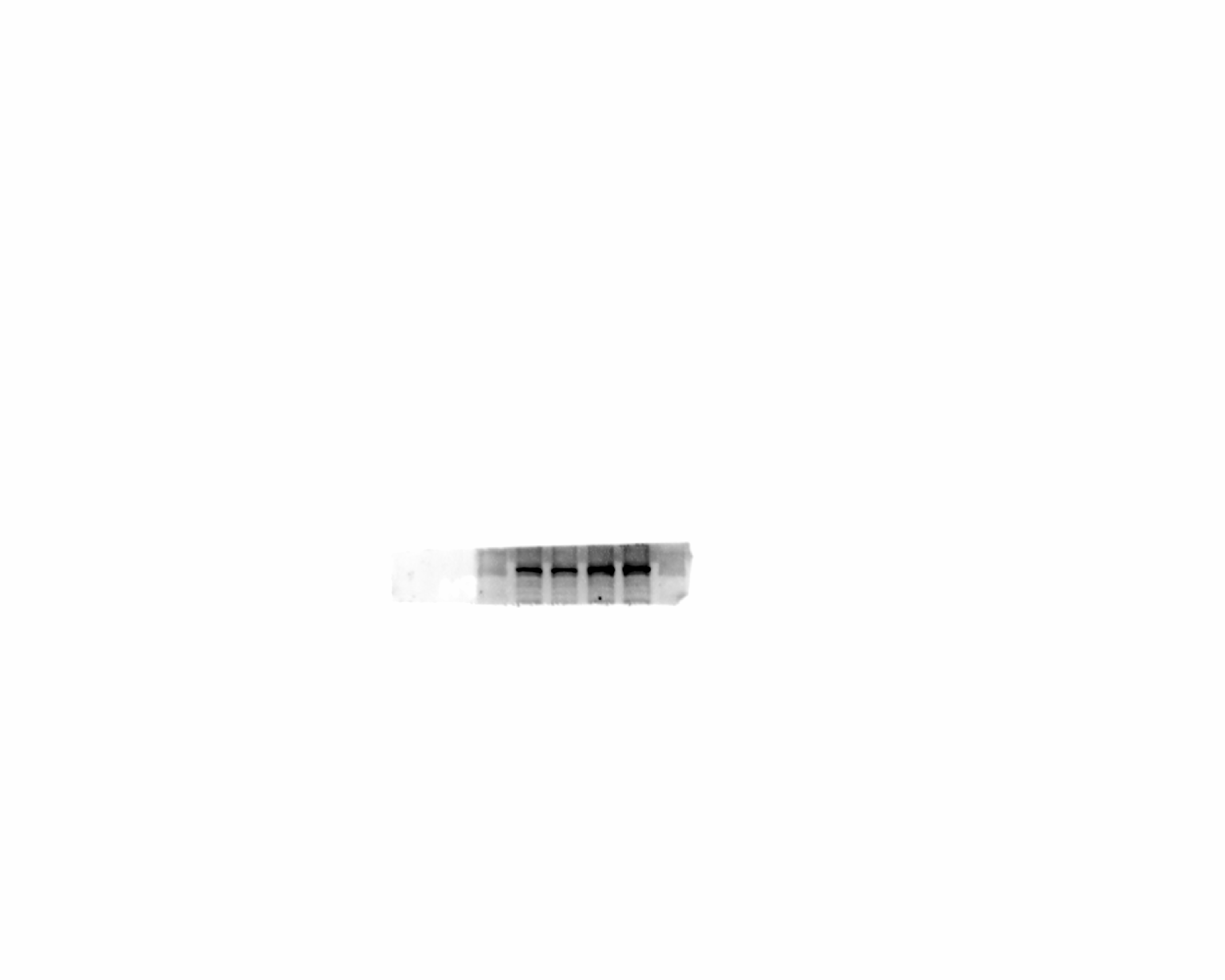

Supplement: Supplementary file 9 [file DataSheet5.zip › 1HIF1α/1HIF1α(Chemiluminescence).tif]

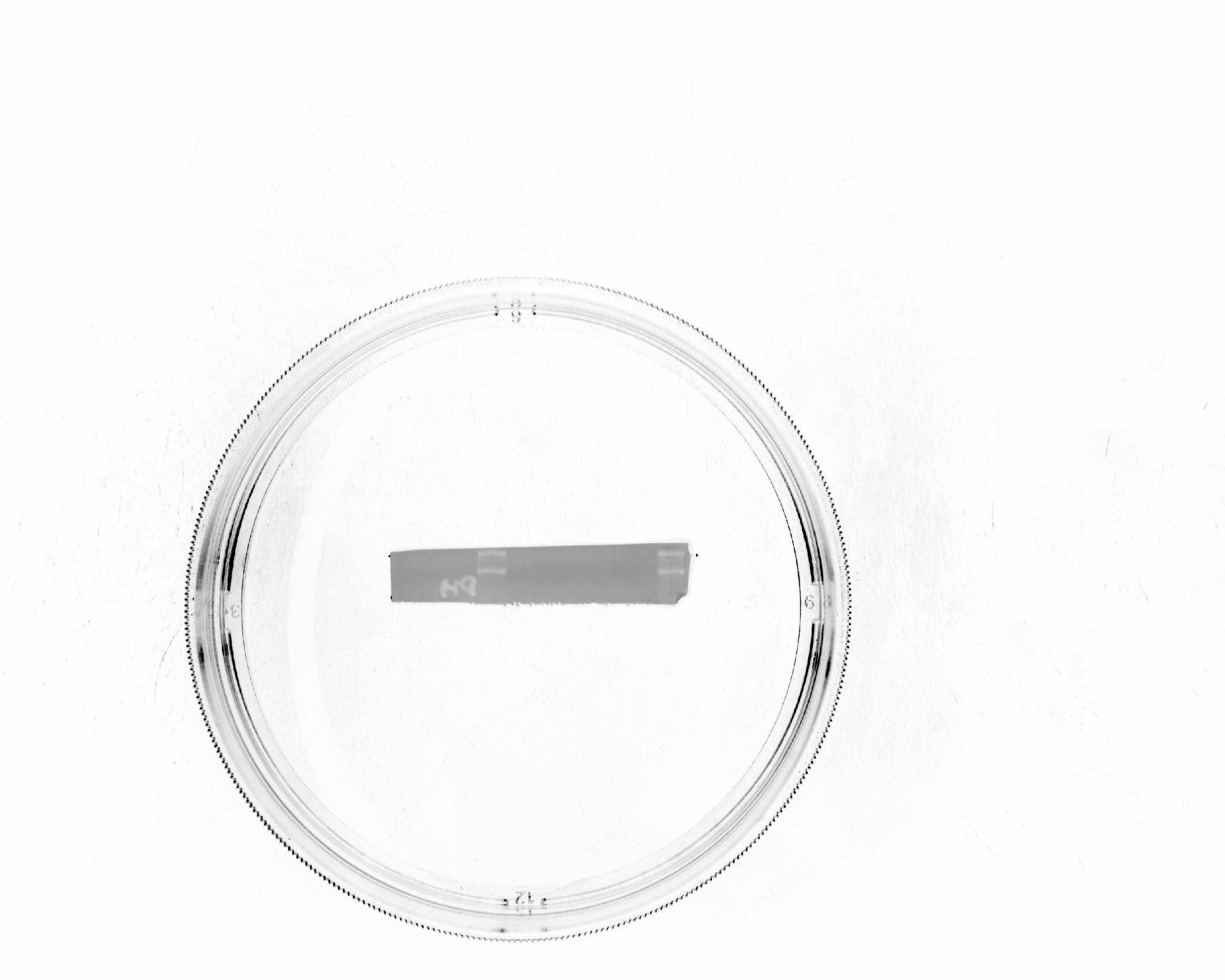

Supplement: Supplementary file 9 [file DataSheet5.zip › 1HIF1α/1HIF1α(Colorimetric).tif]

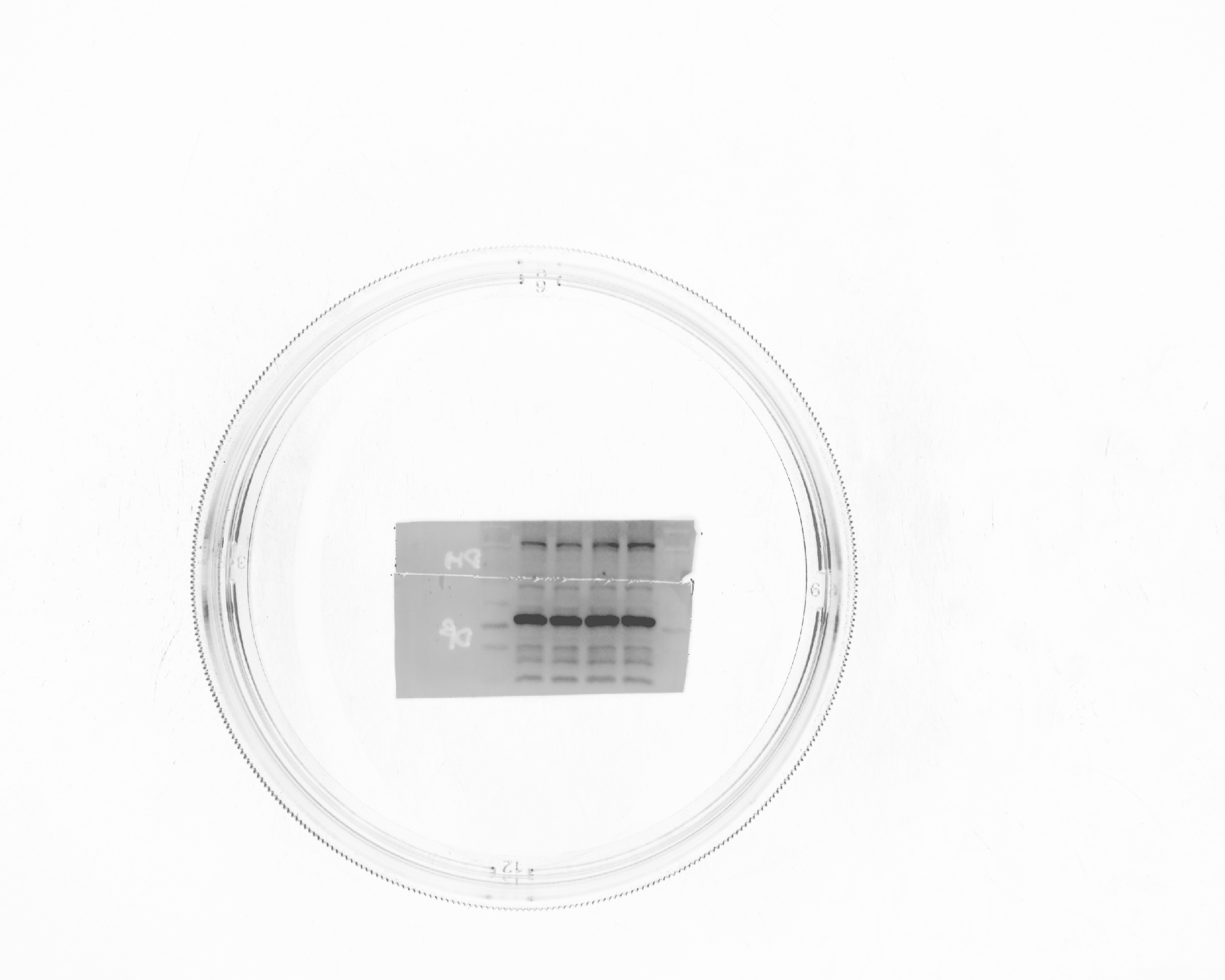

Supplement: Supplementary file 9 [file DataSheet5.zip › 1HIF1α/1HIF1α(复合).tif]

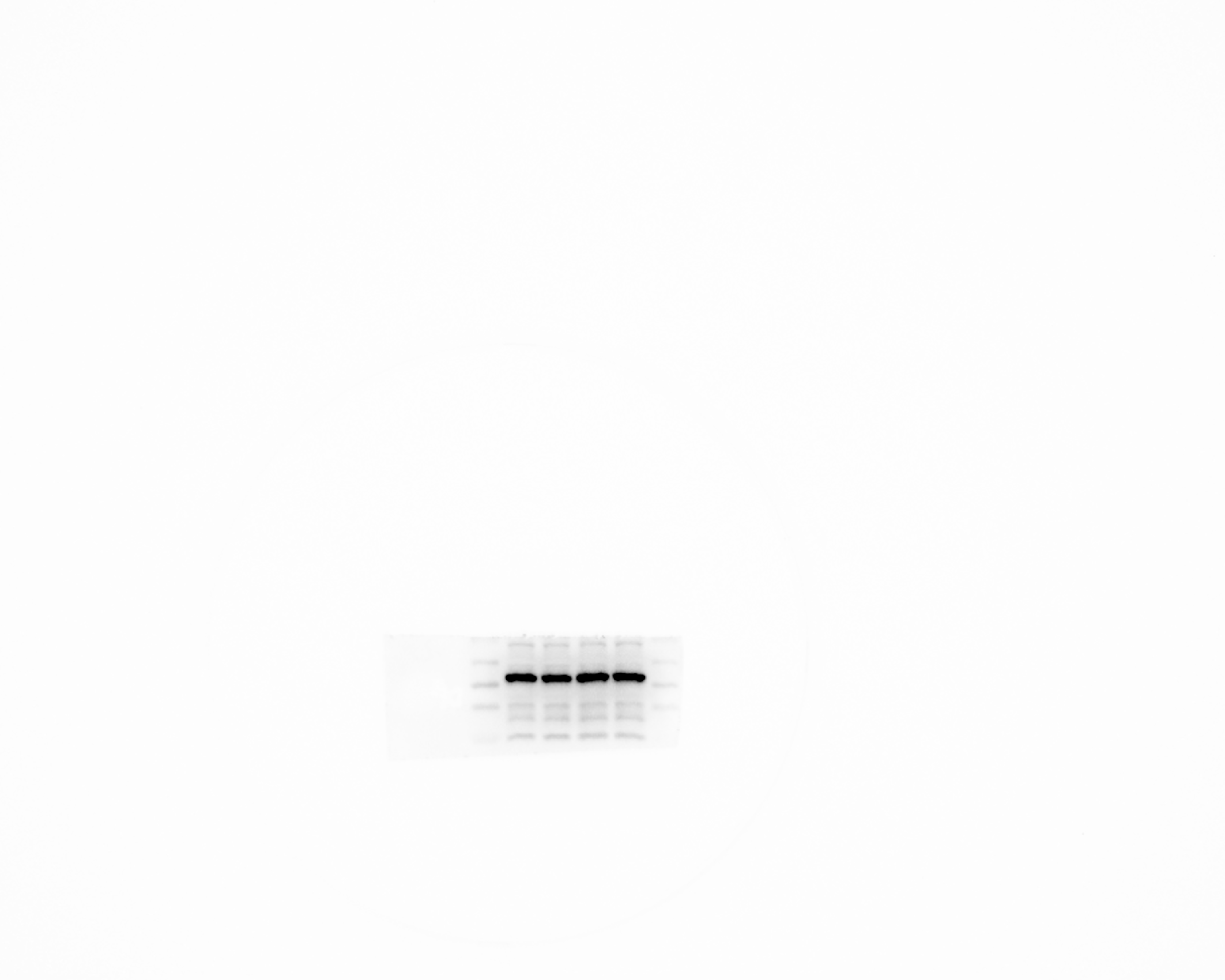

Supplement: Supplementary file 9 [file DataSheet5.zip › 1HIF1α/1HIF1α-beta(Chemiluminescence).tif]

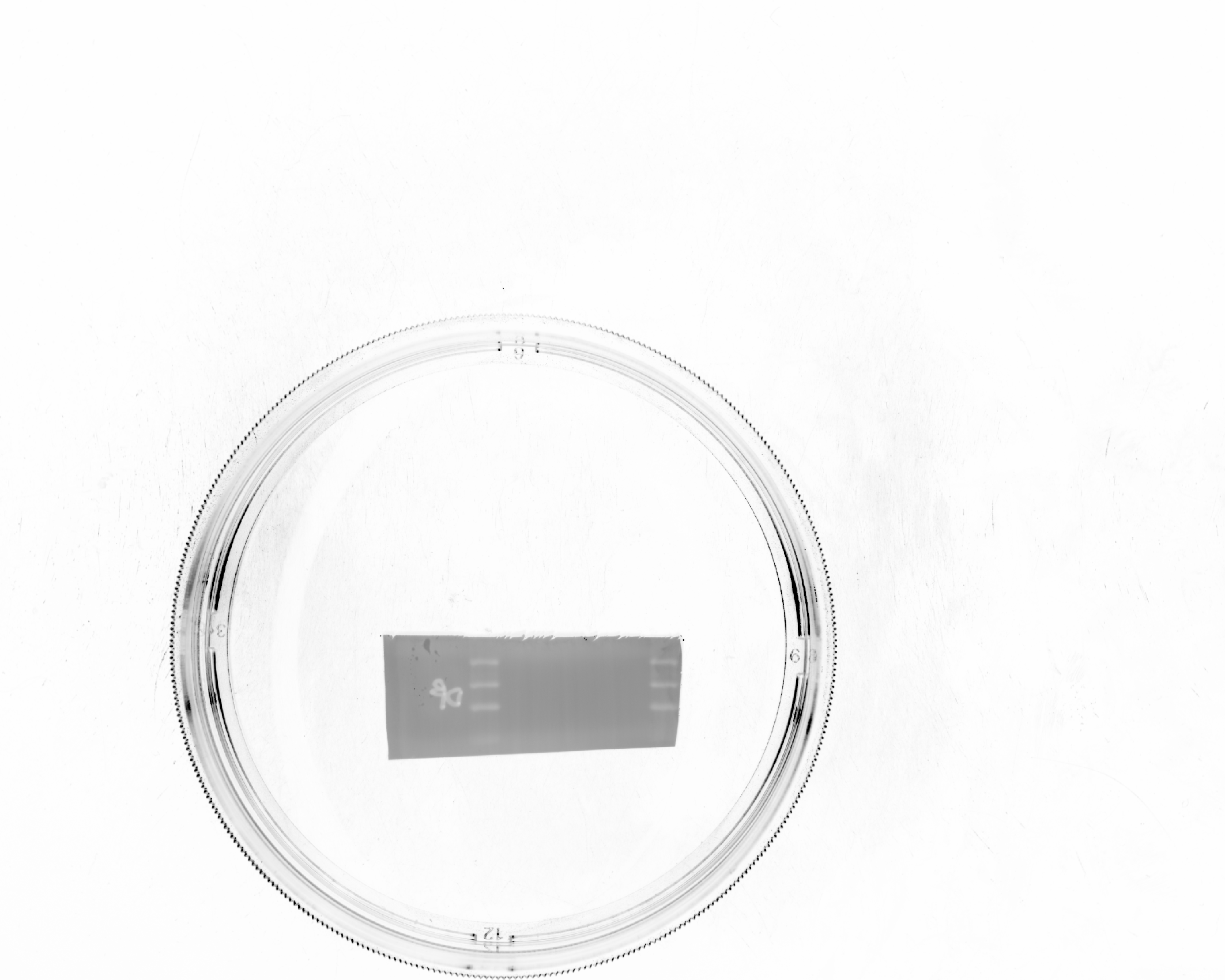

Supplement: Supplementary file 9 [file DataSheet5.zip › 1HIF1α/1HIF1α-beta(Colorimetric).tif]

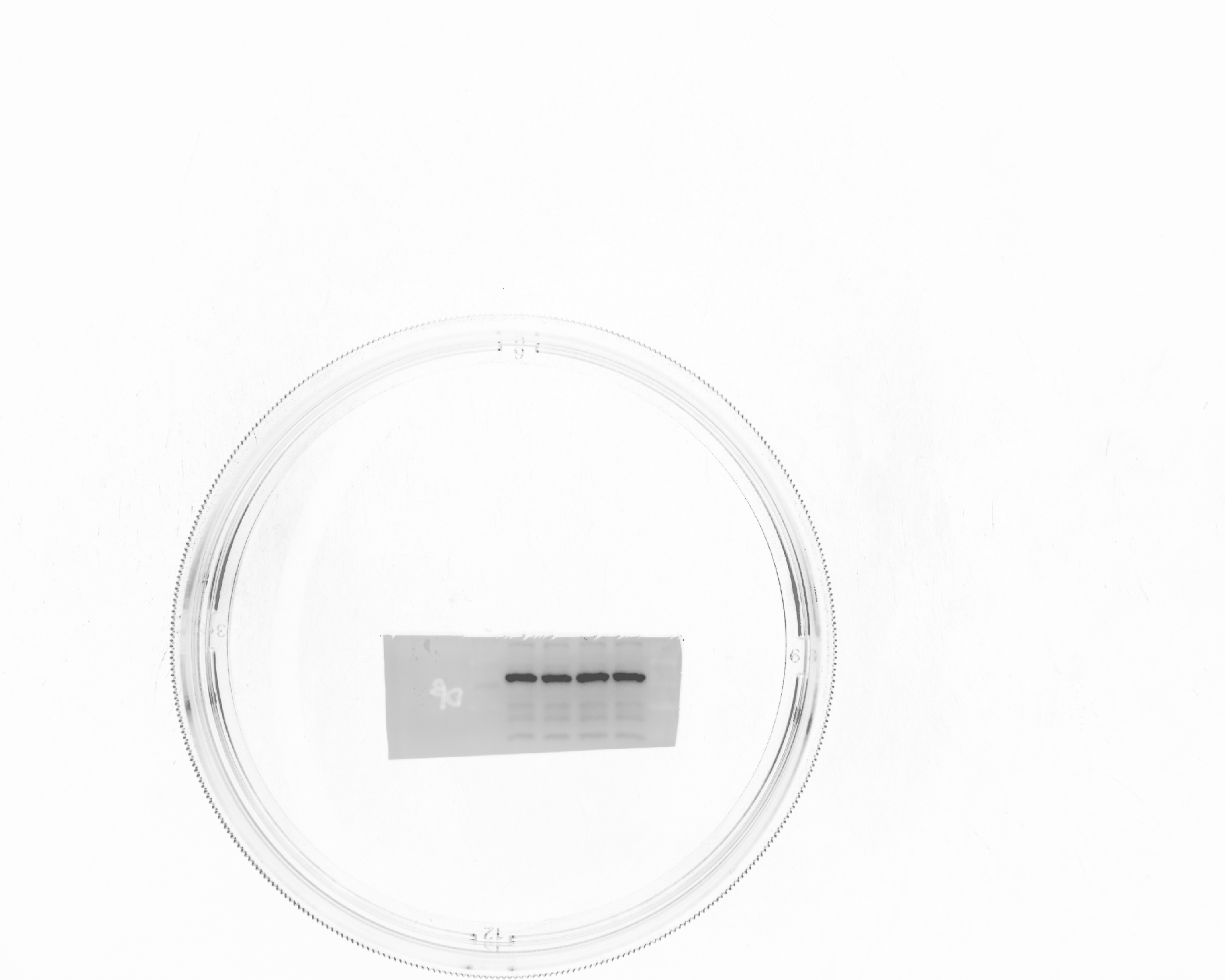

Supplement: Supplementary file 9 [file DataSheet5.zip › 1HIF1α/1HIF1α-beta(复合).tif]

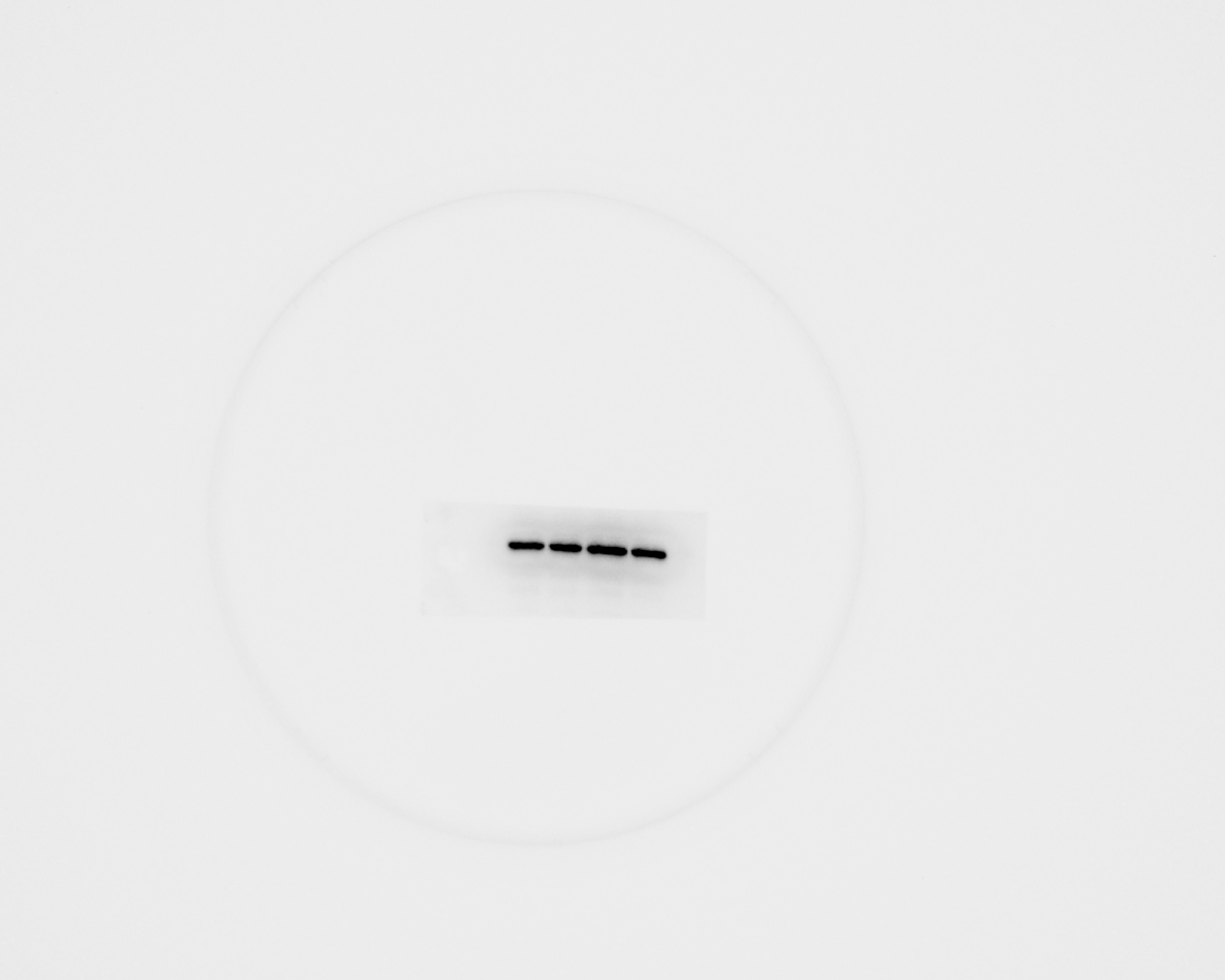

Supplement: Supplementary file 10 [file DataSheet7.zip › 3HIF1α/3-beta(Chemiluminescence).tif]

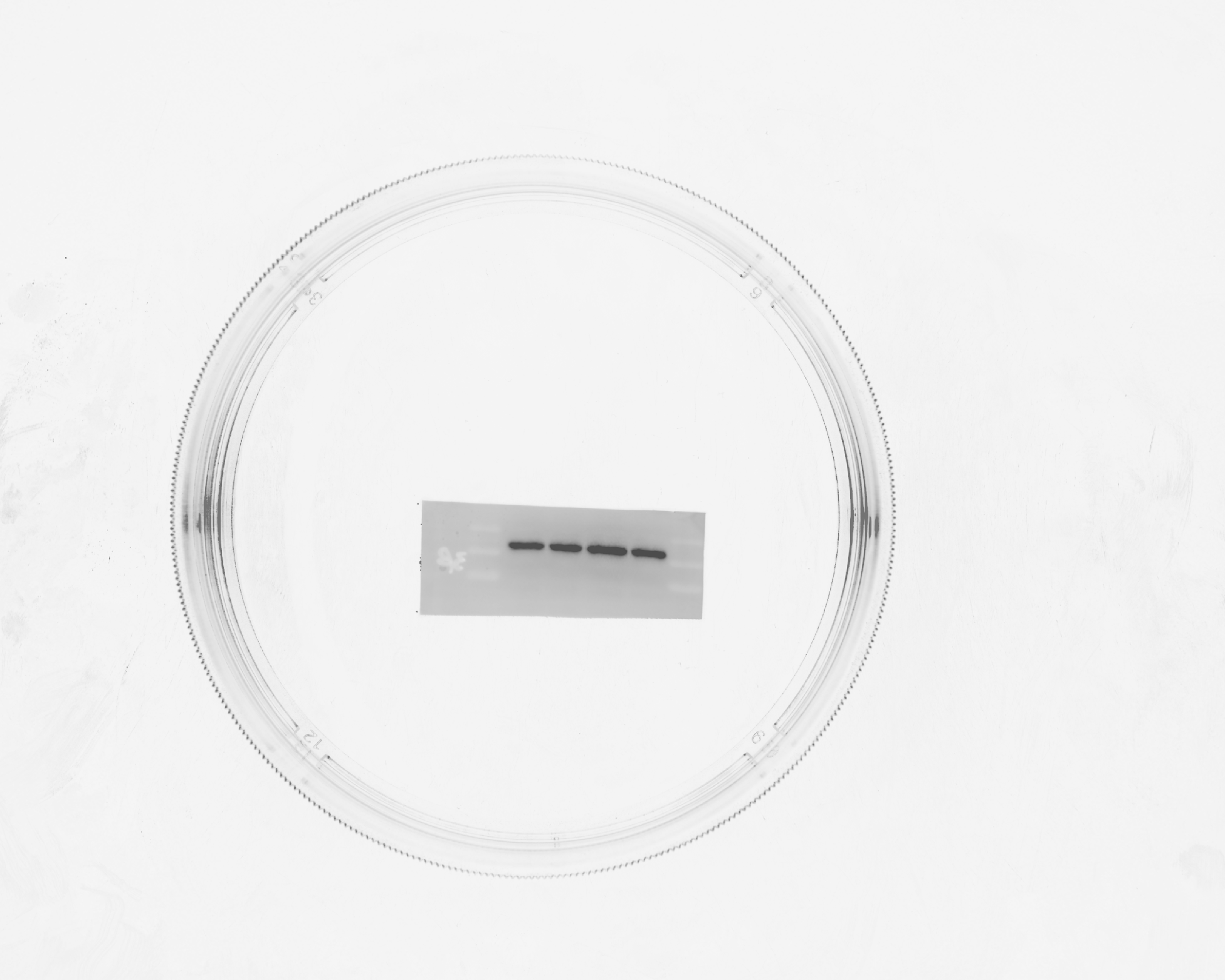

Supplement: Supplementary file 10 [file DataSheet7.zip › 3HIF1α/3-beta-(复合).tif]

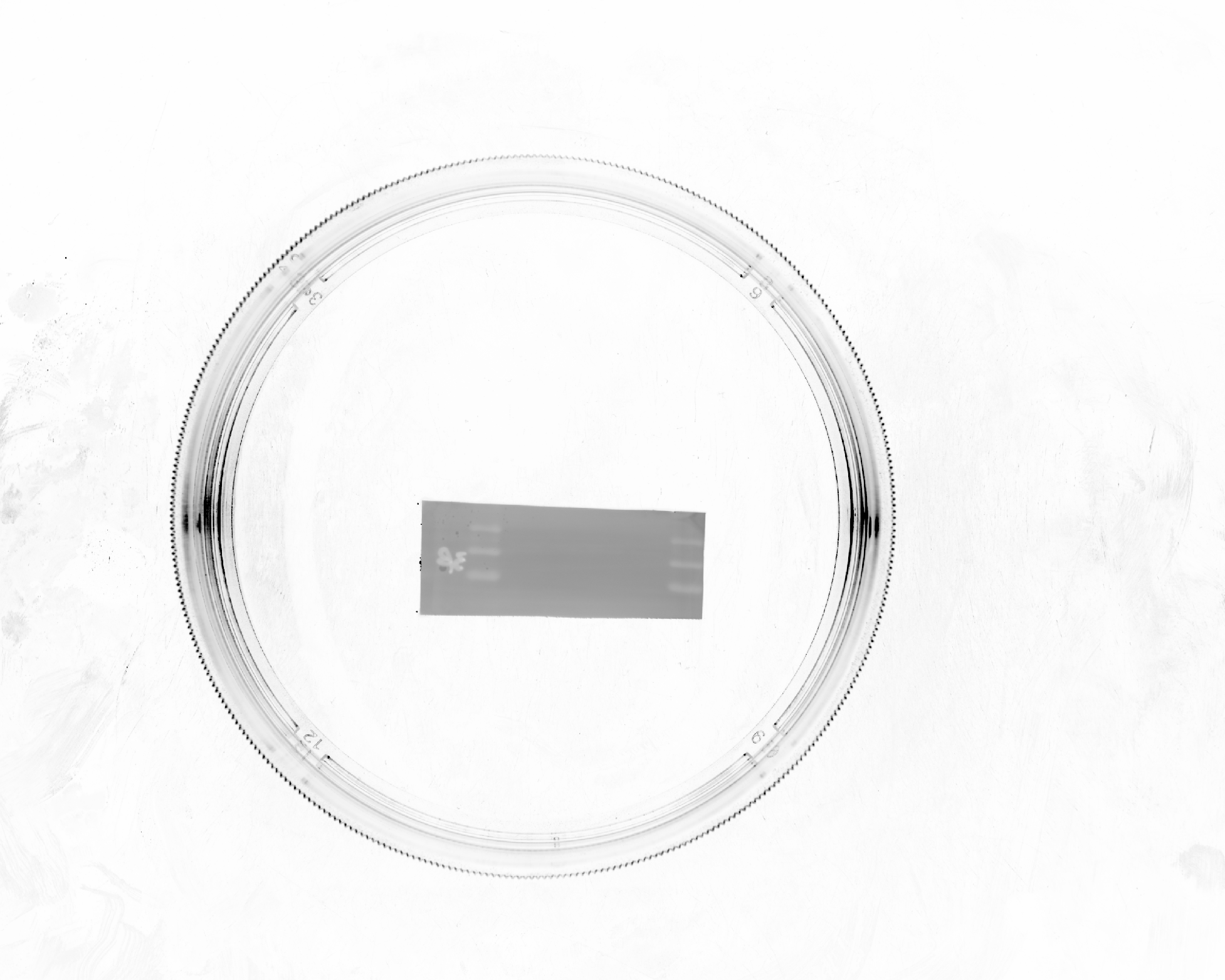

Supplement: Supplementary file 10 [file DataSheet7.zip › 3HIF1α/3-beta--(Colorimetric).tif]

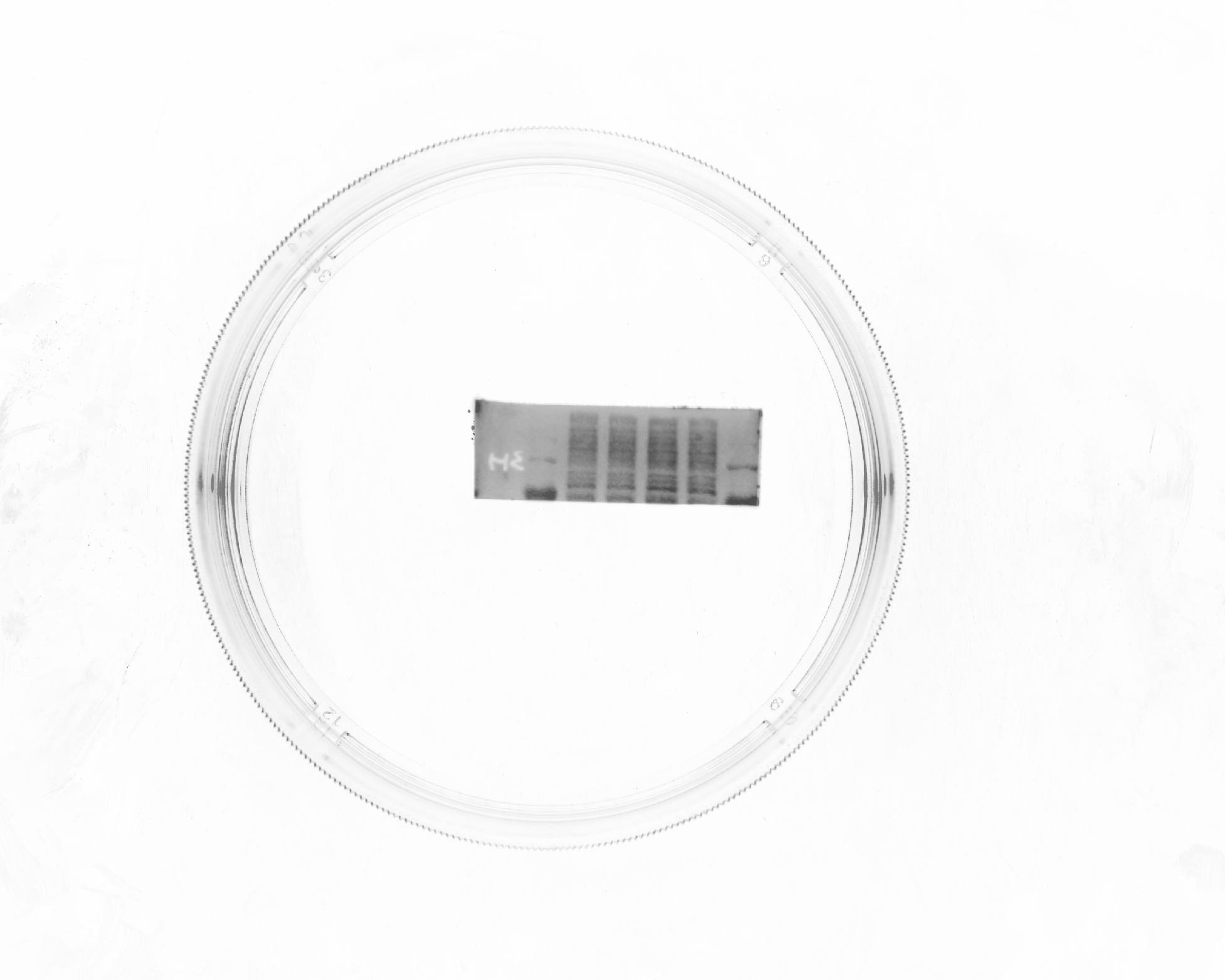

Supplement: Supplementary file 10 [file DataSheet7.zip › 3HIF1α/3-HIF1α(复合).tif]

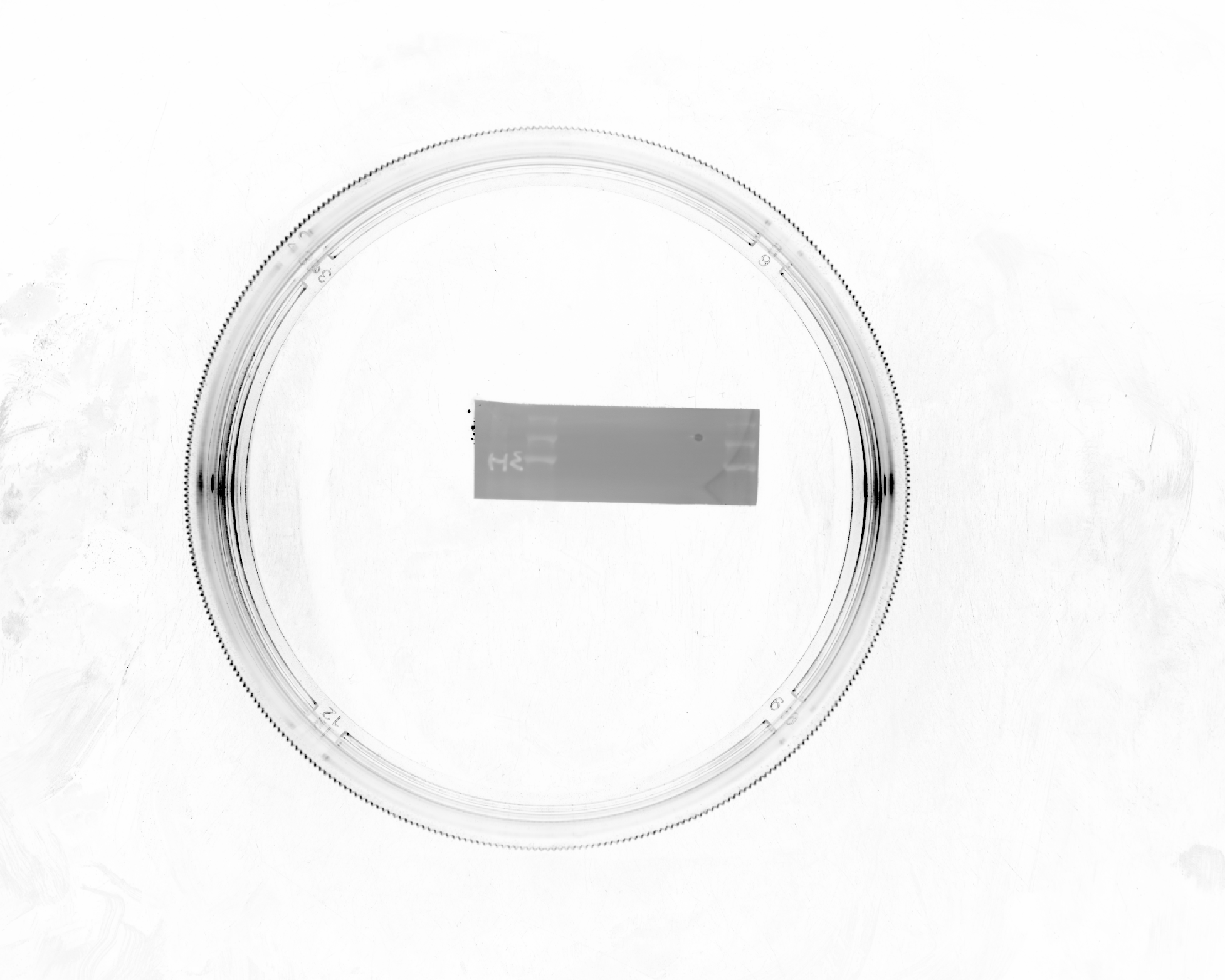

Supplement: Supplementary file 10 [file DataSheet7.zip › 3HIF1α/3-HIF1α-(Colorimetric).tif]

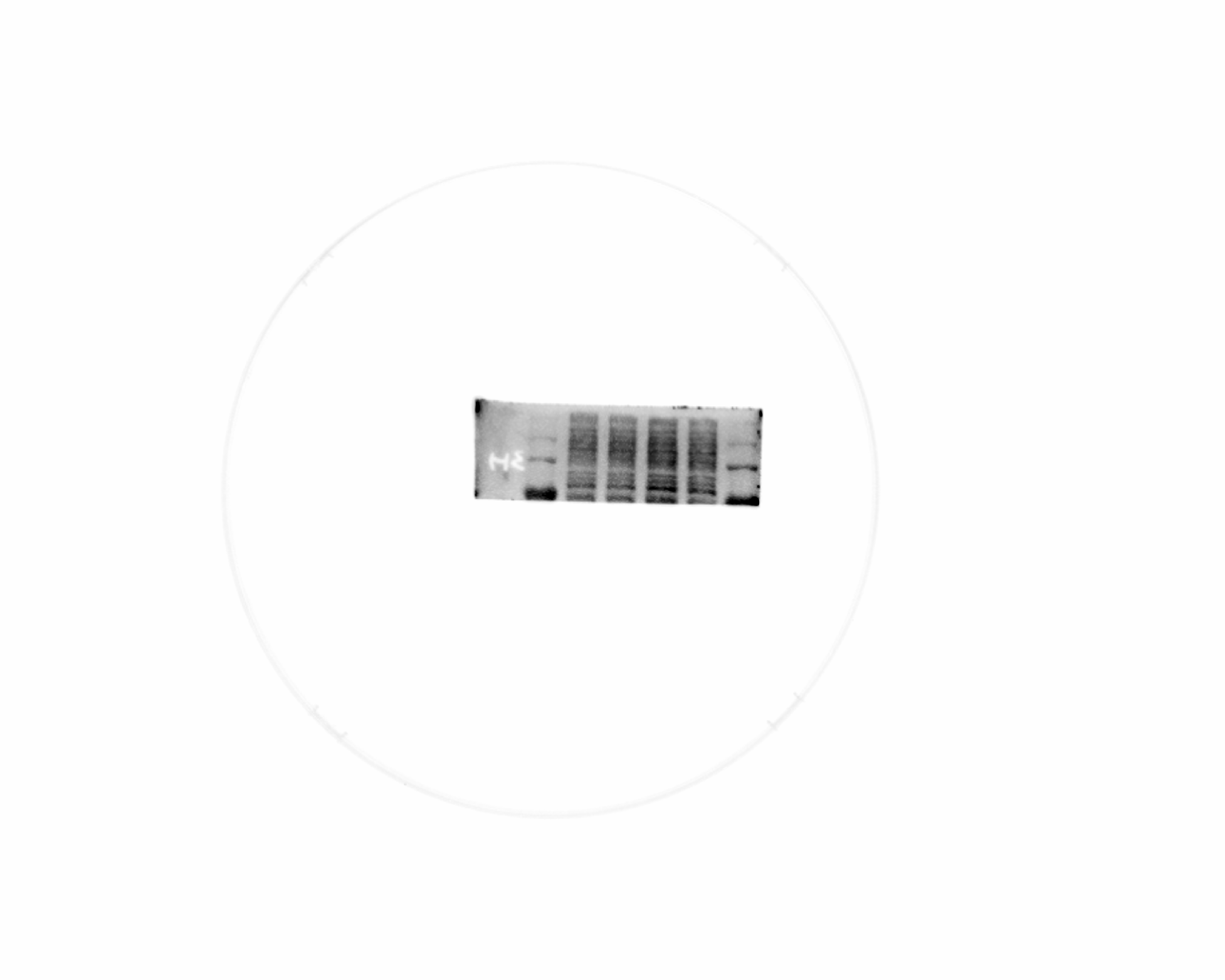

Supplement: Supplementary file 10 [file DataSheet7.zip › 3HIF1α/3-HIF1α--(Chemiluminescence).tif]

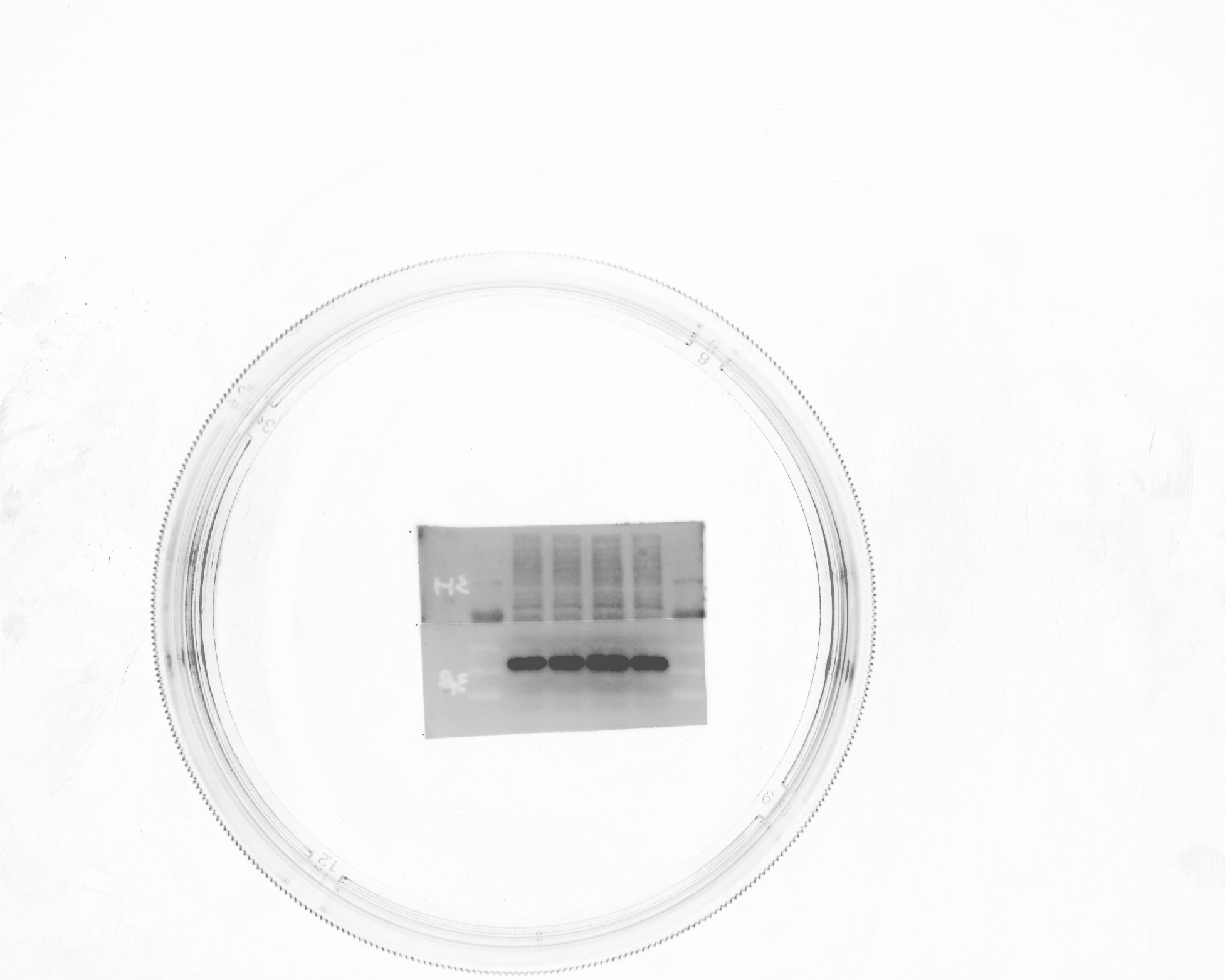

Supplement: Supplementary file 10 [file DataSheet7.zip › 3HIF1α/3HIF1α&1(复合).tif]

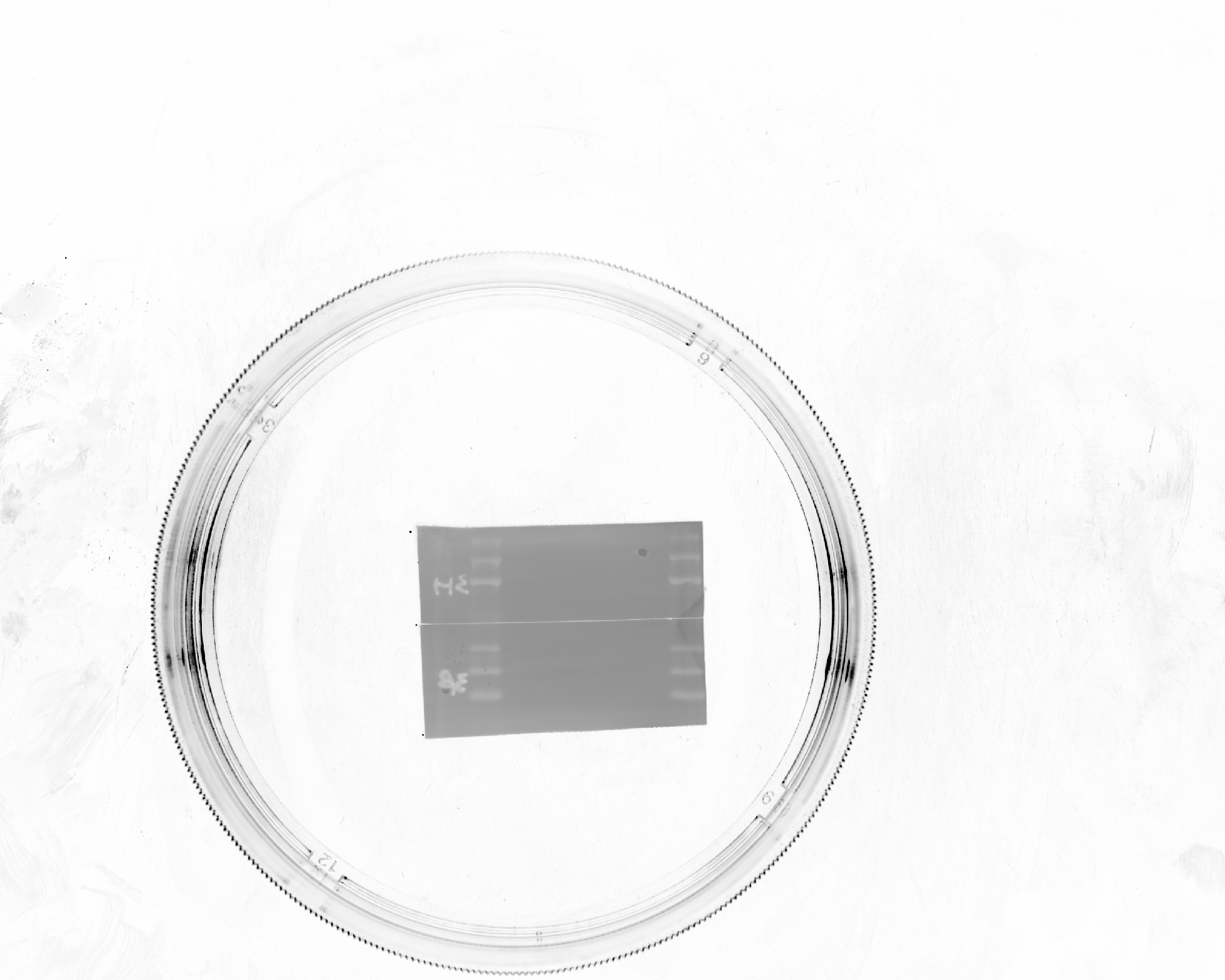

Supplement: Supplementary file 10 [file DataSheet7.zip › 3HIF1α/3HIF1α&1-(Colorimetric).tif]

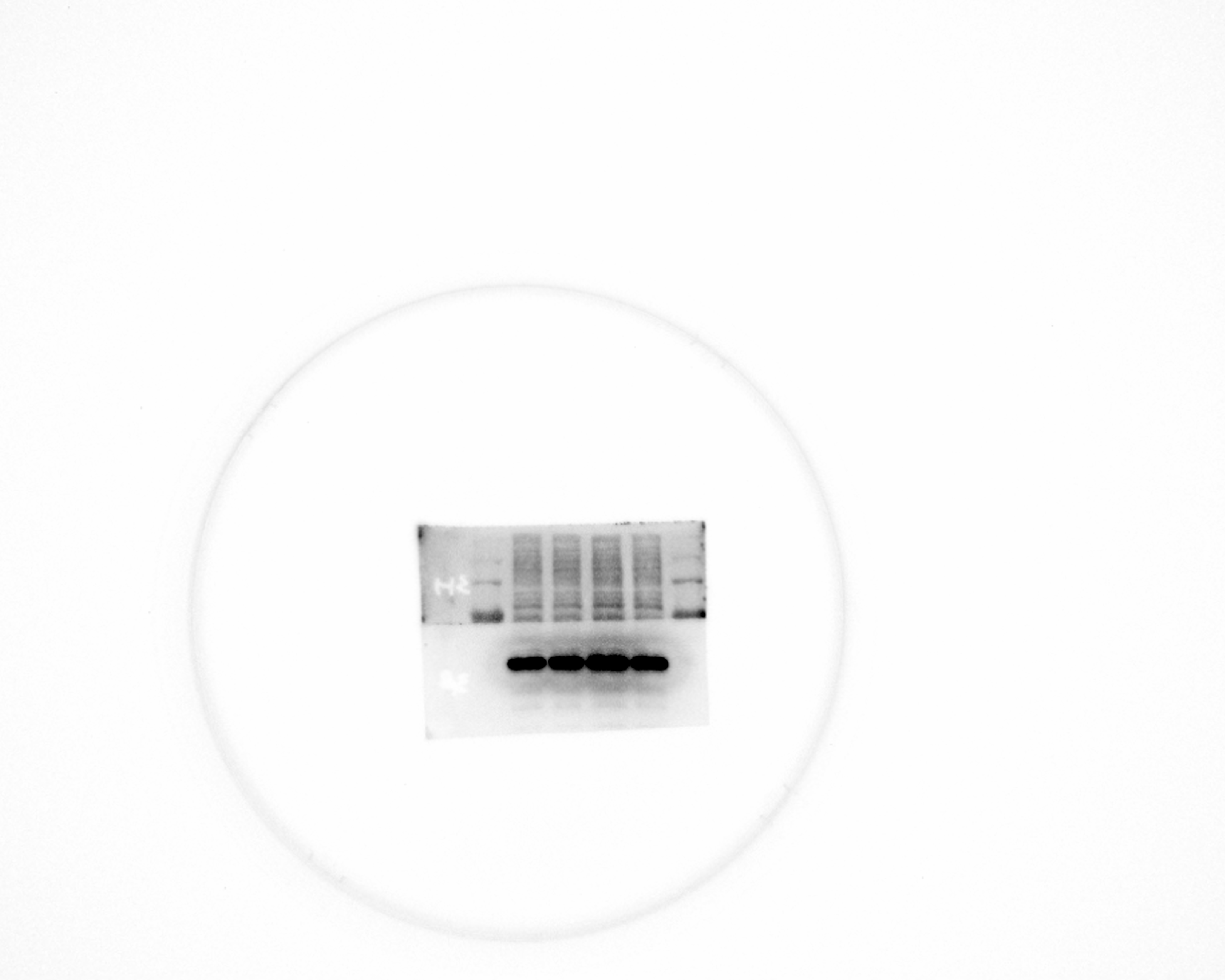

Supplement: Supplementary file 10 [file DataSheet7.zip › 3HIF1α/3HIF1α&beta(Chemiluminescence).tif]
